# Dear-DIA^XMBD^: Deep Autoencoder Enables Deconvolution of Data-Independent Acquisition Proteomics

**DOI:** 10.34133/research.0179

**Published:** 2023-06-26

**Authors:** Qingzu He, Chuan-Qi Zhong, Xiang Li, Huan Guo, Yiming Li, Mingxuan Gao, Rongshan Yu, Xianming Liu, Fangfei Zhang, Donghui Guo, Fangfu Ye, Tiannan Guo, Jianwei Shuai, Jiahuai Han

**Affiliations:** ^1^ Department of Physics, and Fujian Provincial Key Laboratory for Soft Functional Materials Research, Xiamen University, Xiamen 361005, China.; ^2^ Oujiang Laboratory (Zhejiang Lab for Regenerative Medicine, Vision and Brain Health) and Wenzhou Institute, University of Chinese Academy of Sciences, Wenzhou, Zhejiang 325001, China.; ^3^ School of Life Sciences, Xiamen University, Xiamen 361102, China.; ^4^ State Key Laboratory of Cellular Stress Biology, Innovation Center for Cell Signaling Network, Xiamen 361102, China.; ^5^ Department of Computer Science, Xiamen University, Xiamen 361005, China.; ^6^ National Institute for Data Science in Health and Medicine, School of Medicine, Xiamen University, Xiamen 361102, China.; ^7^ Bruker (Beijing) Scientific Technology Co. Ltd., Beijing, China.; ^8^ Westlake Laboratory of Life Sciences and Biomedicine, Key Laboratory of Structural Biology of Zhejiang Province, School of Life Sciences, Westlake University, 18 Shilongshan Road, Hangzhou 310024, China.; ^9^ Institute of Basic Medical Sciences, Westlake Institute for Advanced Study, 18 Shilongshan Road, Hangzhou 310024, China.; ^10^ Westlake Omics Ltd., Yunmeng Road 1, Hangzhou, China.; ^11^ Department of Electronic Engineering, Xiamen University, Xiamen 361005, China.

## Abstract

Data-independent acquisition (DIA) technology for protein identification from mass spectrometry and related algorithms is developing rapidly. The spectrum-centric analysis of DIA data without the use of spectra library from data-dependent acquisition data represents a promising direction. In this paper, we proposed an untargeted analysis method, Dear-DIA^XMBD^, for direct analysis of DIA data. Dear-DIA^XMBD^ first integrates the deep variational autoencoder and triplet loss to learn the representations of the extracted fragment ion chromatograms, then uses the *k*-means clustering algorithm to aggregate fragments with similar representations into the same classes, and finally establishes the inverted index tables to determine the precursors of fragment clusters between precursors and peptides and between fragments and peptides. We show that Dear-DIA^XMBD^ performs superiorly with the highly complicated DIA data of different species obtained by different instrument platforms. Dear-DIA^XMBD^ is publicly available at https://github.com/jianweishuai/Dear-DIA-XMBD.

## Introduction

Mass spectrometry (MS) has long been a dominant technology for peptide and protein identification and quantification [1]. The common strategy for peptide identification is performed by combining the data-dependent acquisition (DDA) approach and database search [2]. Only the top *k* peptide ions with the highest intensity are selected in an MS scan (MS1) for isolation and fragmentation in serial mode for a DDA measurement. The detected fragment ions in MS/MS spectra (MS2) are compared with the theoretical spectra generated by search engines to identify peptides. Nevertheless, the reproducibility of peptides determined by the DDA method is limited because the top *k* precursors are stochastic in repeated DDA experiments.

Aiming to overcome the limitation of the DDA mode, the data-independent acquisition (DIA) strategies have emerged, such as AIF [3], sequential window acquisition of all theoretical mass spectra (SWATH-MS) [4], HDMSE [5], MSX [6], WiSIM-DIA [7], SONAR [8], HRM [9], BoxCar DIA [10], diaPASEF [11], Scanning SWATH [12], and PulseDIA [13]. A common DIA mode is named SWATH-MS, in which all peptide ions in a specified isolation window with a large mass-to-charge ratio (*m*/*z*) are fragmented. The mass spectrometer records all the fragment signals of the mixed peptides in an isolation window. Obviously, it is extremely difficult to directly analyze DIA data because the peptide and fragment signals are mixed in corresponding MS and MS/MS spectra.

In recent years, a number of methods have been developed to process DIA data. For instance, the library-based tools include Spectronaut [9], OpenSWATH [14], SWATHProphet [15], Skyline [16], Specter [17], EncyclopeDIA [18], PIQED [19], DIA-NN [20], and MaxDIA [21]; the library-free tools include DIA-Umpire [22], Group-DIA [23], directDIA (a part of Spectronaut), MSPLIT-DIA [24], PECAN [25], DeepNovo-DIA [26], DIA-NN, and MaxDIA; and the library predicting tools contain DeepMass [27], pDeep [28], Prosit [29], and DeepDIA [30]. OpenSWATH, a prevalent library-dependent workflow integrated into OpenMS [31], was proposed to analyze the SWATH-MS data. OpenSWATH scores the peptides in SWATH-MS data based on the spectral library typically built on DDA MS [32]. To overcome the limitation of DDA library generation, DIA-Umpire calculates the correlation coefficient between precursors and fragments to generate the pseudo-DDA spectra. Group-DIA analyzes the multiple DIA data files simultaneously to determine the precursor–fragment pairs. Both DIA-Umpire and Group-DIA are based on the spectrum-centric strategy. PECAN is a peptide-centric analysis tool that requires a peptide-sequence-based library to directly detect peptides from DIA data. MSPLIT-DIA uses the peptide query method to analyze each DIA MS/MS spectrum. However, the conventional statistical algorithms used by these DIA methods make them insufficient for pattern recognition and classification of extracted ion chromatograms (XICs) of fragments.

In the past 2 years, several deep-learning-based methods have been developed to analyze proteomic MS data [33,34]. DeepNovo-DIA combines the de novo peptide-sequencing method and deep learning to directly identify the amino acid sequences from DIA spectra. DIA-NN begins with a peptide-centric strategy based on in silico spectra libraries and then uses a deep neural network to calculate the *q* value of peptides. DeepDIA predicts MS/MS spectrum and the normalized retention time of peptides in a protein database with a deep learning model and then generates in silico spectral libraries to analyze DIA data. Nevertheless, none of the abovementioned deep-learning-based methods directly analyze DIA data to produce tandem spectra for database searching and then generate the internal libraries (DIA-derived) for quantification. In addition, all of these methods apply supervised learning methods, which limits their generalization ability.

In this paper, we developed Dear-DIA^XMBD^, a spectrum-centric method that combines the deep variational autoencoder (VAE) [35] with other machine learning algorithms to detect the correspondence between precursors and fragments in DIA data without the help of DDA experiments. Dear-DIA^XMBD^ produces the pseudo-tandem MS spectra to a search database and generates the internal libraries. Our approach can be easily integrated into the existing workflow because the output file of Dear-DIA^XMBD^ is in MGF format that can be processed by common search engines, including Comet [36], X!Tandem [37], and MSFragger [38]. Furthermore, benefiting from the fact that the autoencoder is an unsupervised deep learning model, Dear-DIA^XMBD^ shows excellent performance on the DIA data of different species obtained by different instrument platforms. Because of its powerful generalization ability, we suggest that Dear-DIA^XMBD^ is a valuable open-source software for DIA proteomics.

## Results

### Dear-DIA^XMBD^ workflow

**Fig. 1.**
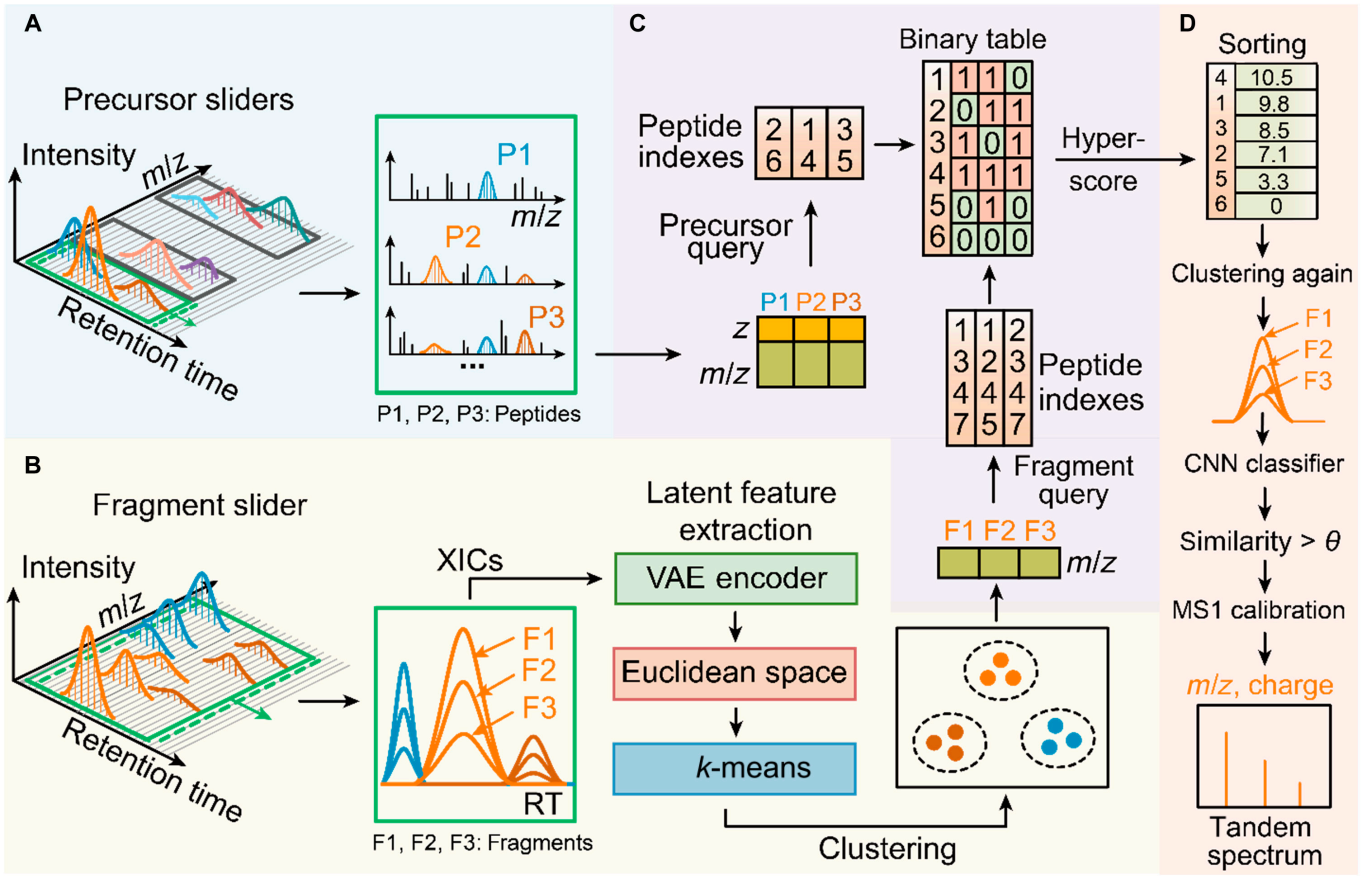
The workflow of Dear-DIA^XMBD^. (A) A precursor slider advanced using set strides along the retention time dimension. The candidate precursors are detected by several SNR-dependent algorithms. (B) The candidate fragment XICs are embedded into the Euclidean space after being fed to the VAE encoder neural network and then assigned to *k*-classes using a *k*-means clustering algorithm. (C) Each fragment cluster is combined with the corresponding precursor based on the protein database and hyperscore. (D) After the high-scoring precursor fragment pairs are removed, the remaining ions are clustered again using *k*-means. These precursor–fragment pairs are judged using a CNN to calculate the similarity among fragments matching the in silico spectrum. The precursor–fragment groups with high similarity are stored as pseudo-tandem spectra for identification.

DIA data are usually visualized as 3-dimensional data containing *m*/*z*, retention time, and intensity. To correctly link the precursors and the ions produced, Dear-DIA^XMBD^ first splits the MS1 retention time with fixed-width sliders in each isolation window. Each precursor slider is treated as a minimum processing unit containing a series of MS1 spectra and corresponding MS2 spectra (Fig. 1A).

Next, we removed the background ions with a low signal–noise ratio (SNR) in the slider using the peak-finding and deisotoping algorithms [39] to determine the candidate precursors and fragments. Since the point-to-point similarity calculation between XICs of candidate fragments is affected by noise and peak misalignment, we used the VAE encoder (Fig. S1) to extract the latent features of fragment XICs and then mapped these features to the Euclidean space. Then, the *k*-means clustering algorithm using a Euclidean metric is applied to assign the candidate fragments to *k* classes in the feature space (Fig. 1B).

Ideally, the fragments in the same cluster should be from the same precursor. In our model, we provide a peptide indexing algorithm named PIndex, which is designed for closed search to return the unique indexes of in silico digested peptides obtained from the FASTA database to determine the precursor of each fragment cluster. A binary table presents the intersection of 2 peptide index sets, that is, the precursor index set obtained from the theoretical identity of the precursor query and the fragment index set obtained from the theoretical identity of the fragment query (Fig. 1C).

We then calculated the hyperscore and sorted the scores for all the in silico digested peptides based on the binary table. Afterward, we removed the precursor–fragment pairs with high scores in the clustering results and then performed *k*-means clustering again on the remaining ions (Fig. 1D). A convolutional neural network (CNN) (Fig. S2) was applied to calculate the similarity among the sets of fragments matching the highest score in silico digested peptide. If the similarity exceeds a certain threshold (θ), the fragments in each cluster will be grouped with the corresponding precursor. We used the high score precursor–fragment groups as internal calibrants to recalibrate all the precursors *m*/*z* [40]. Finally, the calibrated precursor–fragment pairs were stored as the tandem spectrum (Fig. 1D).

### Applications of deep VAE and inverted index

**Fig. 2.**
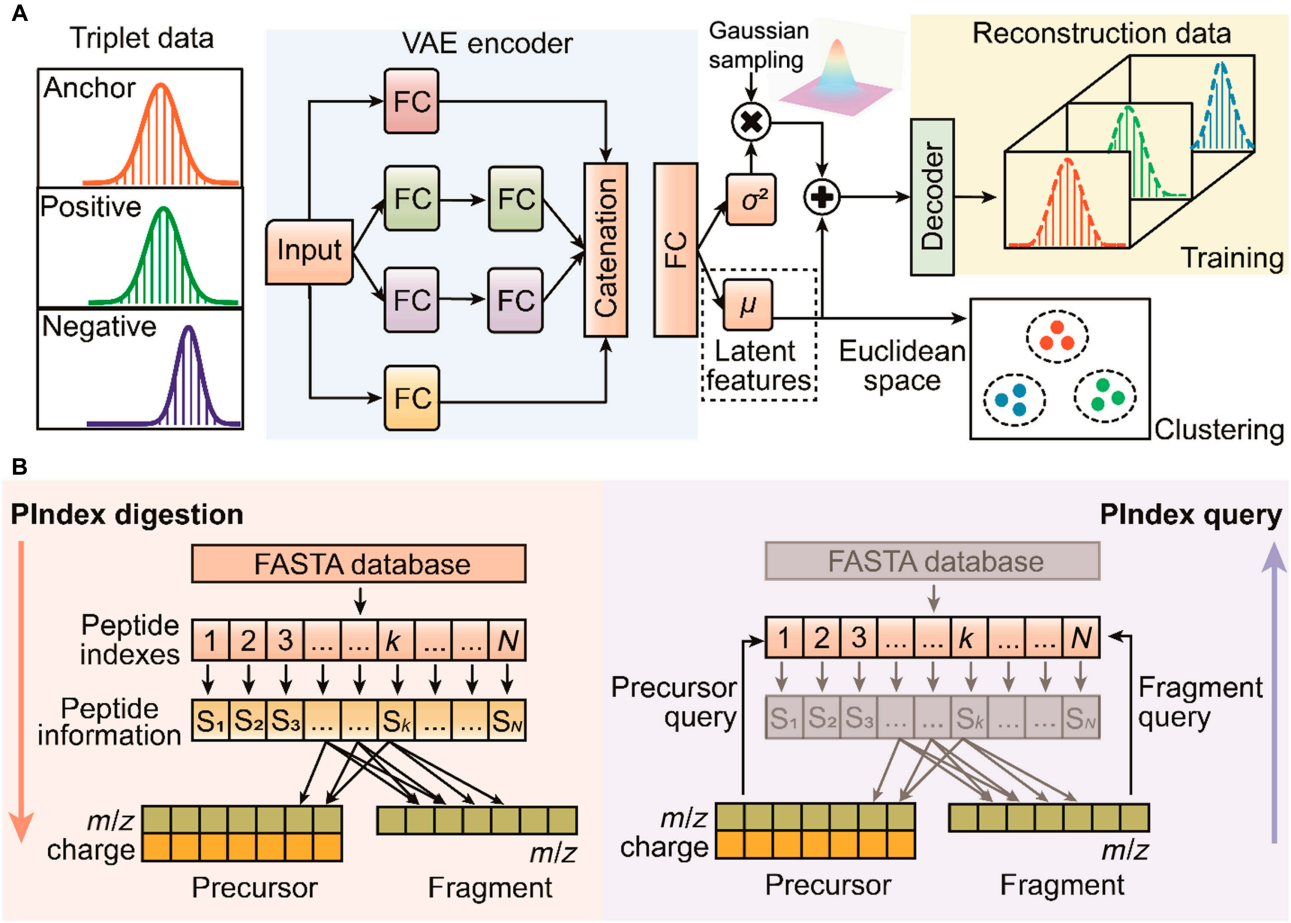
The schematic diagrams of the deep learning model and PIndex querying algorithm. (A) The structure of the deep VAE. The triplet input data contains 3 components: the anchor (red), the positive (green), and the negative (blue) fragment XICs. The anchor and positive fragments come from the same peptide; meanwhile, the anchor and negative fragments belong to different peptides. The triplet data are fed to the 4-branch encoder network, which is consisted of 1-2-2-1 FC layers. The output vectors of the 4-branch networks are catenated by the appending operation at the end. The encoder network outputs 2 vectors of equal size, one for the variance (*σ*^2^) and the other for the mean value (*μ*). The mean vector represents the latent features of the input data. Since anchor and positive are from the same peptide but anchor and negative are from different peptides, the anchor fragment is closer to the positive fragment than to the negative fragment after training triplet loss (the Architecture and training process of VAE section). (B) The peptide indexing algorithm (PIndex). The left part shows the protein database digested into a variety of sets S*_k_*, where *k* indicates the unique index of peptides. The right part describes the processes of precursor query and fragment query. The peptide indexes can be queried by *m*/*z* charge pairs of precursors and *m*/*z* of fragments, respectively.

Because of the high-order complexity of DIA data, the direct classification of the mixed and unlabeled fragment XICs is extremely difficult. Therefore, we designed a VAE model composed of encoder and decoder networks for classification (Fig. 2A). The principle of this model is that the triplet fragment XICs (see details in the Architecture and training process of VAE section) are entered into a multibranched encoder network to extract the latent features of input data for cluster analysis in Euclidean space (Fig. 2A). During the training process, the latent features are reconstructed by a decoder network to make them as close as possible to the input of the encoder (Fig. 2A and see the Architecture and training process of VAE section). We used a loss function of classical VAE with the triplet loss function of FaceNet [41] to improve the ability of the model to distinguish fragments from different precursors. Using a combination of the triplet loss and VAE, we can generate similar representations for fragments of the same precursor and produce the dissimilar features for fragments of the different precursors (Fig. S1 and see the Architecture and training process of VAE section).

Next, we addressed how to precisely match the precursor and fragment clusters. As the narrow-window search is still the main strategy of a database search engine such as MSFragger, Comet, and X!Tandem, an indexing algorithm named PIndex is used to connect the clustering results with the candidate precursors. PIndex contains PIndex digestion and PIndex query algorithms. PIndex digestion begins with an in silico digestion of the protein database containing the series of sets of peptide information S*_k_* with each S*_k_* corresponding to a unique peptide index *k* (Fig. 2B). A peptide information set contains the charge of precursor, the *m*/*z* of precursor, and the *m*/*z* list of fragments. To determine the precursor of each fragment cluster, PIndex constructed precursor query [42] and fragment query for in silico precursors and fragments in all peptide information sets, respectively. Precursor and fragment queries apply the *m*/*z* charge pairs and the *m*/*z* as the key for querying peptide indexes, respectively (Fig. 2B). With the peptide indexes as relay stations, the precursor of each fragment cluster can be quickly inferred.

### A comparison of Dear-DIA^XMBD^ with other DIA analyzing software

To evaluate the performance of Dear-DIA^XMBD^, we make a comparison of Dear-DIA^XMBD^ with the available DIA analysis approaches of DIA-Umpire and Spectronaut 14. First, we trained the autoencoder of Dear-DIA^XMBD^ on an *Escherichia coli* SWATH dataset with 100 variable MS1 windows, which are acquired by TripleTOF 5600 mass spectrometer and TripleTOF 6600 mass spectrometer. The dataset obtained from TripleTOF 5600 contains 7 runs with the MS recording time varying from 30 to 240 min. The datasets from TripleTOF 6600 consist of 6 runs with the MS recording time varying from 15 min to 10 h [43]. We manually selected 97,980 *E. coli* peptide precursor ions quantified by OpenSWATH (Fig. S3B). Each precursor ion contains the top 6 fragment ion XICs. Then, we randomly picked 2 fragment XICs of the same precursor ion as an anchor and positive XICs and randomly selected a fragment XIC from other precursor ions as negative XIC to generate a total of 2,179,590 groups of triplet data as the training dataset (see the Architecture and training process of VAE section). Different from the common supervised deep learning models, we applied the autoencoder to extract the characteristics of XICs, which allows us to use only the number of quantified proteins and peptides as indicators to optimize the model.

We benchmarked the performance of Dear-DIA^XMBD^ using the highly complicated sample datasets, which consist of SWATH-MS Gold Standard (SGS) human dataset [14], L929 mouse dataset, and HYE124 dataset [44] with 64 variable windows (AB Sciex TripleTOF 6600). We used Dear-DIA^XMBD^ to generate pseudo-tandem spectra and then used MSFragger to search the protein FASTA database for peptides and protein identification. All identified peptides and proteins were filtered with a protein-level 1% false discovery rate (FDR) determined by Philosopher [45] to establish the spectrum libraries, and then DIA-NN was applied to quantify peptides and proteins in libraries from Dear-DIA^XMBD^ (Fig. S3A). We applied DIA-Umpire to generate pseudo-tandem spectra and then used the same software tools to process the tandem-spectra file. In addition, we also used Spectronaut 14 (directDIA 2.0) to analyze the benchmark datasets and set the 1% *q* value for filtering the peptides and proteins.

**Fig. 3.**
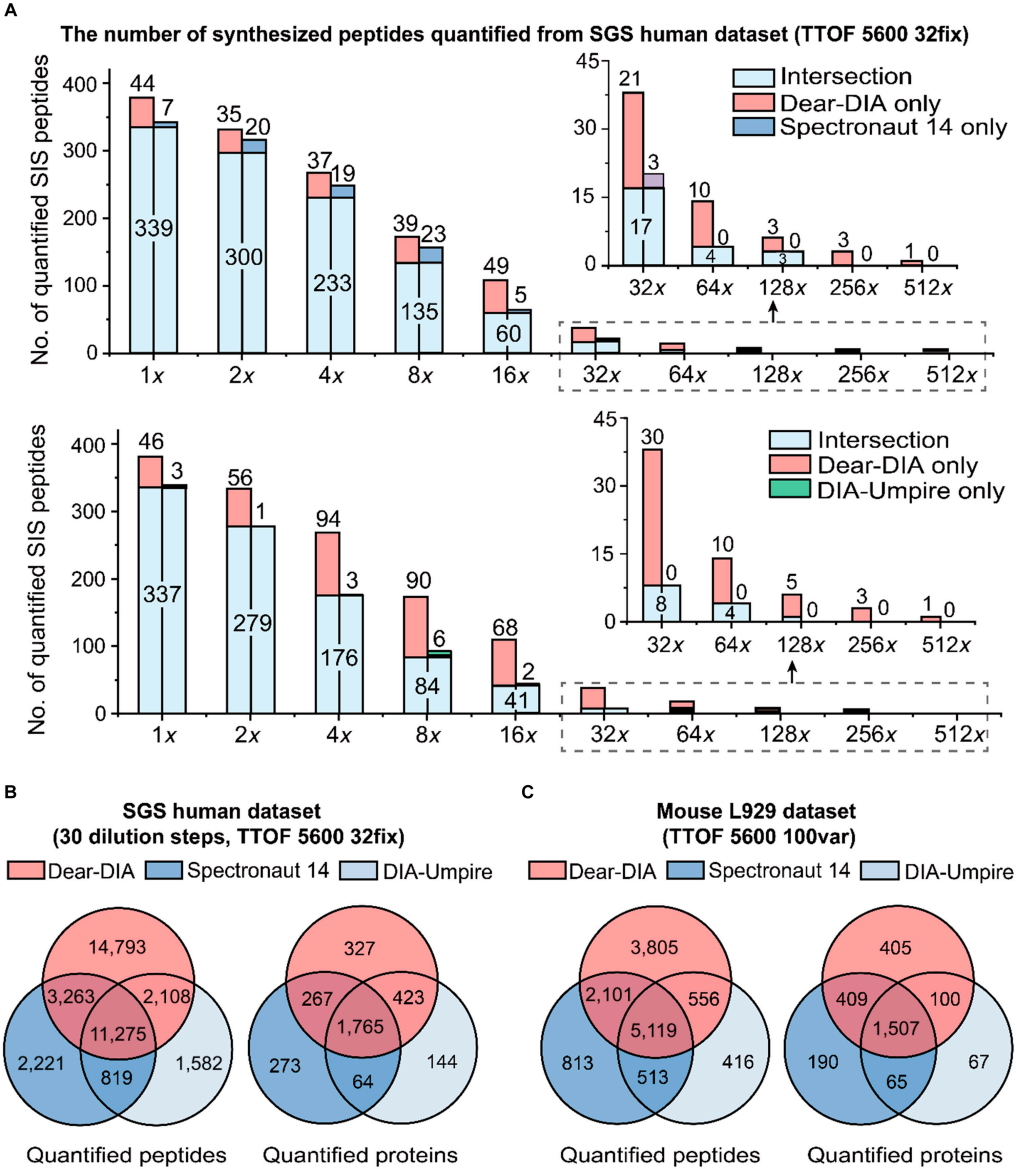
Analysis results of SGS human dataset and mouse L929 dataset. (A) The number of synthesized peptides from the SGS human dataset. The horizontal axis shows the dilution steps from 1× dilution to 512× dilution. The light blue parts in the top and bottom histograms represent the intersection of Dear-DIA^XMBD^ and Spectronaut 14 and the intersection of Dear-DIA^XMBD^ and DIA-Umpire, respectively. The red, dark blue, and green parts show the SIS peptides uniquely found by Dear-DIA^XMBD^, Spectronaut 14, and DIA-Umpire, respectively. (B) Analysis results of all dilution steps (from 1× dilution to 512× dilution), total 30 files, in SGS human dataset. The red, dark blue, and light blue circles represent the results of Dear-DIA^XMBD^, Spectronaut 14, and DIA-Umpire, respectively. (C) Venn diagrams of peptides and proteins found using mouse L929 dataset. The red, dark blue, and light blue circles represent the results of Dear-DIA^XMBD^, Spectronaut 14, and DIA-Umpire, respectively.

The SGS human dataset was generated by Röst et al. [14] using the separately diluted 422 stable isotope-labeled standard (SIS) peptides in HeLa cell lysate in 10 dilution steps (from 1× to 512× dilution) and then acquired as DIA data in triplicate with SWATH-MS. According to the quantified results of SIS peptides, Dear-DIA^XMBD^ can find more synthesized peptides than Spectronaut 14 and DIA-Umpire in all dilution steps, indicating that the sensitivity of Dear-DIA^XMBD^ is higher than Spectronaut 14 and DIA-Umpire (Fig. 3A). Dear-DIA^XMBD^ covers 97% and 98% (average coverage) of SIS peptides reported by Spectronaut 14 and DIA-Umpire, respectively. Notably, the number of SIS peptides uniquely discovered by Dear-DIA^XMBD^ far exceeds those found by Spectronaut 14 and DIA-Umpire, demonstrating that Dear-DIA^XMBD^ shows a higher confidence interval (Fig. 3A). In addition, Dear-DIA^XMBD^ finds more human peptides and proteins than Spectronaut 14 and DIA-Umpire when analyzing 10 dilution steps combined data. According to the quantified results, Dear-DIA^XMBD^ discovered 31,439 peptides and 2,782 proteins, while DIA-Umpire reported 15,784 peptides and 2,396 proteins, and Spectronaut 14 found 17,578 peptides and 2,369 proteins (Fig. 3B and Figs. S4 to S8).

The mouse dataset was derived from L929 cell lysate, which contains triplicate samples with 100 variable MS1 windows measured in SWATH mode on TripleTOF 5600 mass spectrometer (AB Sciex). In the quantification process, the total numbers of peptides found by Dear-DIA^XMBD^, Spectronaut 14, and DIA-Umpire were 11,581, 8,546, and 6,604, respectively, and the total number of proteins found by Dear-DIA^XMBD^, Spectronaut 14, and DIA-Umpire were 2,421, 2,171, and 1,739, respectively. Dear-DIA^XMBD^ also covers 84.5% of peptides and 88.3% of proteins reported by Spectronaut 14. Dear-DIA^XMBD^ covers 85.9% of peptides and 92.4% of proteins revealed by DIA-Umpire. The wide coverage shows a nice reproducibility among Dear-DIA^XMBD^, DIA-Umpire, and Spectronaut 14 (Fig. 3C and Figs. S4 and S9). Dear-DIA^XMBD^ discovered more low-intensity peptides than DIA-Umpire (Fig. S10).

Next, we compare the performances of Dear-DIA^XMBD^, DIA-Umpire, and Spectronaut 14 with the HYE124 dataset, which was specifically designed for checking DIA algorithms. The HYE124 dataset includes 2 hybrid proteome samples, A and B. Sample A was composed of 65% (w/w) human, 30% (w/w) yeast, and 5% (w/w) *E. coli* proteins, while sample B was composed of 65% (w/w) human, 15% (w/w) yeast, and 20% (w/w) *E. coli* proteins.

**Fig. 4.**
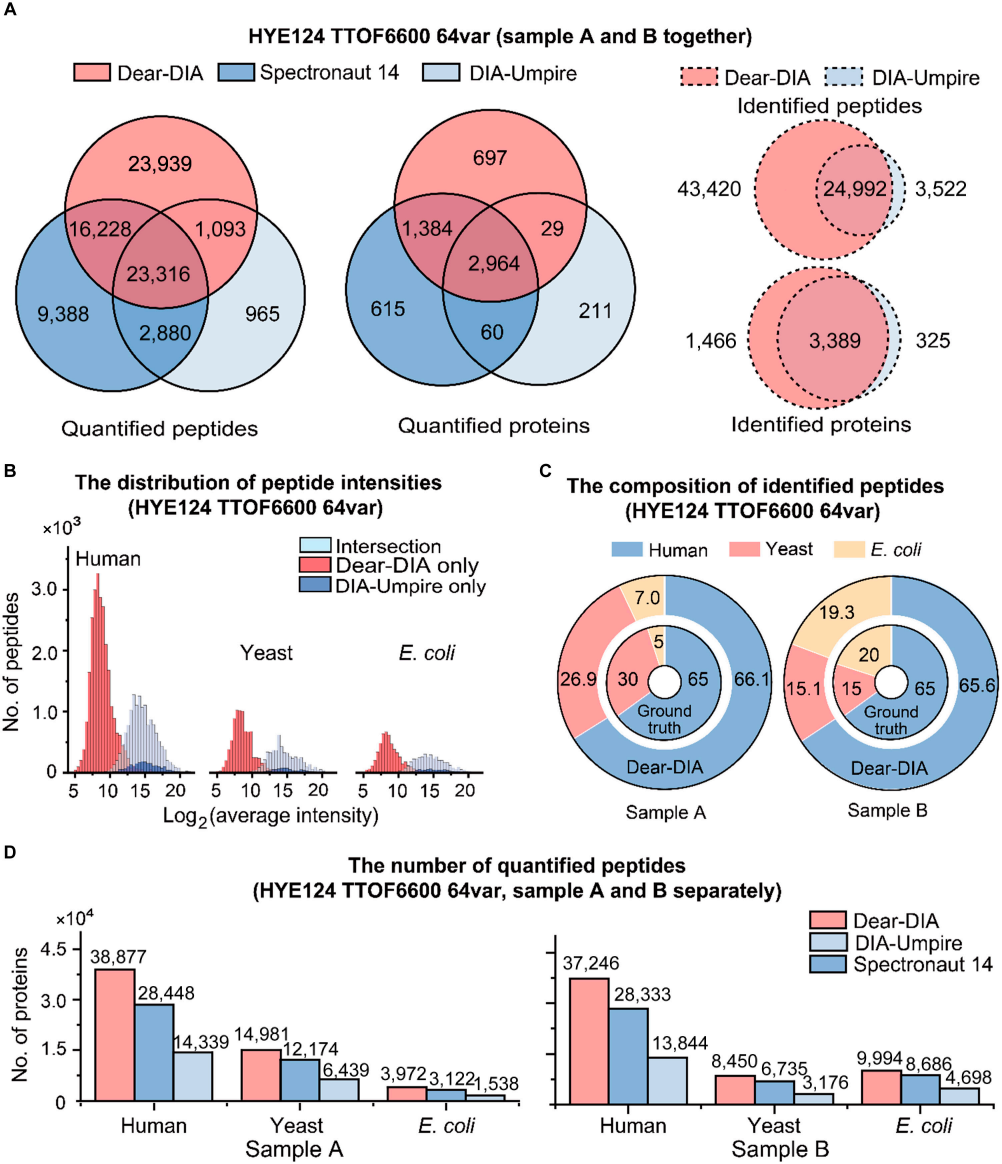
Analysis results of HYE124 dataset with 64 variable windows (TripleTOF 6600). (A) The comparison of numbers of identified and quantified peptides and proteins obtained by Dear-DIA^XMBD^, DIA-Umpire, and Spectronaut 14 from the HYE124 dataset with samples A and B together. The solid lines and the dashed lines show the quantified and identified results, respectively. The red, dark blue, and light blue circles represent the results of Dear-DIA^XMBD^, Spectronaut 14, and DIA-Umpire, respectively. (B) The log_2_-scaled distributions of the quantified peptide intensities were discovered from the HYE124 dataset with samples A and B together. The peptides shared jointly with DIA-Umpire and Dear-DIA^XMBD^ are shown in light blue; the peptides exclusively reported by Dear-DIA^XMBD^ and by DIA-Umpire are shown in red and dark blue, respectively. (C) The composition of proteins found by Dear-DIA^XMBD^ with sample A dataset and with sample B dataset, respectively. The blue, red, and yellow colors represent human, yeast, and *E. coli* species, respectively. The smaller doughnuts represent the ground-truth composition of proteins, which are mixed in defined proportions. The larger doughnuts show the composition of proteins discovered by Dear-DIA^XMBD^. (D) The numbers of the quantified proteins found by Dear-DIA^XMBD^, Spectronaut 14, and DIA-Umpire with sample A dataset and sample B dataset, respectively. The red, dark blue, and light blue colors show the results of Dear-DIA^XMBD^, Spectronaut 14, and DIA-Umpire, respectively.

Adding 2 samples of HYE124 datasets together, the total quantified peptides discovered by Dear-DIA^XMBD^, Spectronaut 14, and DIA-Umpire are 64,576, 51,812, and 28,254, respectively, and the total quantified proteins are 5,074, 5,023, and 3,264, respectively, in which Dear-DIA^XMBD^ covers 86.6% proteins and 76.3% peptides found by Spectronaut 14. Dear-DIA^XMBD^ also covers 91.7% proteins and 86.4% peptides found by DIA-Umpire (Fig. 4A). These results show quite a good reproducibility among Dear-DIA^XMBD^, Spectronaut 14, and DIA-Umpire. In addition, the number of identified peptides discovered uniquely by Dear-DIA^XMBD^ was 12.3 times that found uniquely by DIA-Umpire (i.e., 43,420 versus 3,522) (Fig. 4A and Figs. S11 to S13). Dear-DIA^XMBD^ can find a large number of proteins and peptides overlooked by DIA-Umpire in identification and quantification. The current Dear-DIA^XMBD^ only uses the *E. coli* data as training data, but it shows excellent generalization ability.

Furthermore, it is well known that proteins and peptides with low abundance are hardly identified by MS analysis algorithms because of the interference of background noise. However, Dear-DIA^XMBD^ performs much better than DIA-Umpire on this issue when using the same quantified software tool such as DIA-NN since the intensity distributions of the quantified proteins and peptides given by Dear-DIA^XMBD^ are more in the low-density range (Fig. 4B).

We also analyzed sample A and sample B of the HYE124 dataset separately. For sample A, the percentages of identified peptides given by Dear-DIA^XMBD^ were 66.1% for humans, 26.9% for yeast, and 7.0% for *E. coli*, respectively. For sample B, Dear-DIA^XMBD^ found 65.6% of humans, 15.1% of yeast, and 19.3% of *E. coli*-identified peptides, respectively (Fig. 4C). Consistently, Dear-DIA^XMBD^ found more peptides in humans, yeast, and *E. coli* than Spectronaut 14 and DIA-Umpire (Fig. 4D). We manually checked the XICs of human, yeast, and *E. coli* peptides identified by Dear-DIA^XMBD^ (but not DIA-Umpire and Spectronaut 14) to confirm the similarity among fragments (Fig. S14).

**Fig. 5.**
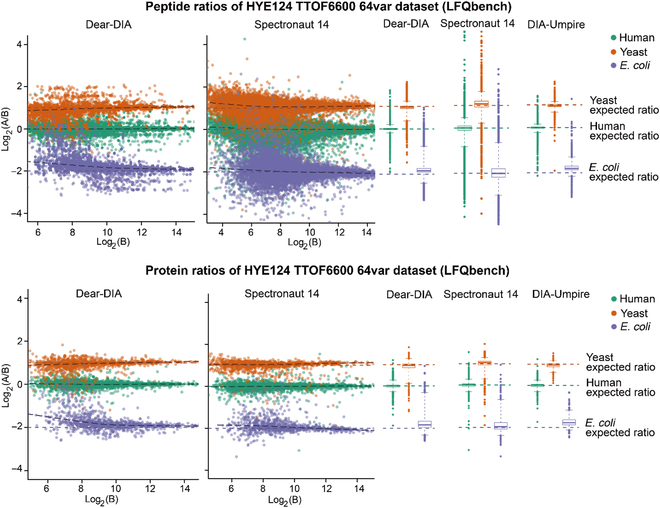
Peptide-level and protein-level LFQbench test performance of HYE124 dataset with 64 variable windows (TripleTOF 6600). The top and bottom scatter plots represent the peptide ratios and the protein ratios reported by Dear-DIA^XMBD^ and Spectronaut14, respectively. The colored dashed lines indicate the expected log_2_(A/B) ratios for human (green), yeast (orange), and *E. coli* (purple) species. The black dashed lines represent the local trend along the *x* axis of the experimental log-transformed ratios of each population (human, yeast, and *E. coli*). The horizontal axis and vertical axis of the scatter chart represent the log-transformed ratios [log_2_(A/B)] of the quantified intensity and the log-transformed intensity of sample B [log_2_(B)], respectively. The top and bottom box plots show the quantified performance of peptides and proteins, respectively (boxes, interquartile range; whiskers, 1 to 99 percentile; human, yeast, and *E. coli*).

We used LFQbench [44] R package to benchmark the precision of quantification on the HYE124 dataset. Compared with Spectronaut 14 and DIA-Umpire, Dear-DIA^XMBD^ performs similarly in precision for both peptides and proteins of humans, yeast, and *E. coli* (Fig. 5, Fig. S15, and Table S1). We also tested the performance of Dear-DIA^XMBD^ on the HYE124 TripleTOF 5600 dataset with 64 variable windows (Figs. S16 to S20). Dear-DIA^XMBD^ discovered more peptides and proteins than Spectronaut 14 and DIA-Umpire.

In addition, we used Dear-DIA^XMBD^, Spectronaut 14, and DIA-Umpire to analyze the Biognosys facility (BGS) mouse DIA dataset [46], which was acquired from Orbitrap Fusion Lumos mass spectrometer (Thermo Fisher Scientific, San Jose, CA) with a 2-h gradient and 40 DIA scans. The results demonstrated that Dear-DIA^XMBD^ could also analyze the data from Thermo Fisher Scientific mass spectrometer (Fig. S21). We conducted comparisons and performance evaluations of Dear-DIA^XMBD^ from various perspectives (Texts S1 to S7) and provided a detailed usage tutorial of Dear-DIA^XMBD^ (Text S8).

We also benchmarked Dear-DIA^XMBD^ on TNFR1 (tumor necrosis factor receptor 1) complex dataset [23,47–50] from L929 cells treated with TNF from 6 different time periods. We performed a comparison of DIA-NN quantified results and manual inspection results, showing that Dear-DIA^XMBD^ can find truly regulated proteins (Fig. S22).

## Discussion

In the paper, we designed a new method with neural network architecture, namely, Dear-DIA^XMBD^, to improve the feature extraction ability of fragment XICs, which consults to the structures of a fully connected (FC) VAE network. Moreover, we also implemented a high concurrency program written in C/C++ from scratch to increase the speed of the program.

We demonstrated that Dear-DIA^XMBD^ is a more efficient method than DIA-Umpire and Spectronaut 14 in directly analyzing DIA data to discover proteins and peptides. First of all, Dear-DIA^XMBD^ can reproduce most of the results obtained by DIA-Umpire and Spectronaut 14 at identification and quantification levels. Second, Dear-DIA^XMBD^ can identify more low-abundance proteins and peptides, indicating that it has a better performance than DIA-Umpire in processing the low SNR signals.

Furthermore, although the training dataset is from *E. coli*, Dear-DIA^XMBD^ shows an outstanding performance in analyzing datasets of different species with different instruments, indicating its general recognition ability. The pseudo-tandem spectra generated by Dear-DIA^XMBD^ can be easily fed into common search engines for library generation. In addition, analyzing MS data of posttranslational modifications (PTMs) is an important issue and challenge. Dear-DIA^XMBD^ currently supports carbamidomethyl as a fixed modification and oxidation and *n*-acetylation as variable modifications. Dear-DIA^XMBD^ does not work accurately enough for other modifications such as phosphorylation. Since OpenSWATH IPF [51] is a powerful tool for processing PTMs data, we consider combining Dear-DIA^XMBD^ and OpenSWATH IPF to analyze PTMs DIA data in future work.

Collectively, Dear-DIA^XMBD^ is an advanced software for processing a variety of highly complex DIA data. We believe that deep learning methods may play more important roles in the analysis of the complicated protein spectrum data.

## Materials and Methods

### Training data for deep learning

*E. coli* DH5a strain cells were washed 3 times with H_2_O and collected by centrifugation. Protein pellet was dissolved in 1% sodium deoxycholate (SDC)/10 mM tris(2-carboxyethyl)phosphine hydrochloride (TCEP)/40 mM 2-chloroacetamide (CAA)/tris-HCl (pH 8.5). Subsequently, 1% SDC was diluted with water to 0.5%. The protein centration was measured with Pierce 660-nm protein assay reagent (Thermo Fisher Scientific). The trypsin (Sigma-Aldrich) was added with the ratio of 1:100 (trypsin:protein). The tubes were kept at 37 °C for 12 to 16 h. The peptides were desalted with poly(styrene-divinylbenzene)-reversed phase sulfonate (SDB-RPS) StageTips. Peptides were dissolved in 0.1% formic acid (FA; 06440, Sigma-Aldrich) and analyzed by TripleTOF 5600 MS (AB Sciex). Peptides first bound to a 5-mm × 300-μm trap column packed with Zorbax C18 5-μm 300-Å resin (5065-9913, Agilent) using 0.1% (v/v) FA/2% acetonitrile (ACN) in H_2_O at 10 μl/min for 5 min, and then separated using 30-, 45-, 60-, 120-, 150-, 180-, or 240-min gradient from 2% to 35% buffer B [buffer A: 0.1% (v/v) FA and 5% dimethyl sulfoxide (DMSO) in H_2_O; buffer B: 0.1% (v/v) FA and 5% DMSO in acetonitrile) on a 30-cm × 75-μm in-house pulled emitter-integrated column packed with Magic C18 AQ 3-μm 200-Å resin. The column temperature was kept at 50 °C by a column heater (PST_CHC-RC, Phoenix S&T) and a controller (PST-BPH-20, Phoenix S&T). For SWATH-MS, MS1 scan recorded a 350 to 1,250 *m*/*z* range for 250 ms, and a 100 to 1,800 *m*/*z* range was recorded for 33.3 ms in the high-sensitivity mode MS2 scan. One MS1 scan was followed by 100 MS2 scans, which covered a precursor *m*/*z* range from 400 to 1200.

### Sample preparation and MS analysis of L929 mouse datasets

Murine fibroblast L929 cells were harvested by scraping and centrifugation at 4 °C. L929 cells were lysed with 1% SDC/100 mM tris-HCl (pH 8.5), followed by sonication. The protein concentration was assayed using the bicinchoninic acid (BCA) method. Ten micrograms of proteins were reduced and alkylated using 10 mM TCEP/40 mM CAA. One percent of SDC was diluted to 0.5% SDC using high-performance liquid chromatography H_2_O, and trypsin was added at the protein: trypsin ratio of 50:1. Digestion was performed at 37 °C for 12 and 16 h. The tryptic peptides were cleaned up using SDB-RPS StageTips before MS analysis. Peptides were dissolved in 0.1% FA and analyzed by MS in DDA and SWATH modes. MS analysis was performed on a TripleTOF 5600 (Sciex) MS coupled to NanoLC Ultra 2D Plus (Eksigent) high-performance liquid chromatography system. Peptides first bound to a 5-mm × 500-μm trap column packed with Zorbax C18 5-μm 200-Å resin using 0.1% (v/v) FA/2% acetonitrile in H_2_O at 10 μl/min for 5 min and then separated from 2% to 35% buffer B (buffer A: 0.1% (v/v) FA and 5% DMSO in H_2_O; buffer B: 0.1% (v/v) FA and 5% DMSO in acetonitrile) on a 15-cm × 75-μm in-house pulled emitter-integrated column packed with Magic C18 AQ 3-μm 200-Å resin. For DDA, the 250-ms MS1 scan was performed in the range of 350 to 1,250 *m*/*z*, and up to 20 most intense precursors with charge states 2 to 5 were isolated for fragmentation. MS/MS spectra were collected in the range of 100 to 1,800 *m*/*z* for 100 ms. For SWATH-MS, a 100-ms survey scan (time-of-flight MS) that was collected in 350 to 1,500 *m*/*z* was performed followed by 100 MS2 experiments with scan time of 33 ms, which were collected in 100 to 1,800 *m*/*z*. The 100 variable isolation windows of L929 dataset were the same as those of *E. coli* dataset acquired from TripleTOF 5600.

### The complete analysis workflow of Dear-DIA^XMBD^

The complete analysis workflow of Dear-DIA^XMBD^ mainly consists of identification and quantification processes. The workflow begins with a profile mzXML file and ends with a report file contained peptides and proteins. In the identification process, the raw files of MS data were converted into profile mzXML files using MSConvert (V.3.0.19311), which were subjected to Dear-DIA^XMBD^ for generating pseudo-DDA mgf files (Fig. S3A). The mgf files were converted to mzML files, which were analyzed with FragPipe (version 19.1) workflow. The mzML files were subjected to database search using MSFragger search engine (version 3.7) against an UniprotKB/Swiss-Prot database (Fig. S3A).

The pepXML search results were validated and scored using PeptideProphet [52], PTMProphet [53], and ProteinProphet [54] modules integrated into the Philosopher toolkit (version 4.8.1). For PeptideProphet, the following parameters were utilized: --decoyprobs --ppm --accmass --nonparam --expectscore. PTMProphet used default parameters, including KEEPOLD STATIC EM=1 NIONS=b STY:79.966331, M:15.9949 MINPROB=0.5. ProteinProphet was configured with the parameter --maxppmdiff 2000000. Subsequently, the validated report files were filtered at the protein-level 1% FDR using Philosopher filter module, with the parameters --sequential --prot 0.01 --tag DECOY_. The target peptide ions passing the 1% FDR threshold were used as input for EasyPQP to generate a spectral library. In the library, the retention time of peptides was replaced with normalized retention time, and endogenous peptides were used for retention time normalization (Fig. S23).

For the quantification process, DIA-NN (version 1.8.1) was used as quantified toolkit to analyze the raw MS data. The above library file was used as the specific spectral library of DIA-NN instead of the in silico spectral library generated from FASTA database. The remaining parameters of DIA-NN were set to their default values.

### Parameters of software tools

The parameters of MSFragger and DIA-NN are shown in Table S2 and Fig. S24. The parameters of Dear-DIA^XMBD^, Spectronaut 14 (v14.10.201222.47784 and v14.9.201124.47784) and DIA-Umpire (v2.3.2) are shown in Tables S3 to S5, respectively.

### Format conversion of benchmarked datasets

The .wiff raw data files were converted into profile and centroid mzML and mzXML format by msconvert.exe and qtofpeakpicker.exe from ProteoWizard (version 3.0.20039) package. The .raw files from Thermo Fisher Scientific mass spectrometer were converted into mzXML by msconvert.exe (ProteoWizard version 3.0.20039).

### Data preprocessing

Usually, DIA data contain a large number of background ion signals, which greatly increases the data redundancy and complexity. Thus, we applied several preprocessing algorithms to reduce the calculation consumption. In an MS1 isolation window, Dear-DIA^XMBD^ uses a fixed-width slider in MS1 retention time dimension to capture the local characteristics of DIA data. A slider contains a series of precursor ion spectra and the corresponding fragment ion spectra. Alignment of fragment XICs can be naturally resolved by using sliders in a single run. The fixed width of slider was set as 20, which is the length of XIC. Under the parallel mode, we moved the slider of all MS1 windows with stride of one and update the internal MS1 and MS2 spectra. By setting the appropriate width of slider, we assume that the peptides in a slider are recorded only once, and the chromatographic peaks of fragments from the same peptide show similar shapes.

Considering that precision of MS data in profile mzXML file as high as 10^−3^, Dear-DIA^XMBD^ applied the binning algorithm to truncate the precision MS data to low-precision values for spectrum analysis. In detail, we split 1.0 *m*/*z* to 30 bins with a truncated resolution of 0.03 *m*/*z*. The number of bins is a configurable parameter for the users. As a result, each spectrum in slider is represented by a fixed-length vector after data binning. For instance, if the maximum value of *m*/*z* is specifically defined as 1,200, the spectrum will be represented by a vector of length 36,000. An index of the vector corresponds to *m*/*z* of an ion, and the vector value at that index is equal to the ion intensity. If an ion is not recorded in a spectrum, its intensity will be replaced by zero. The binning algorithm starts from the zero value of initial vector. Then, the ion intensity is accumulated to the vector values of the corresponding index. Both MS1 and MS2 spectra are handled by this binning method.

Because the signals of precursors and fragments are submerged in a large number of background ion signals, the binned DIA data are still complicated. It is important to remove the background ions with an extremely low SNR. Furthermore, in SWTAH workflow, MS1 and MS2 scan times are configured to 250 and 33 ms, respectively. The difference in scan time causes the SNR of MS1 spectrum to be higher than that of MS2 spectrum. Therefore, Dear-DIA^XMBD^ adopts different methods to filter the background ions in MS1 and MS2 spectra.

For each MS1 spectrum, a peak-finding algorithm is applied to detect peaks with high SNR in *m*/*z* dimension. Those detected peaks are probably from the true signals of peptides, rather than background ions. For the detected peaks, a deisotoping algorithm is then used to find the isotopic clusters and to calculate the ion charge of the first isotopic peak. The ions that are able to determine the ion charges are regarded as the candidate precursors in a slider. Dear-DIA^XMBD^ stores XICs, charges, and the binning *m*/*z* indexes of the candidate precursor ions.

Furthermore, the background ions in MS2 spectra are handled by setting 2 filter conditions of SNR of XICs. The first requirement is that the number of nonzero values of XIC must be greater than 5. The second condition requires that the ratio of the maximum value to the nonzero minimum value of XIC should be larger than 4. All fragments satisfying these conditions are treated as the candidate fragments in a slider, and their *m*/*z* and XICs are stored for the next processing.

### Feature extraction of fragment XICs

We feed fragment XICs into the encoder network and store the output of encoder as the representation of XICs. The deep neural network is written by Python3.6 on MXNet deep learning framework and trained on NVIDIA GeForce GTX 1080Ti GPU.

### Architecture and training process of VAE

Autoencoders are important unsupervised learning models for data dimensionality reduction and feature extraction. Their learning objectives perform the following mapping:

$$\begin{array}{l} \varphi :X\to Z\\ \phi :Z\to \hat{X} \end{array}$$ (1)

where *Z* represents the features of input data. The encoder network *ϕ* maps input data *X* to *Z*, and then the decoder network *φ* reconstructs *Z* to $\hat{X}$. The input data *X* is the XICs of fragments in a slider. The learning objective of autoencoder is to make $\hat{X}$ as close to *X* as possible.

The common encoder and decoder are designed as a stack of fully connected (FC) neural networks, which are simple with high computing speed. To achieve better performance on feature extraction task, we referenced the 4-branch networks idea of GoogLeNet [55] structure and constructed a 4-branch of FC VAE neural networks. In the networks, we set the number of neurons of FC layers to be equal to the channel size of inception block.

The network structure of encoder and decoder presents mirror symmetry (Fig. S1). The encoder network is a 4-branch network, and each branch consists of FC layers. The first branch network contains a FC 384-dimensional layer, followed by a dropout layer. The second branch network includes 2 FC layers with the dimensions of 192 and 384 and a dropout layer. The third branch network includes 2 FC layers with the dimensions of 48 and 128, and a dropout layer. The fourth branch only contains an FC 128-dimensional layer (Fig. S1).

The 20-dimensional input vector of encoder network is fragment XIC. The output vectors of the 4-branch networks are catenated by the appending operation at the end. The encoder network outputs two 16-dimensional vectors, one for the standard deviation (*σ*^2^) and the other for the mean value (*μ*). The mean vector represents the latent features of the input data. Then, $Z=\mu +\varepsilon *\sqrt{\sigma^{2}},\varepsilon \sim N(0,\;1)$ is fed to the decoder network, where *ε* is a random value sampled from Gaussian distribution (Fig. S1).

Then the 16-dimensional vector *Z* is fed to the decoder network consisting of 4-branch network. For the decoder network, the first branch network contains an FC 384-dimensional layer, followed by a dropout layer. The second branch network includes 2 FC layers with the dimensions of 384 and 192 and a dropout layer. The third branch network includes 2 FC layers with the dimensions of 128 and 48 and a dropout layer. The fourth branch only contains a FC 128-dimensional layer. The output vectors of the 4-branch networks are catenated by the appending operation at the end, and the size of catenation vector is 384 + 192 + 48 + 128 = 752. Then, the 752-dimensional catenation vector is fed to a 20-dimensional FC layer. Finally, the decoder network outputs a 20-dimensional vector as the reconstructed data of the input vector (Fig. S1).

To train the VAE, we input the anchor, positive, and negative XICs (*X_a_*, *X_p_*, *X_n_*) to the encoder network, respectively (Fig. 2A). The encoder network outputs the latent features (*μ_a_*, *μ_p_*, *μ_n_*) and the variance values ($\sigma_{a}^{2},\sigma_{p}^{2},\sigma_{n}^{2}$) corresponding to the anchor, positive, and negative XICs, respectively. Then, *Z_a_*, *Z_p_*, and *Z_n_* [$Z=\mu +\varepsilon *\sqrt{\sigma^{2}},\varepsilon \sim N\left(0,\;1\right)$] are fed to the decoder network, respectively. The decoder network reconstructs *Z_a_*, *Z_p_*, and *Z_n_* to $X_{a}^{\prime }$, $X_{p}^{\prime }$, and $X_{n}^{\prime }$, respectively. Here, we calculate the objective functions of classical VAE of anchor, positive, and negative XICs, respectively. The objective function of the classical VAE is defined by the following equations:

$$\begin{array}{l} {\text{Loss}}_{\mathrm{VAE}}=-{\text{Loss}}_{\mathrm{KL}}+{\text{loss}}_{\text{recon}}\\ {\text{Loss}}_{\mathrm{KL}}=-\frac{1}{2}\left(\log\sigma^{2}-\mu^{2}-\sigma^{2}+1\right)\\ {\text{Loss}}_{\text{recon}}=\frac{1}{N}\sum {\left\|X-X^{\prime }\right\|}^{2} \end{array}$$ (2)

The *Loss_VAE_* of anchor, positive, and negative XICs is defined to${\mathrm{Loss}}_{\mathrm{VAE}}^{a}$, ${\mathrm{Loss}}_{\mathrm{VAE}}^{p}$, and ${\mathrm{Loss}}_{\mathrm{VAE}}^{n}$.Then, we calculate the triplet loss using *μ_a_*, *μ_p_*, and *μ_n_*. The triplet loss is defined by the following equation:

$${\mathrm{Loss}}_{\mathrm{triplet}}=\frac{1}{N}\sum_{i=1}^{N}{\left[{\left\|\mu_{a}^{i}-\mu_{p}^{i}\right\|}^{2}-{\left\|\mu_{a}^{i}-\mu_{n}^{i}\right\|}^{2}+\alpha \right]}_{+}$$ (3)

where *α* is a margin parameter which is set to 1. In addition, ‖∗‖^2^ presents the square of Euclidean distance. Finally, we combine the VAE loss and the triplet loss as the final optimized function *Loss_total_*:

$${\mathrm{Loss}}_{\mathrm{total}}=\frac{1}{3}\left({\mathrm{Loss}}_{\mathrm{VAE}}^{a}+{\mathrm{Loss}}_{\mathrm{VAE}}^{p}+{\mathrm{Loss}}_{\mathrm{VAE}}^{n}\right)+{\mathrm{Loss}}_{\mathrm{triplet}}$$ (4)

In the above training process, the anchor, positive, and negative XICs are input to the encoder network, respectively. The encoder network outputs the latent features of each input fragment XIC, whether it is anchor, positive, or negative. Therefore, when making prediction, the input data of the trained neural network model are all fragment XICs in a slider, rather than a single labeled XIC.

The training data come from the results quantified by OpenSWATH. We use DIA-Umpire to analyze DIA file for generating pseudo-DDA spectra and then use OpenSWATH to quantify the peptides contained in spectral library. We apply the information (fragment *m*/*z* and retention time of peptides) of the OpenSWATH output file to extract the fragment XICs of quantified peptide from the DIA file. Afterward, we randomly choose 2 fragment XICs from the same peptide as anchor and positive data. The negative data are randomly selected from the different peptides. Finally, we combine the anchor, positive, and negative data as triple data.

We trained the Dear-DIA^XMBD^ on an *E. coli* SWATH dataset with 100 variable MS1 windows, which are acquired by TripleTOF 5600 mass spectrometer and TripleTOF 6600 mass spectrometer. The dataset from TripleTOF 5600 contains 7 runs with the MS recording time varying from 30 to 240 min. The dataset from TripleTOF 6600 consist of 6 runs with the MS recording time varying from 15 min to 10 h. We manually selected 97,980 *E. coli* peptide precursor ions quantified by OpenSWATH (Fig. S3B). Each precursor ion contains top 6 fragment ion XICs. Then, we randomly picked 2 fragment XICs of the same precursor ion as anchor and positive XICs, respectively, and randomly selected a fragment XIC from other precursor ion as negative XIC to generate a total of 2,179,590 groups of triplet data as the training dataset.

We employed 6 common deep learning optimizers (Adadelta, Adagrad, Adamax, Nadam, SGD, and Adam) to optimize our model. By comparing the loss function curves of different optimizers, we decided to use Adam (adaptive moment estimation) to optimize our model to find more peptides. The update rules of Adam optimizer are defined by the following formula:

$$\begin{array}{c} g_{t}=\nabla_{\theta }f_{t}\left(\theta_{t-1}\right)\\ m_{t}=\beta_{1}\cdot m_{t-1}+\left(1-\beta_{1}\right)\cdot g_{t}\\ v_{t}=\beta_{2}\cdot v_{t-1}+\left(1-\beta_{2}\right)\cdot g_{t}^{2}\\ {\hat{m}}_{t}=\frac{m_{t}}{1-\beta_{1}^{t}}\\ {\hat{v}}_{t}=\frac{v_{t}}{1-\beta_{2}^{t}}\\ \theta_{t}=\theta_{t-1}-\eta \cdot \frac{{\hat{m}}_{t}}{\sqrt{{\hat{v}}_{t}}+\varepsilon } \end{array}$$ (5)

where *f_t_*(*θ*_*t* − 1_) is the loss function, *g_t_* is the gradient of the parameter *θ*, and *η* is the learning rate. The default value of *η* is 0.001. *β*_1_ and *β*_2_ are the parameters in the algorithm, generally *β*_1_ = 0.9 and *β*_2_ = 0.999. *m_t_* and *v_t_* are the first-order and the second-order moment estimation of the gradient, respectively. ${\hat{m}}_{t}$ and ${\hat{v}}_{t}$ are corrections to *m_t_* and *v_t_*, respectively, which can be approximated as an unbiased estimate of the expectation. When *ε* = 10^−8^, the zero denominator can be avoided.

### Triplet dataset generation

When the triplet loss is introduced into the model, we need to generate the triplet data to train neural network. The training data come from the results quantified by OpenSWATH (Fig. S3B). We stored XICs of the first 6 high-intense fragments of quantified peptides. Then, we randomly chose 2 fragment XICs from the same peptide as anchor and positive data. The negative data were randomly selected from the different peptides. Finally, we combined the anchor, positive, and negative data into triple data.

### Architecture and training process of CNN classifier

We applied CNN classifier to calculate the similarity of hit fragments that matched in silico peptide. Since the first 6 fragment ions were usually selected to observe their similarity during manual check, we fed the XICs of first 6 ions in hit fragments into CNN classifier. We used 1 and 0 to label fragment ions belonging to the same peptide and different peptide, respectively.

The length of each fragment XIC is 20, so that the input matrix of CNN contains 6 rows and 20 columns (Fig. S2). The CNN classifier consists of 4-branch (1-2-2-1) convolutional layers, which is the same with Inception block of GoogLeNet. The output feature maps of the 4-branch networks are catenated in channel dimension. The result of catenation is flattened into a vector, which is fed to a 512-dimensional FC layers. The last layer reports the similarity score, which locates between 0 to 1 (Fig. S2). The loss function of CNN classifier is the binary cross entropy (BCE) function, which is defined by the following equation:

$${\mathrm{Loss}}_{\mathrm{BCE}}=-\frac{1}{N}\sum_{i=1}^{N}\left[t_{i}\cdot \log\left(y_{i}\right)+\left(1-t_{i}\right)\cdot \left(1-y_{i}\right)\right]$$ (6)

where *t_i_* and *y_i_* represent the label of input data and the output of CNN classifier, respectively. *N* is the number of input matrixes. We used Adam optimizer with default parameters to train CNN classifier and treated the outputs of CNN classifier as the similarity scores of input fragment groups.

### PIndex querying algorithm

To match fragments with precursors, we developed PIndex querying algorithm based on the inverted index algorithm. PIndex starts with in silico digestion of protein FASTA database and then generates the in silico digested peptide information sets which contain the charge of precursor, the *m*/*z* of precursor, and the *m*/*z* list of fragments. We allocated the unique index to each information set. Obviously, we can query the in silico precursors and fragments using the peptide indexes.

Next, we created the inverted index table between the peptide indexes and the in silico digested peptides. The inverted querying process includes 2 parts: One is to map precursors to peptide indexes, and the other is map fragments to peptide indexes. Precursor query maps the precursor identifiers, including *m*/*z* and charge, to peptide index set, which is named Index1. Fragment query maps the fragment *m*/*z* to peptide index set, which is named Index2. We calculated the intersection of Index1 and Index2, and then we can obtain the peptides that were hit by both fragments and precursors. Querying the same peptide index indicates that the precursor and fragments come from the same peptide.

### Two-stage clustering method

In the first *k*-means clustering, we obtained numerous fragment ion combinations. These combinations were matched with precursors using PIndex querying. However, affected by the interference signal, there are still some fragment ions in the clustering results that did not match the precursors. Therefore, we removed the precursor–fragment pairs in the first clustering, and used *k*-means to cluster the remaining fragment ions again to improve the usage of fragment ions.

### MS1 recalibration

When the MS is not calibrated for long, the masses will often exhibit systematic shifts. The proper calibration can improve identification, alignment, and quantification. We referenced mzRecal [40], a universal MS1 recalibration method using high confidence peptides as internal calibrants, to improve the performance of Dear-DIA^XMBD^. We selected the peptides with X!Tandem expected values less than 0.001 as potential calibrants, and then use the following mzRecal formula to calibrate the MS1 *m*/*z*: Orbitrap instrument: $m^{\prime }=\frac{A}{{\left(\sqrt{m}-B\right)}^{2}}$, time-of-flight instrument: $m^{\prime }=\mathrm{Am}+B\sqrt{m}+C$, where *m*^′^ is the calibrated *m*/*z* and *m* is the experimental *m*/*z*. Parameters A, B, and C can be calculated by curve fitting method.

## Acknowledgments

We would like to express our deep appreciation to D. Wang, S. X. Shuai, P. Shaw, H. Shaw, and X. Liu for the helpful suggestions while drafting the manuscript. We thank Z. Xu and Y. Yu for help with the high-performance computer. **Funding:** This project is supported by the Ministry of Science and Technology of the People's Republic of China (STI2030-Major Projects 2021ZD0201900 to J.S.), the National Natural Science Foundation of China (grant nos. 12090052 to J.S., 81788101 to J.H., 11704318 to X.Li., and J1310027 to C.-Q.Z.), and the Fundamental Research Funds for the Central Universities (grant nos. 20720230017 to X.Li, and 20720190087 to C.-Q.Z.). **Author contributions:** J.S., Q.H., and C.-Q.Z. conceived the project. Q.H. developed the algorithm, implemented the software, and wrote the manuscript. C.-Q.Z. acquired MS data for training the deep neural network. X.Li analyzed the data and results. H.G. plotted figures for the Supplementary Materials. X. Liu, F.Z., and T.G. analyzed data with Spectronaut software. J.S., Y.L., M.G., R.Y., D.G., and F.Y. discussed the algorithms. J.S. and J.H. wrote the manuscript and supervised the project. **Competing interests:** The authors declare that they have no competing interests.

## Data Availability

The new raw MS data of L929 mouse samples, *E. coli* training data (TripleTOF 5600), and the analysis results have been deposited to the ProteomeXchange Consortium [56] (http://proteomecentral.proteomexchange.org) via the iProX [57] partner repository with the iProX identifier IPX0003690000 and the PRIDE identifier PXD029694. The login URL and password of the IPX0003690000 are “https://www.iprox.cn/page/SSV024.html;url=1684680054320VxOd” and “Password: jXrU”. The public raw MS data of *E. coli* (TripleTOF 6600), HYE124-mixed samples, BGS mouse, and TNFR1 complex dataset are available with the dataset identifier PXD020761, PXD002952, PXD011691, and PXD002177. The SGS datasets are available from the PeptideAtlas raw data repository with accession number PASS00289.

## Supplementary Materials

Supplementary 1 Figure S1. The structure of VAE neural network.
Figure S2. The structure of CNN classifier.
Figure S3. The schematic diagram of Dear-DIA^XMBD^ and OpenSWATH analysis workflow.
Figure S4. Venn diagrams of identified peptides and proteins found from SGS human and mouse L929 datasets.
Figure S5. The XICs of synthesized peptides SGS_80-FSQAGSEVSALLGR identified in SGS human dataset by Dear-DIA^XMBD^ but not identified by DIA-Umpire.
Figure S6. The XICs of synthesized peptides identified in SGS human dataset by Dear-DIA^XMBD^ but not identified by DIA-Umpire.
Figure S7. The log_2_-scaled distributions of peptide and protein intensities discovered from SGS human dataset.
Figure S8. Venn diagrams of peptides and proteins found from SGS human dataset.
Figure S9. Venn diagrams of peptides and proteins found from L929 mouse dataset.
Figure S10. The log_2_-scaled distributions of peptide and protein intensities discovered from mouse L929 mouse dataset.
Figure S11. Venn diagrams of peptides and proteins found from HYE124 TOF6600 64var dataset.
Figure S12. Distribution of the number of peptides from HYE124 TOF6600 64var dataset.
Figure S13. The number of peptides with coefficient of variation below 20% from HYE124 TTOF6600 64var dataset (sample A with sample B).
Figure S14. The XICs of peptides identified in HYE124 TTOF6600 64var dataset by Dear-DIA^XMBD^ but not identified by Spectronaut14 and DIA-Umpire.
Figure S15. LFQbench test performance of DIA-Umpire for HYE124 Triple TOF 6600 64var dataset.
Figure S16. Venn diagrams of peptides and proteins found from HYE124 Triple TOF 5600 64var dataset.
Figure S17. Distribution of the number of peptides from HYE124 TOF5600 64var dataset.
Figure S18. Venn diagrams of peptides and proteins found from HYE124 Triple TOF 5600 64var dataset.
Figure S19. The number of peptides with coefficient of variation below 20% from HYE124 TTOF5600 64var dataset (sample A with sample B).
Figure S20. LFQbench test performance of HYE124 Triple TOF 5600 64var dataset.
Figure S21.Venn diagrams of peptides and proteins found from BGS mouse DIA dataset.
Figure S22.The heatmaps of protein intensities of Dear-DIA^XMBD^ and manual analysis in TNFR1 dataset.
Figure S23. The parameters of Philosopher (v4.8.1).
Figure S24.The parameters of DIA-NN (v1.8.1).
Table S1. The LFQbench metrics of peptides and proteins found by Dear-DIA^XMBD^, Spectronaut 14, and DIA-Umpire of HYE124 64-var dataset (TripleTOF 6600).
Table S2. The parameters of MSFragger search engines.
Table S3. The parameters of Dear-DIA^XMBD^.
Table S4. The parameters of Spectronaut 14.
Table S5. The parameters of DIA-Umpire.
Text S1. The times required for training and running Dear-DIA^XMBD^, DIA-Umpire, and Spectronaut 14.
Text S2. The comparison between VAE and a simplistic precursor–fragment grouping algorithm.
Text S3. The comparison results between Dear-DIA^XMBD^ with PIndex and Dear-DIA^XMBD^ without PIndex.
Text S4. The results of simplistic training VAE using Orbitrap data and QTOF data.
Text S5. The limitations of Pearson correlation coefficient.
Text S6. A comparison between the Gaussian curve fitting model and the VAE model.
Text S7. The FDR validation of Dear-DIA^XMBD^, DIA-Umpire, and Spectronaut 14.
Text S8. The installation instruction of Dear-DIA^XMBD^.
