# Supplementary material for: Dear-DIAXMBD: Deep Autoencoder Enables Deconvolution of Data-Independent Acquisition Proteomics
Source: Research (Wash D C). 2023 Jun 26;6:0179. doi: 10.34133/research.0179 (PMC10292580; doi:10.34133/research.0179)
Supplement: Supplementary 1 — Figure S1. The structure of VAE neural network. Figure S2. The structure of CNN classifier. Figure S3. The schematic diagram of Dear-DIAXMBD and OpenSWATH analysis workflow. Figure S4. Venn diagrams of identified peptides and proteins found from SGS human and mouse L929 datasets. Figure S5. The XICs of synthesized peptides SGS_80-FSQAGSEVSALLGR identified in SGS human dataset by Dear-DIAXMBD but not identified by DIA-Umpire. Figure S6. The XICs of synthesized peptides identified in SGS human dataset by Dear-DIAXMBD but not identified by DIA-Umpire. Figure S7. The log2-scaled distributions of peptide and protein intensities discovered from SGS human dataset. Figure S8. Venn diagrams of peptides and proteins found from SGS human dataset. Figure S9. Venn diagrams of peptides and proteins found from L929 mouse dataset. Figure S10. The log2-scaled distributions of peptide and protein intensities discovered from mouse L929 mouse dataset. Figure S11. Venn diagrams of peptides and proteins found from HYE124 TOF6600 64var dataset. Figure S12. Distribution of the number of peptides from HYE124 TOF6600 64var dataset. Figure S13. The number of peptides with coefficient of variation below 20% from HYE124 TTOF6600 64var dataset (sample A with sample B). Figure S14. The XICs of peptides identified in HYE124 TTOF6600 64var dataset by Dear-DIAXMBD but not identified by Spectronaut14 and DIA-Umpire. Figure S15. LFQbench test performance of DIA-Umpire for HYE124 Triple TOF 6600 64var dataset. Figure S16. Venn diagrams of peptides and proteins found from HYE124 Triple TOF 5600 64var dataset. Figure S17. Distribution of the number of peptides from HYE124 TOF5600 64var dataset. Figure S18. Venn diagrams of peptides and proteins found from HYE124 Triple TOF 5600 64var dataset. Figure S19. The number of peptides with coefficient of variation below 20% from HYE124 TTOF5600 64var dataset (sample A with sample B). Figure S20. LFQbench test performance of HYE124 Triple TOF 5600 64var dataset. [file research.0179.f1.docx]

Supplementary Materials


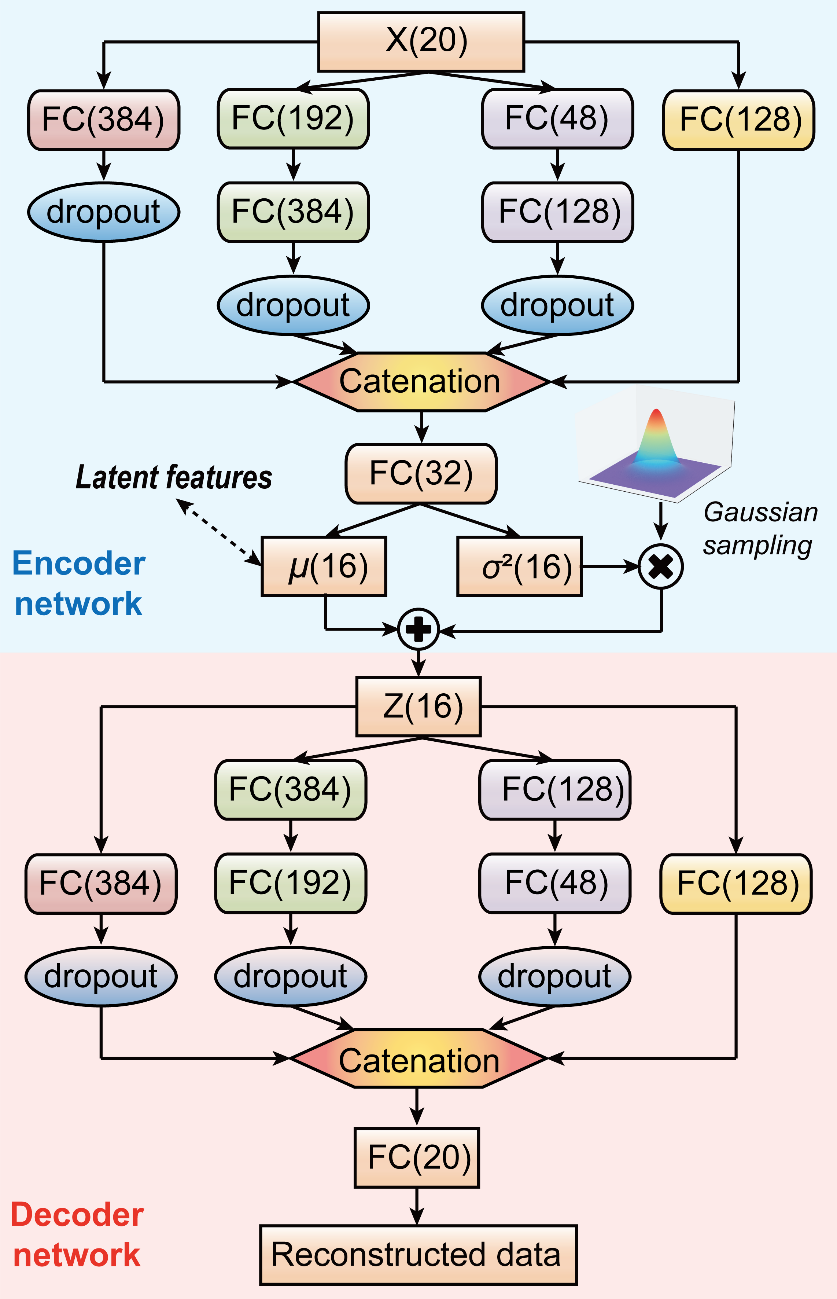


**Figure S1. The structure of VAE neural network.** The blue and red areas indicate the encoder neural network and the decoder neural network, respectively. The network structures of encoder and decoder present mirror symmetry. The 20-dimensional input vector X is fed to the four-branch encoder network which is consisted of 1-2-2-1 fully connected (FC) layers. The number in brackets indicates the dimension of the fully connected layer. The output vectors of the four-branch networks are catenated by the appending operation at the end. The encoder network outputs two 16-dimenasinal vectors, one for the standard deviation (σ^2^) and one for the mean value (μ). The mean vector represents the latent features of the input data. Then, Z=μ+ε*sqrt(σ^2^) (ε~N(0,1)) are fed to the decoder network, where ε is a random value sampling from Gaussian distribution. The 16-dimensinal vector Z is fed to the decoder network consisted of four-branch network. The decoder network outputs a 20-dimensional vector as the reconstructed data of input vector.


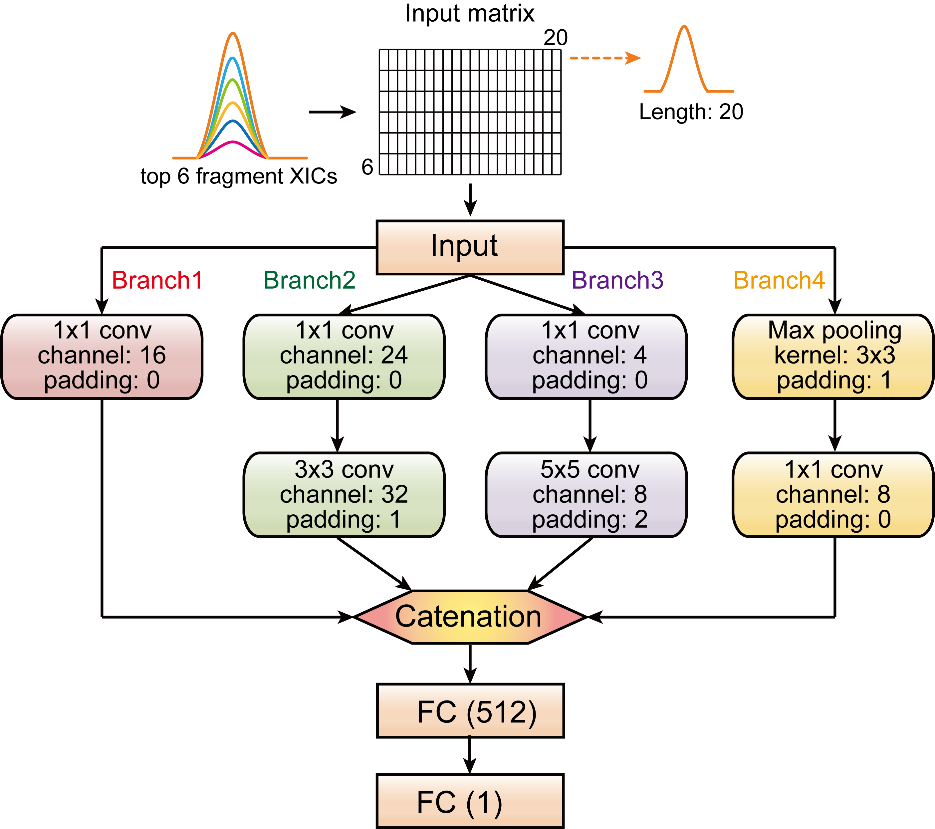


**Figure S2. The structure of convolutional neural network (CNN) classifier.** The input matrix contains 6 rows and 20 columns, which consists of top 6 fragment XICs of peptide precursor quantified by OpenSWATH. Each row represents a fragment XIC with a length of 20. The input matrix is fed to the four-branch network which is consisted of 1-2-2-1 convolutional layers. Branch1 is a single convolutional layer with the kernel size of (1, 1), the channel size of 16, the padding size of 0, and ReLU activation. Branch2 contains two convolutional layers with the kernel size of (1, 1) and (3, 3), the channel size of 16 and 32, the padding size of 0 and 1, respectively, and ReLU activation. Branch3 consists of two convolutional layers with the kernel size of (1, 1) and (5, 5), the channel size of 4 and 8, the padding size of 0 and 2, respectively, and ReLU activation. Branch4 includes a max pooling layer with the kernel size of (3, 3) and the padding size of 1 and a convolutional layer with the kernel size of (1, 1), the channel size of 8 and the padding size of 0. The output feature maps of the four-branch networks are catenated at channel dimension. The result of catenation is flattened into a vector, which is fed to a fully connected (FC) layers with 512 dimensions. The number in brackets indicates the dimension of the fully connected layer. The last layer reported similarity score, which located between 0 to 1.


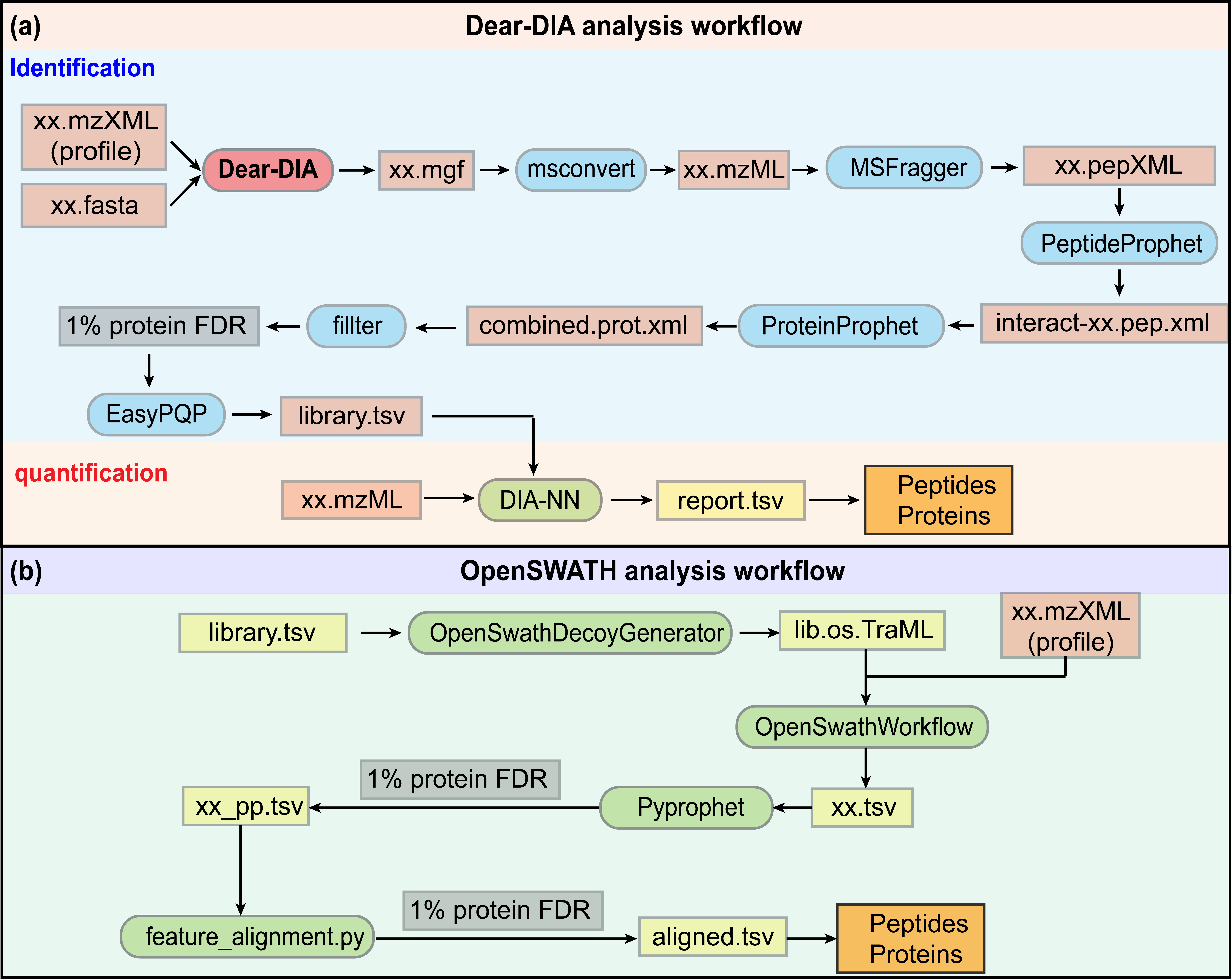


**Figure S3. The schematic diagram of Dear-DIA^XMBD^ and OpenSWATH analysis workflow. (a)** Dear-DIA^XMBD^ workflow consists of identification and quantification. The blue region and orange region indicates the identified and quantified processes, respectively. The frames with light red and light yellow colors indicate the I/O files of identification and quantification, respectively. The frames with light blue and light green colors represent the software tools using in identification and quantification, respectively. **(b)** OpenSWATH analysis workflow. The yellow and green colors indicate the I/O files and software, respectively.


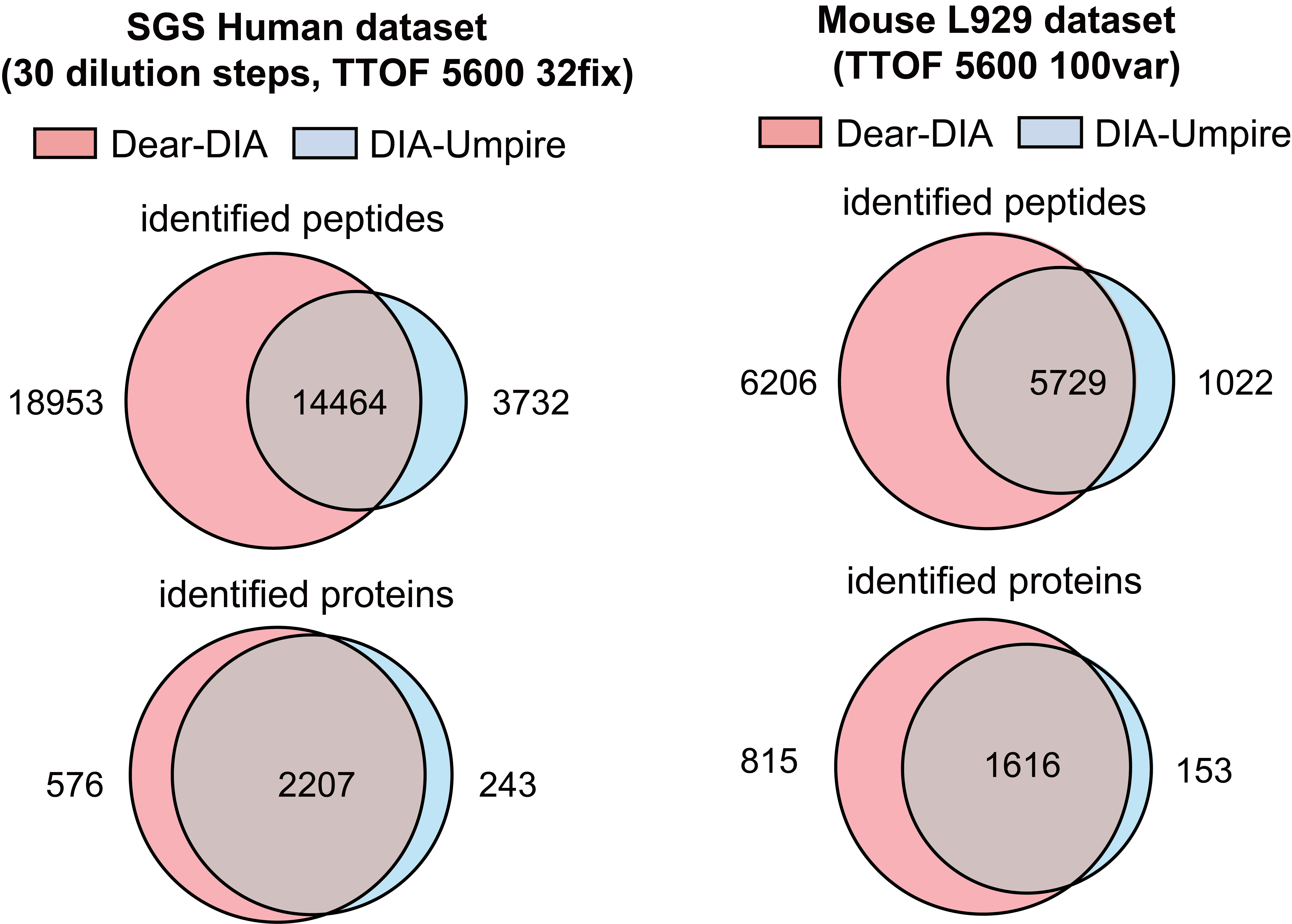


**Figure S4. Venn diagrams of identified peptides and proteins found from SGS Human and Mouse L929 datasets.** The peptides and proteins in spectral library of SGS Human and Mouse L929 datasets. The red circles and blue circles represent the results of Dear-DIA^XMBD^ and DIA-Umpire.


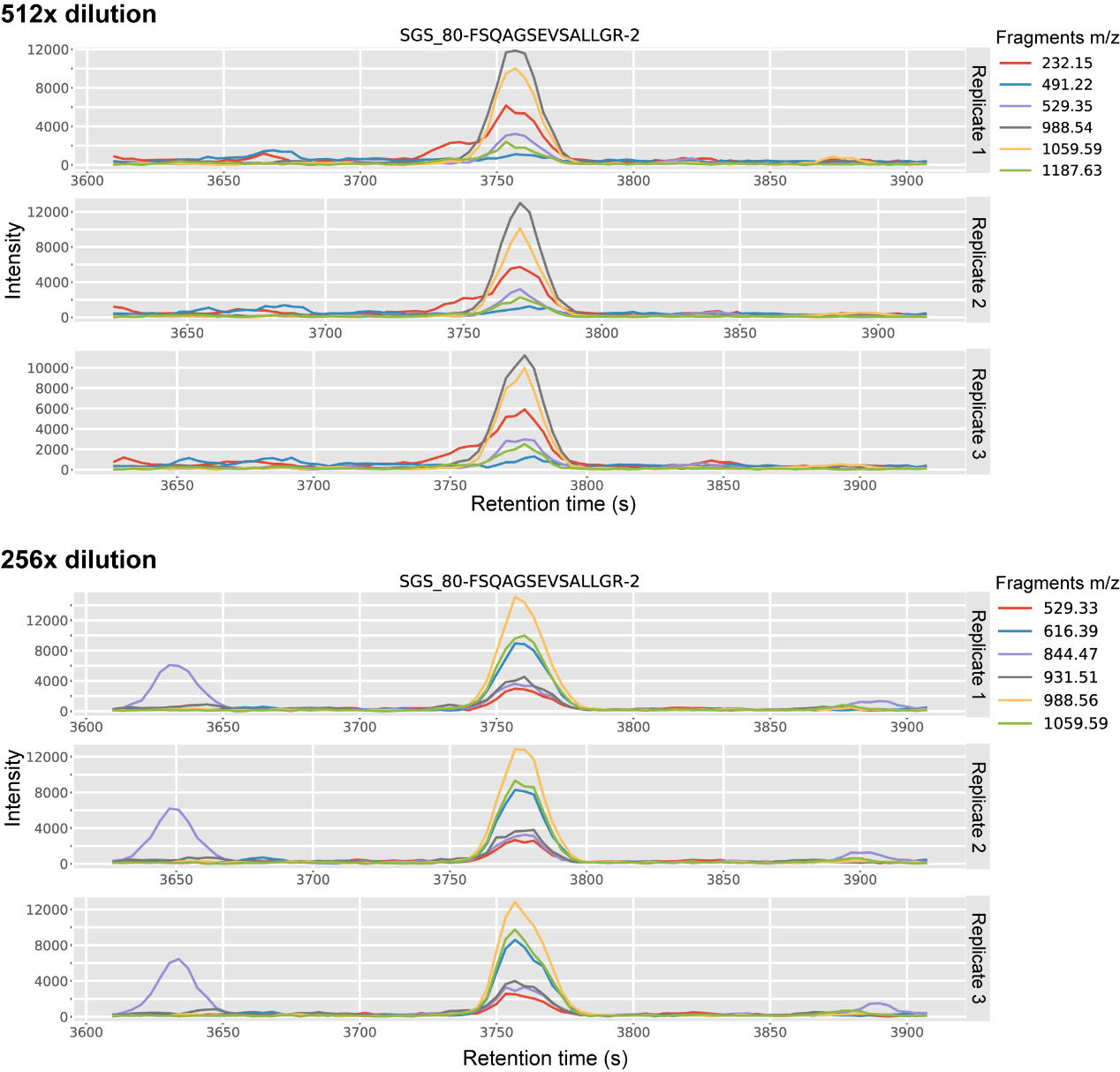


**Figure S5. The XICs of synthesized peptides SGS_80-FSQAGSEVSALLGR identified in SGS human dataset by Dear-DIA^XMBD^ but not identified by DIA-Umpire.** The colored lines are the XICs of fragments. X-axis is the retention time. Y-axis is the intensity of XICs of fragments. The 512X and 256X at the left top side of the figure represent the dilution times for the synthesized peptide.


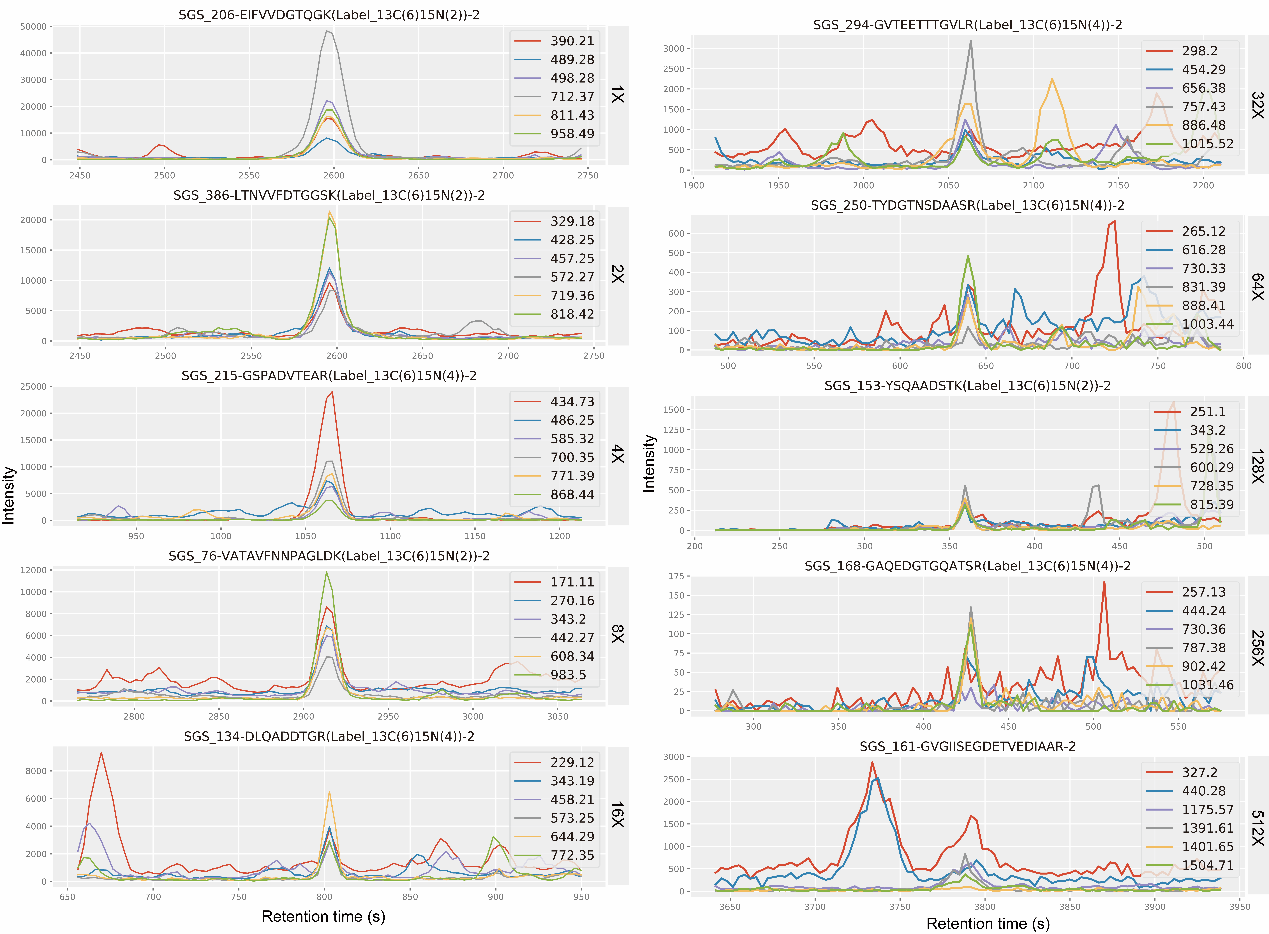


**Figure S6. The XICs of synthesized peptides identified in SGS human dataset by Dear-DIA^XMBD^ but not identified by DIA-Umpire.** The colored lines are the XICs of fragments. X-axis is the retention time. Y-axis is the intensity of XICs of fragments. The 1X, 2X, 4X, 8X, 16X, 32X, 64X, 128X, 256X, and 512X at the right side of the figure represent the dilution times for the synthesized peptides. The legend of each subplot represents the m/z of the corresponding fragments.


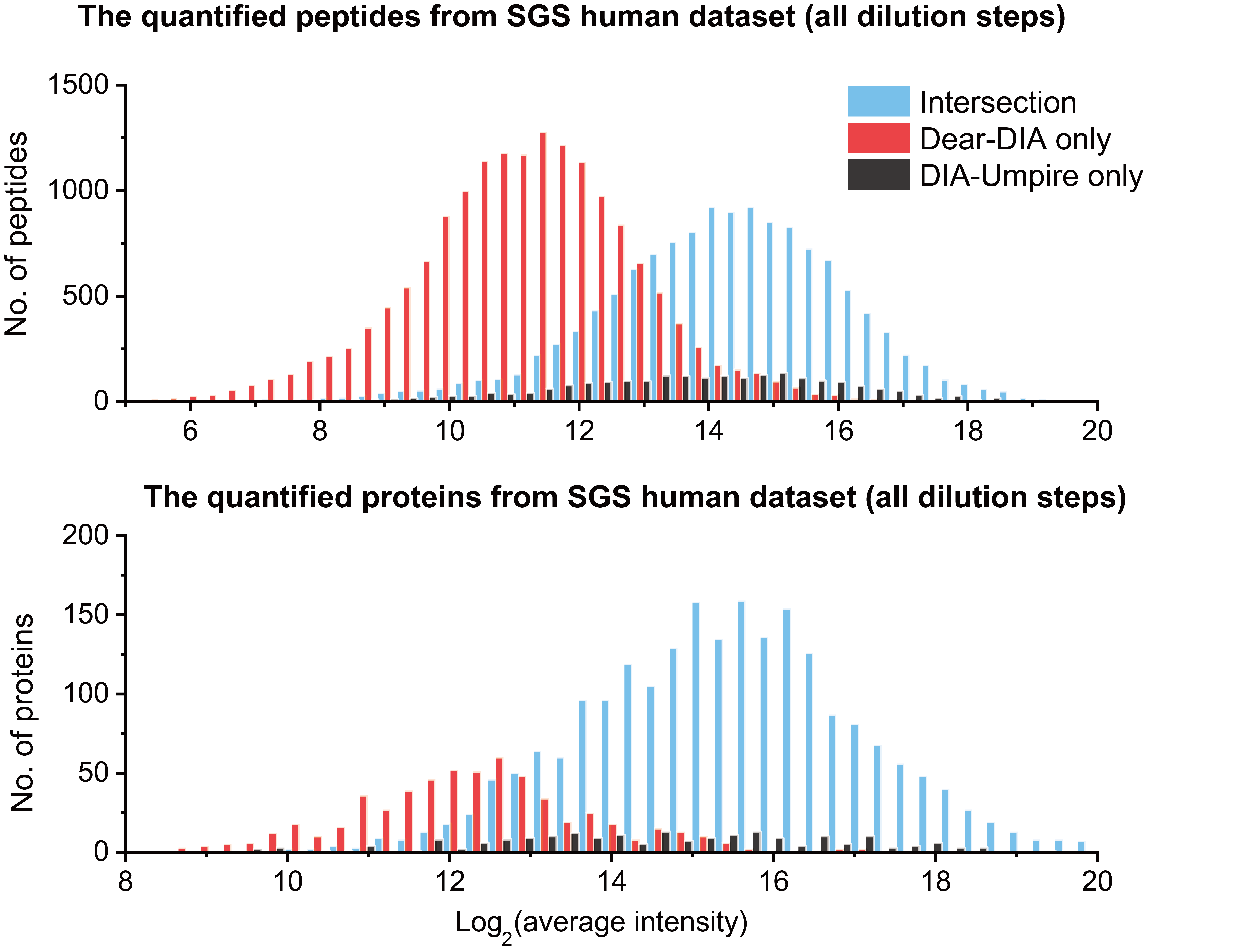


**Figure S7. The log2-scaled distributions of peptide and protein intensities discovered from SGS human dataset.** The peptides and proteins are quantified by DIA-NN. The peptides and proteins shared jointly with DIA-Umpire and Dear-DIA^XMBD^ are shown in light blue; the peptides and proteins reported exclusively by Dear-DIA^XMBD^ and DIA-Umpire are shown in red and black, respectively.


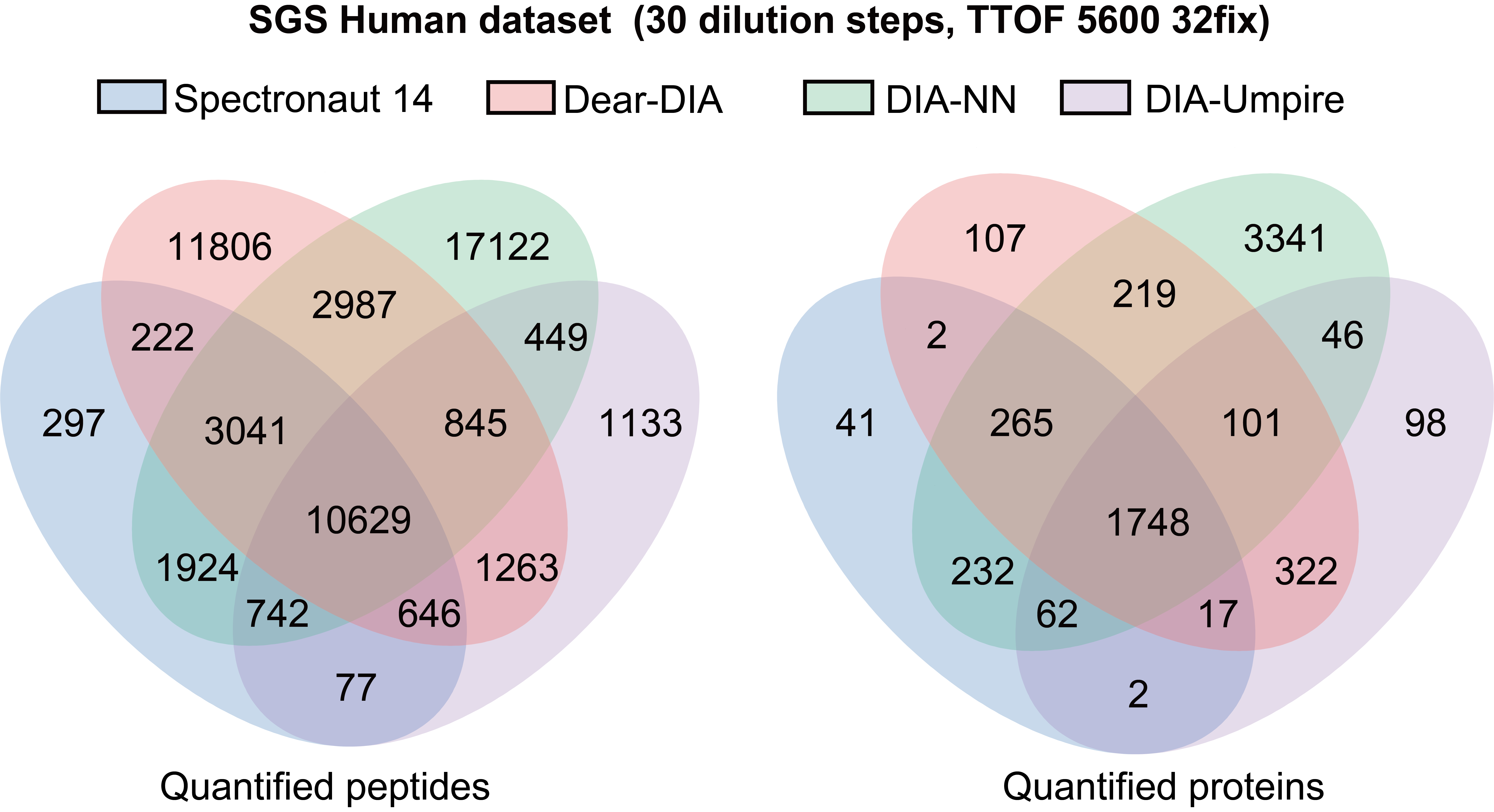


**Figure S8. Venn diagrams of peptides and proteins found from SGS human dataset.** The comparison of the numbers of quantified peptides and proteins obtained by Dear-DIA^XMBD^, DIA-Umpire, DIA-NN, and Spectronaut 14 from SGS human dataset with 30 dilution steps. The blue circles, red circles, green circles, and purple circles represent the results of Spectronaut 14, Dear-DIA^XMBD^, DIA-NN, and DIA-Umpire, respectively.


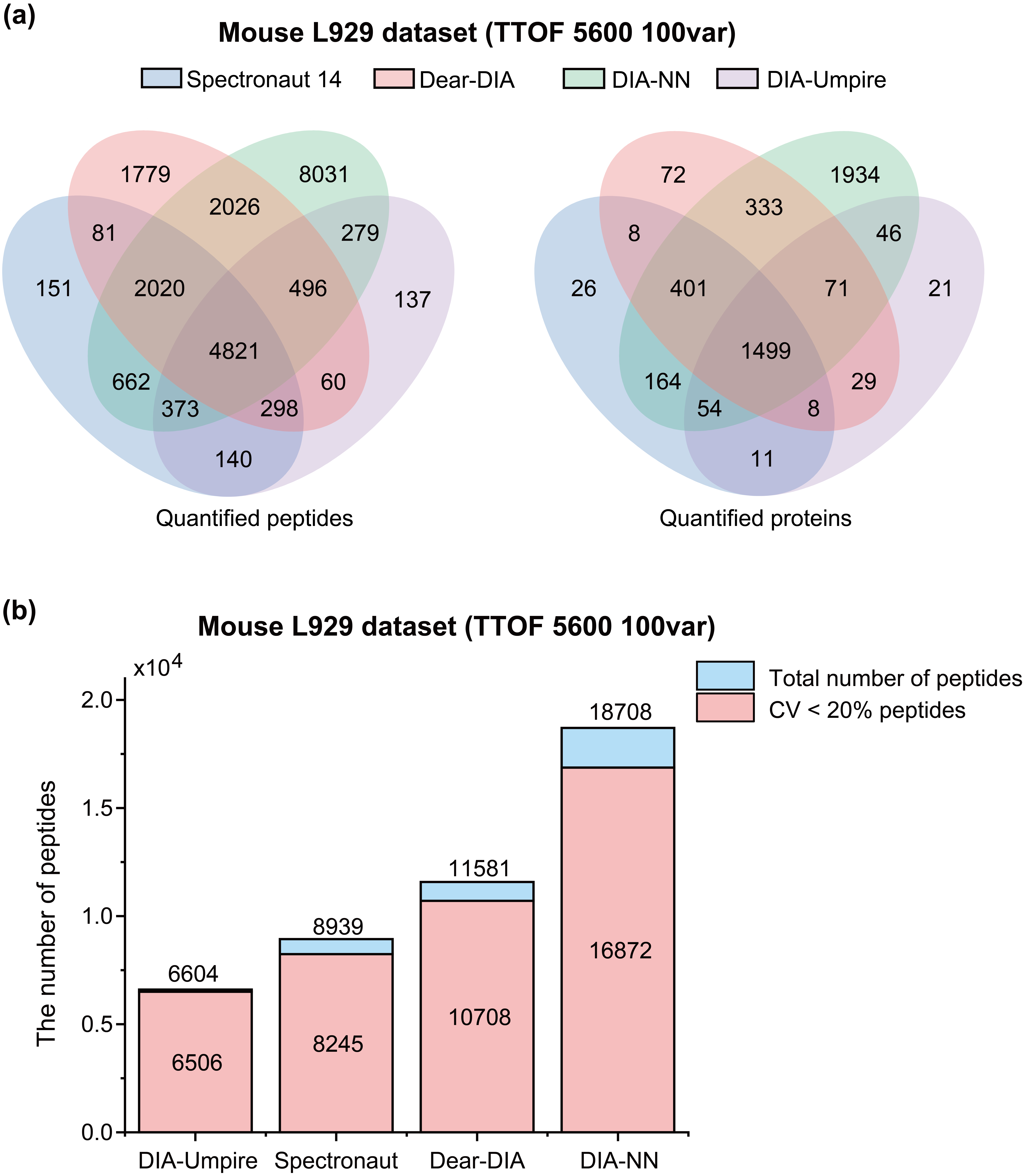


**Figure S9. (a)Venn diagrams of peptides and proteins found from L929 mouse dataset.** The comparison of the numbers of quantified peptides and proteins obtained by Dear-DIA^XMBD^, DIA-Umpire, DIA-NN, and Spectronaut 14 from L929 mouse dataset. The blue circles, red circles, green circles, and purple circles represent the results of Spectronaut 14, Dear-DIA^XMBD^, DIA-NN, and DIA-Umpire, respectively. **(b) The number of peptides with coefficient of variation (CV) below 20%.** The red and blue part represent the number of peptides with CV below 20% and the total number of peptides, respectively.


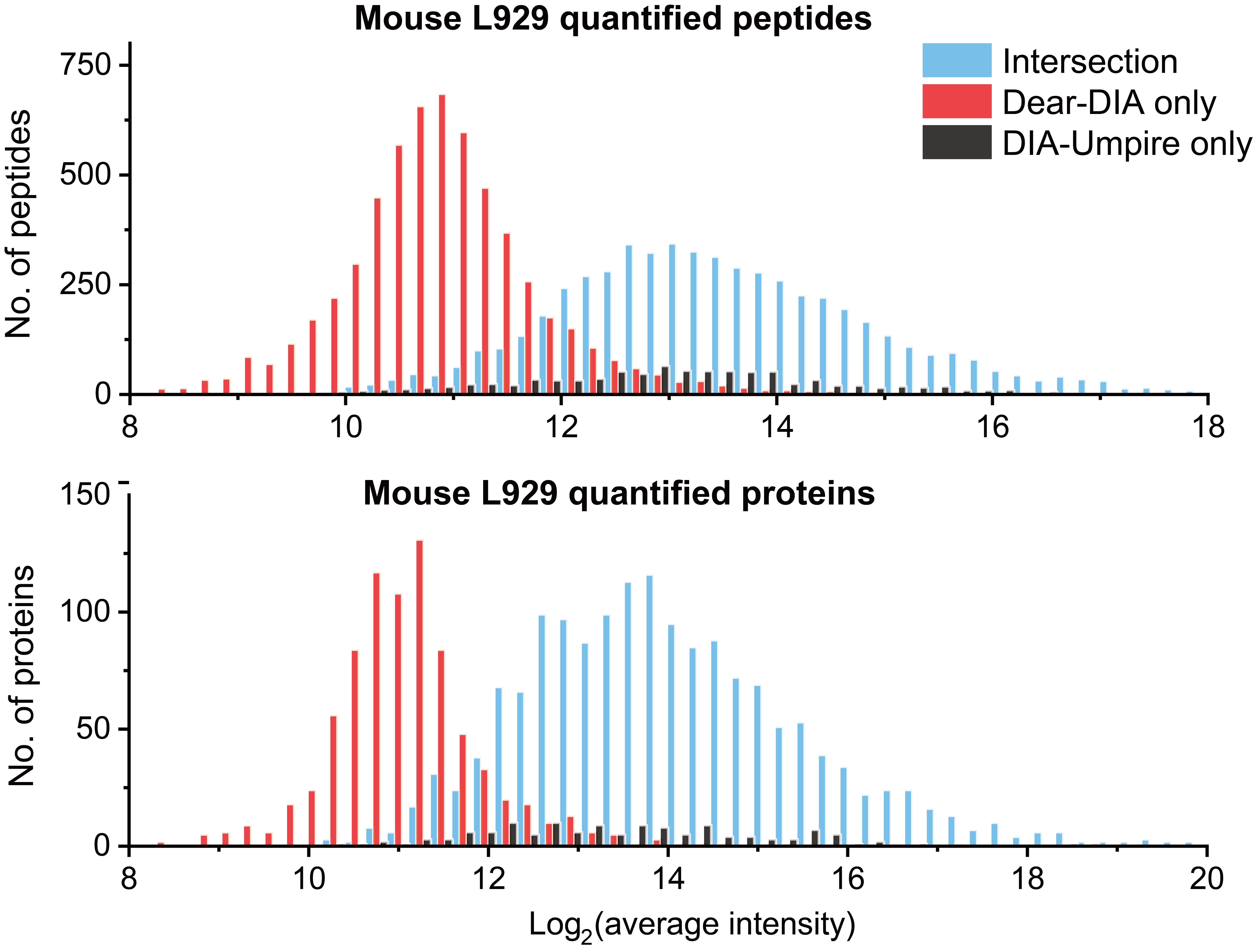


**Figure S10. The log2-scaled distributions of peptide and protein intensities discovered from mouse L929 mouse dataset.** The peptides and proteins are quantified by DIA-NN. The peptides and proteins shared jointly with DIA-Umpire and Dear-DIA^XMBD^ are shown in light blue; the peptides and proteins reported exclusively by Dear-DIA^XMBD^ and DIA-Umpire are shown in red and black, respectively.


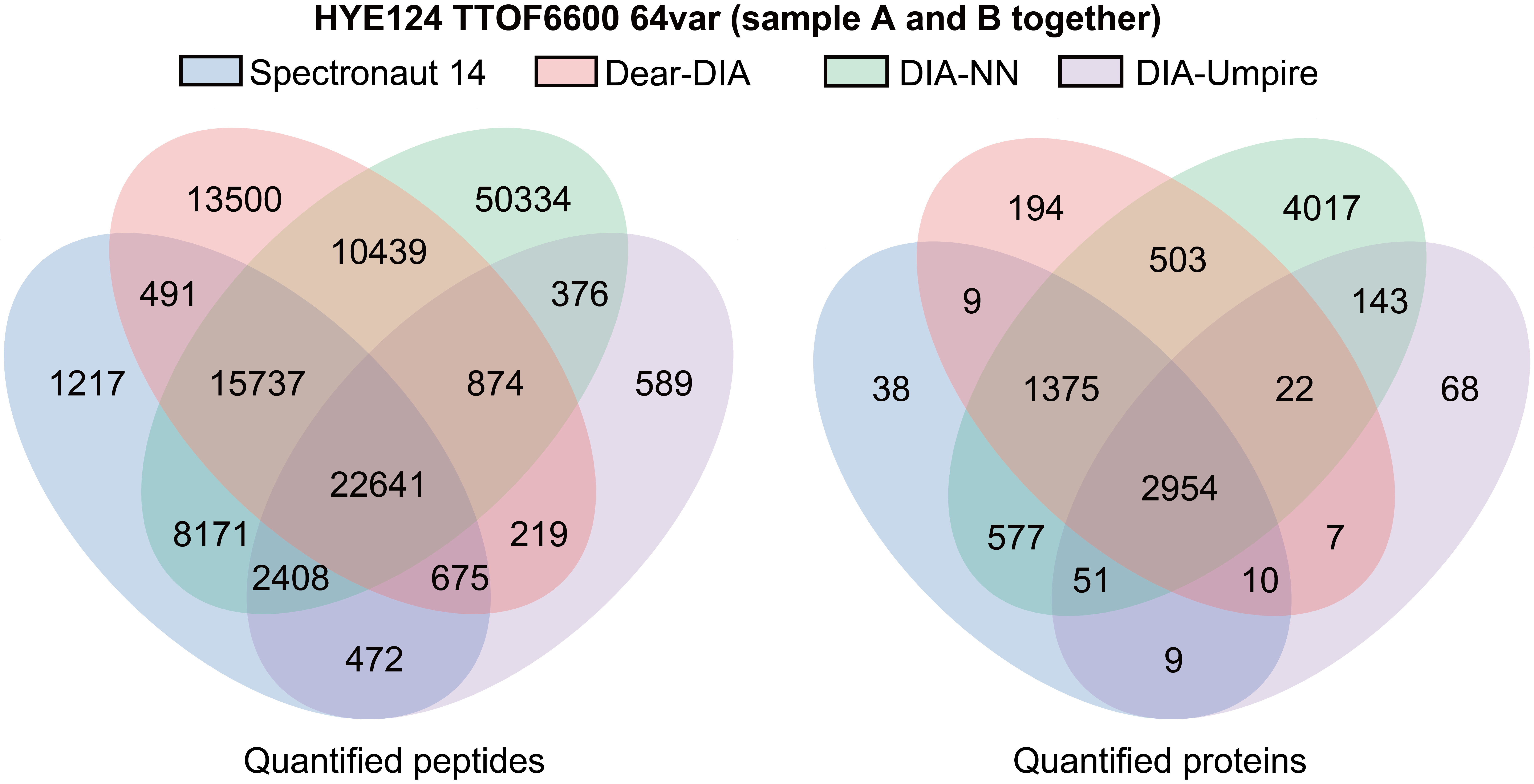


**Figure S11. Venn diagrams of peptides and proteins found from HYE124 TOF6600 64var dataset.** The comparison of the numbers of quantified peptides and proteins obtained by Dear-DIA^XMBD^, DIA-Umpire, DIA-NN, and Spectronaut 14 from HYE124 TOF6600 64var dataset. The blue circles, red circles, green circles, and purple circles represent the results of Spectronaut 14, Dear-DIA^XMBD^, DIA-NN, and DIA-Umpire, respectively.


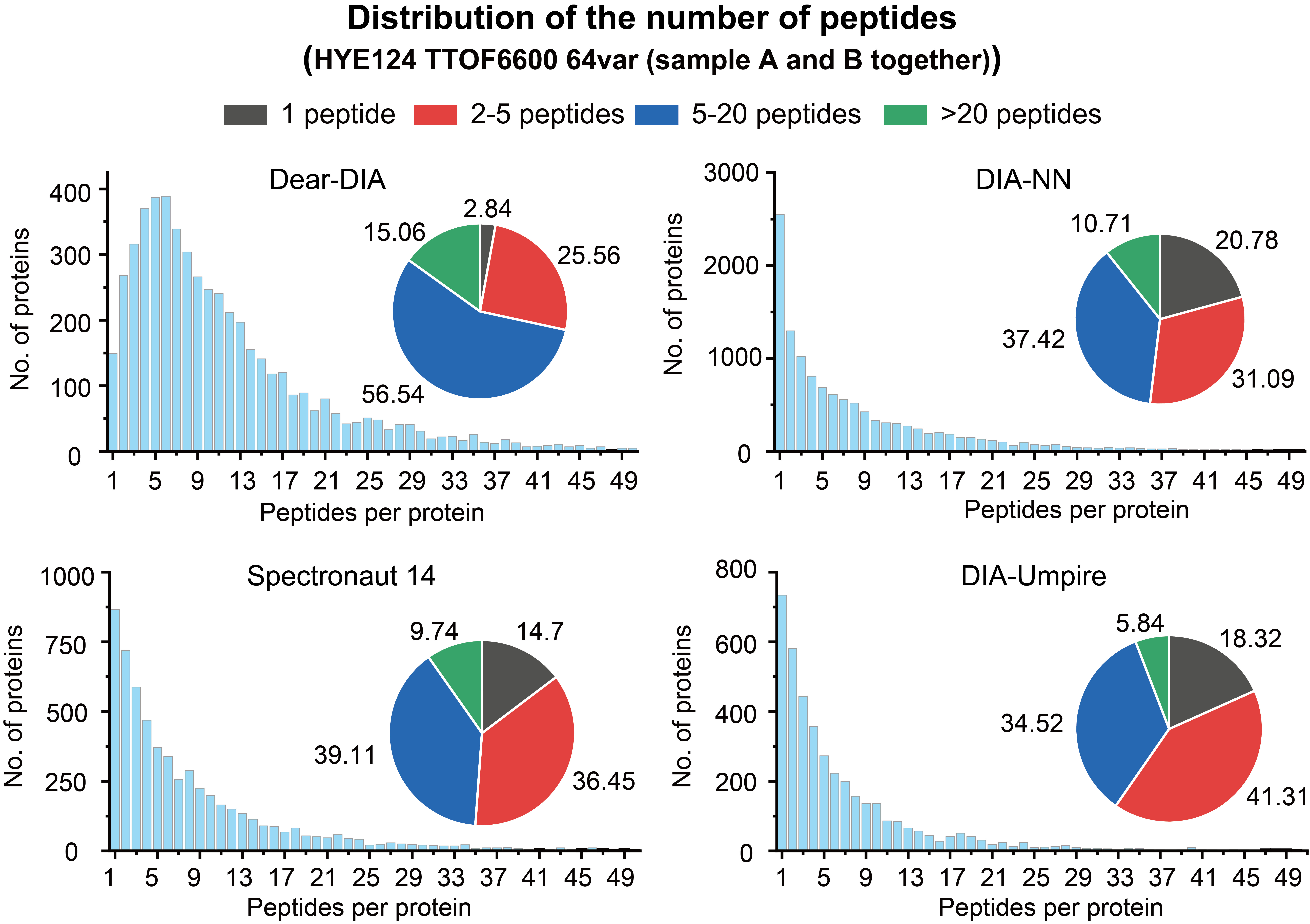


**Figure S12. Distribution of the number of peptides from HYE124 TOF6600 64var dataset.** The x-axis and y-axis represent the number of peptides and the number of proteins, respectively.


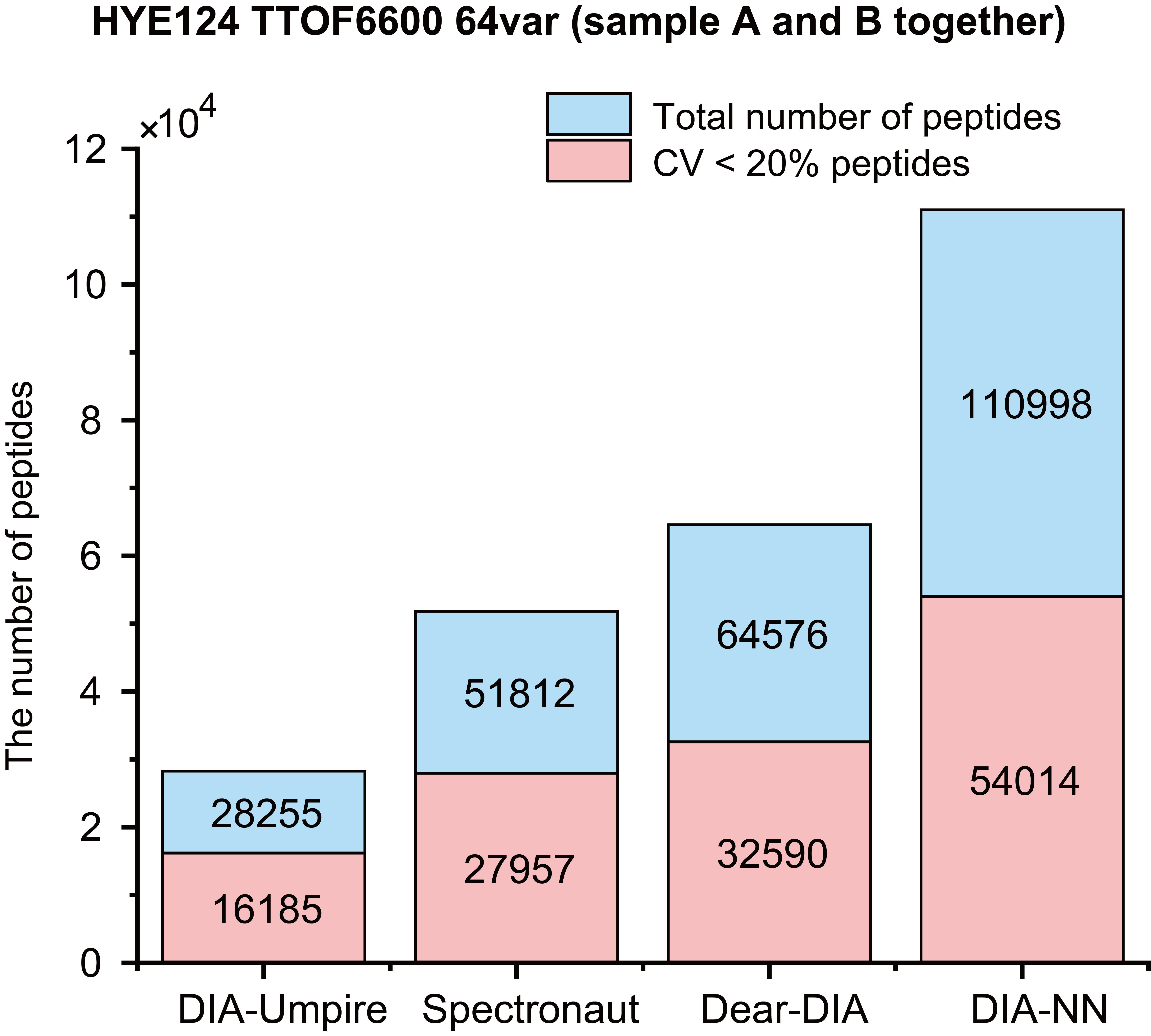


**Figure S13. The number of peptides with coefficient of variation (CV) below 20% from HYE124 TTOF6600 64var dataset (sample A with sample B).** The red and blue part represent the number of peptides with CV below 20% and the total number of peptides, respectively.


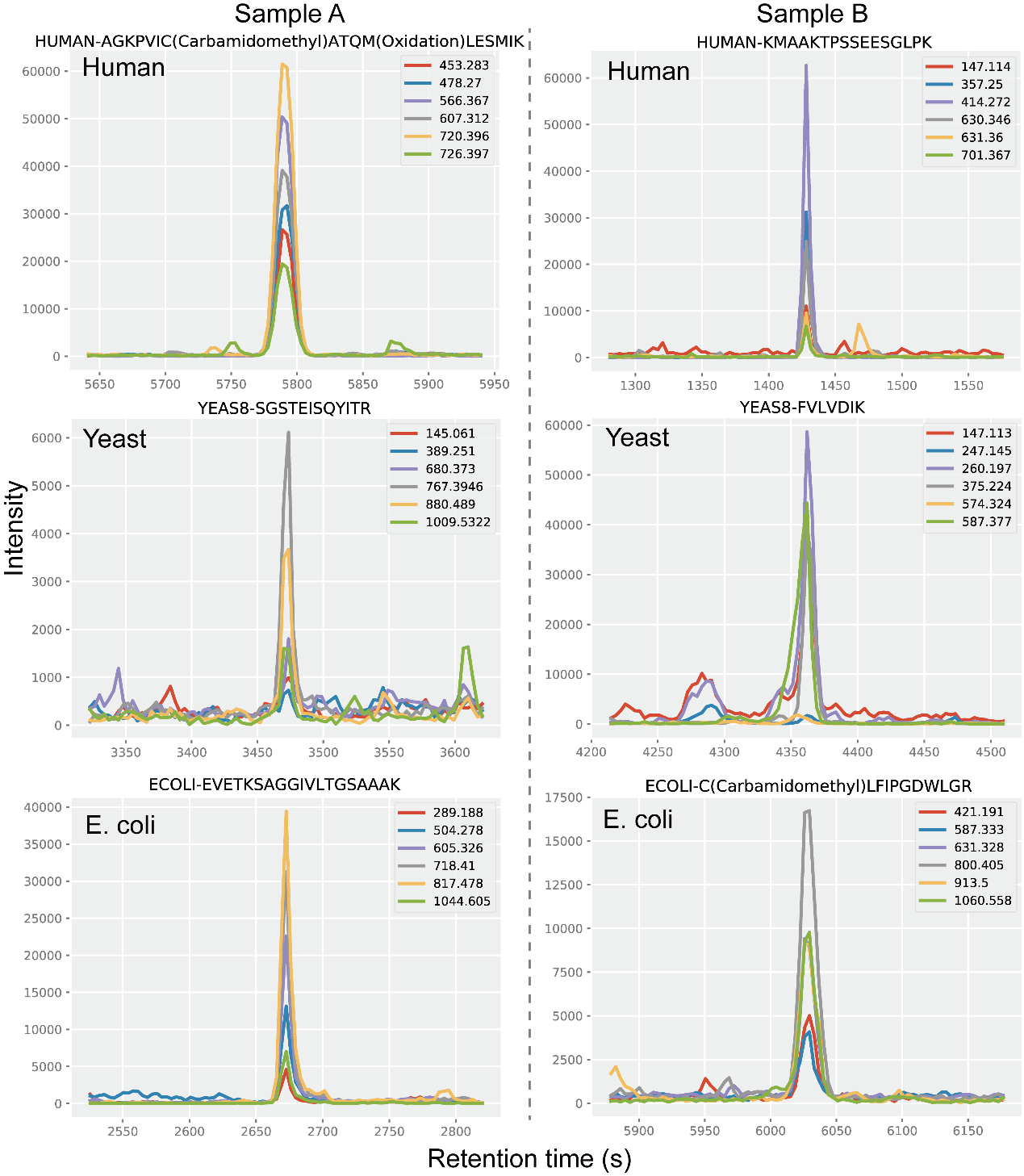


**Figure S14. The XICs of peptides identified in HYE124 TTOF6600 64var dataset by Dear-DIA^XMBD^ but not identified by Spectronaut14 and DIA-Umpire.** The colored lines are the XICs of fragments. X-axis is the retention time, and Y-axis is the intensity of XICs of fragments. The left and right panels show the peptides of sample A and sample B of HYE124 TTOF6600 64var, respectively. The legend of each subplot represent the m/z of the corresponding fragments.


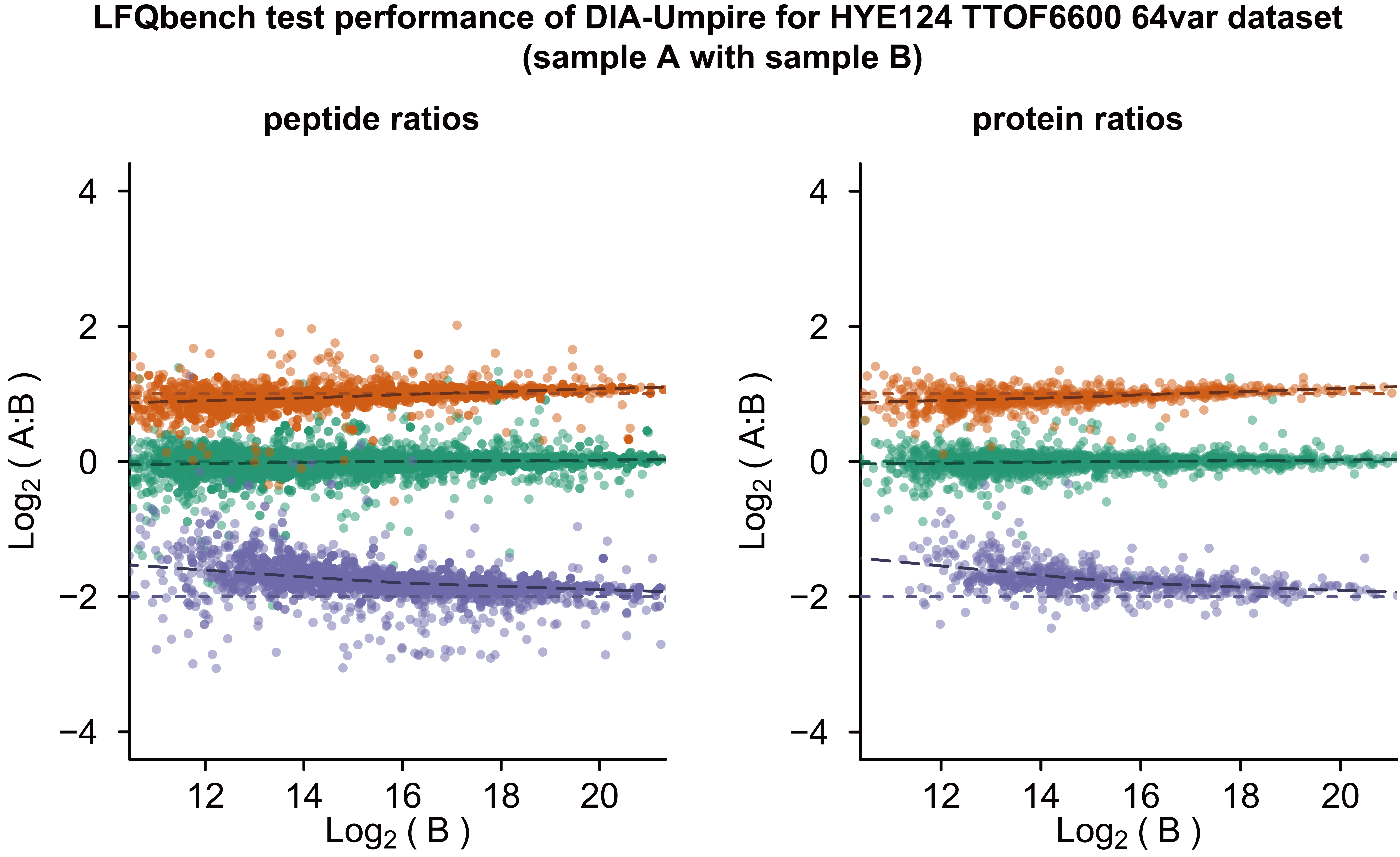


**Figure S15. LFQbench test performance of DIA-Umpire for HYE124 Triple TOF 6600 64var dataset.** The left and right scatter plots represent the peptide ratios and the protein ratios reported by DIA-Umpire, respectively. The colored dashed lines indicate the expected ratios for human (green), yeast (orange) and E. coli (purple). The black dashed lines represent the local trend along the x axis of experimental log-transformed ratios of each population (human, yeast and E. coli).


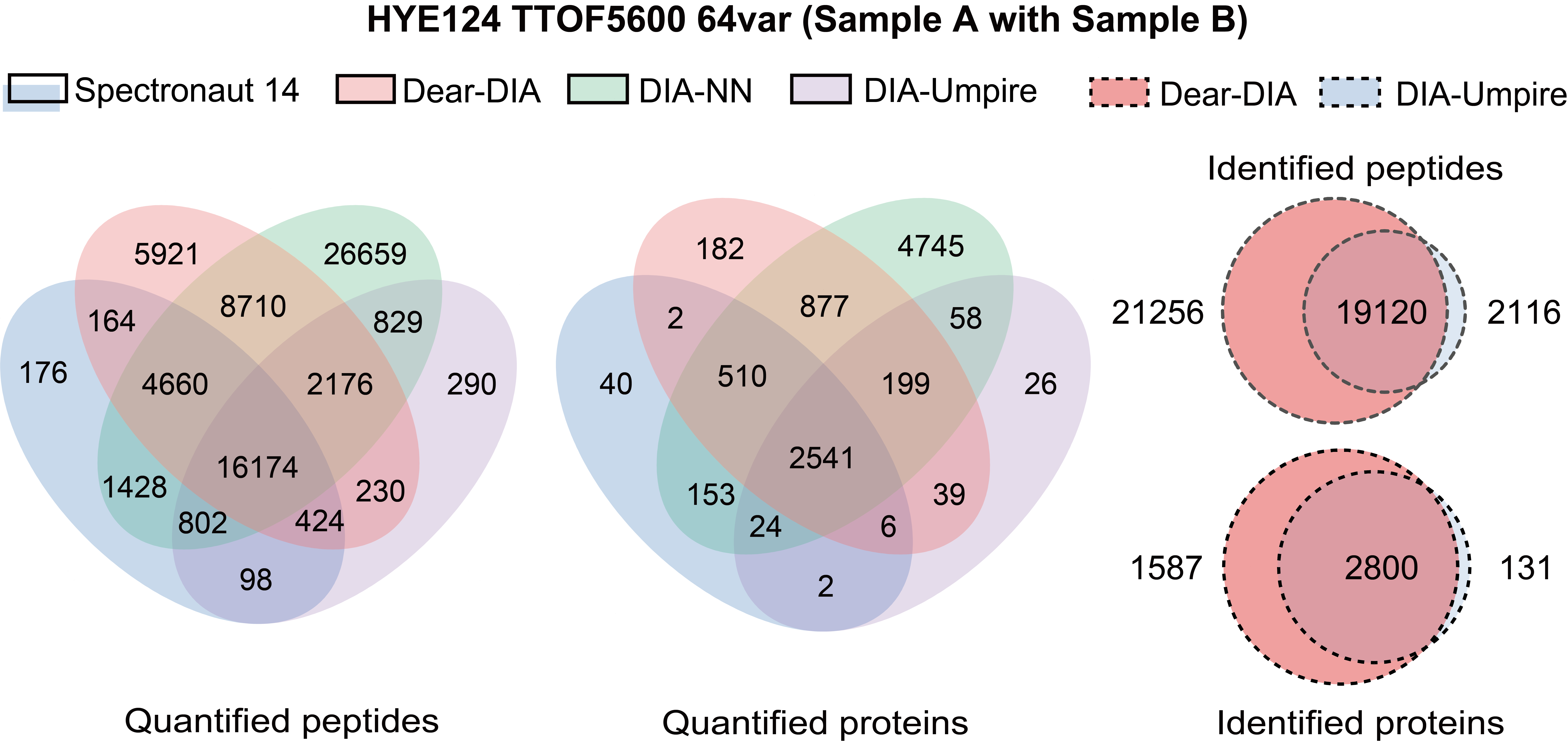


**Figure S16. Venn diagrams of peptides and proteins found from HYE124 Triple TOF 5600 64var dataset.** The comparison of the numbers of identified and quantified peptides and proteins obtained by Dear-DIA^XMBD^, DIA-Umpire, and Spectronaut 14 from HYE124 TripleTOF5600 64var dataset with samples A and B together. The solid lines and dashed lines show the quantified and identified results, respectively. The red circles, the dark blue circles and the light blue circles represent the results of Dear-DIA^XMBD^, Spectronaut 14, and DIA-Umpire, respectively.


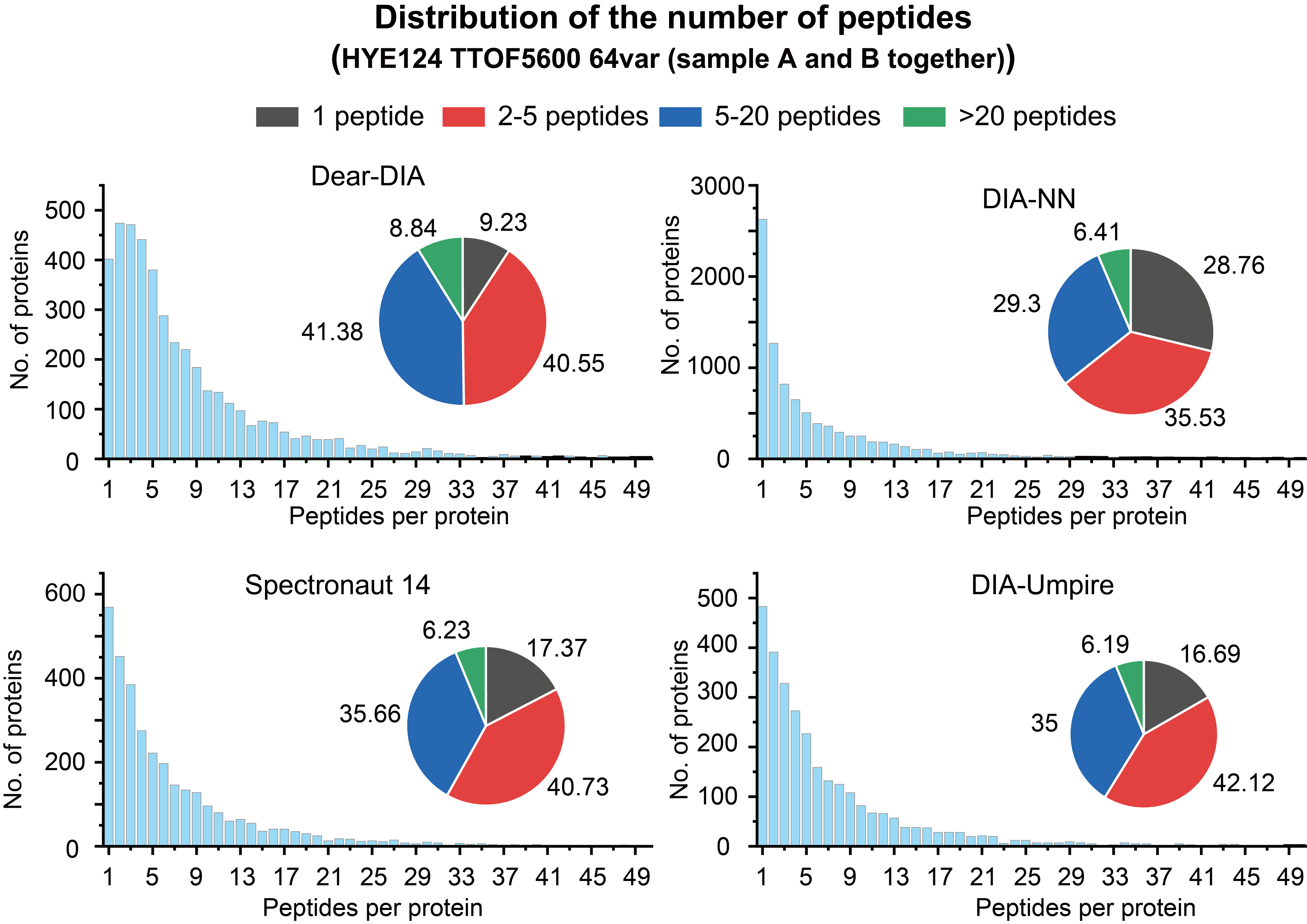


**Figure S17. Distribution of the number of peptides from HYE124 TOF5600 64var dataset.** The x-axis and y-axis represent the number of peptides and the number of proteins, respectively.


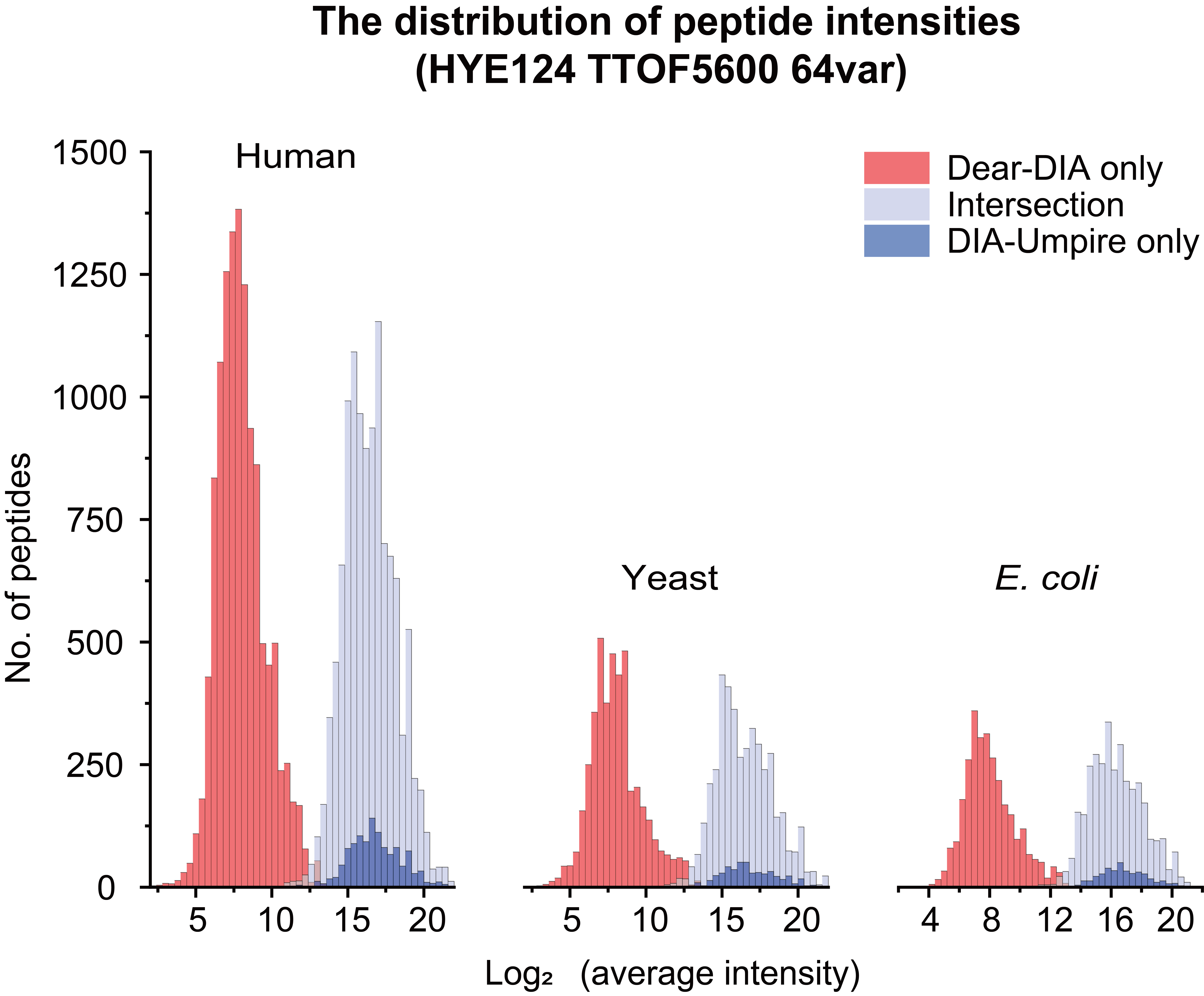


**Figure S18. Venn diagrams of peptides and proteins found from HYE124 Triple TOF 5600 64var dataset.** The log2-scaled distributions of quantified peptide intensities discovered from HYE124 TripleTOF 5600 dataset with samples A and B together. The peptides shared jointly with DIA-Umpire and Dear-DIA^XMBD^ are shown in light blue; the peptides reported exclusively by Dear-DIA^XMBD^ and by DIA-Umpire are shown in red and dark blue, respectively.


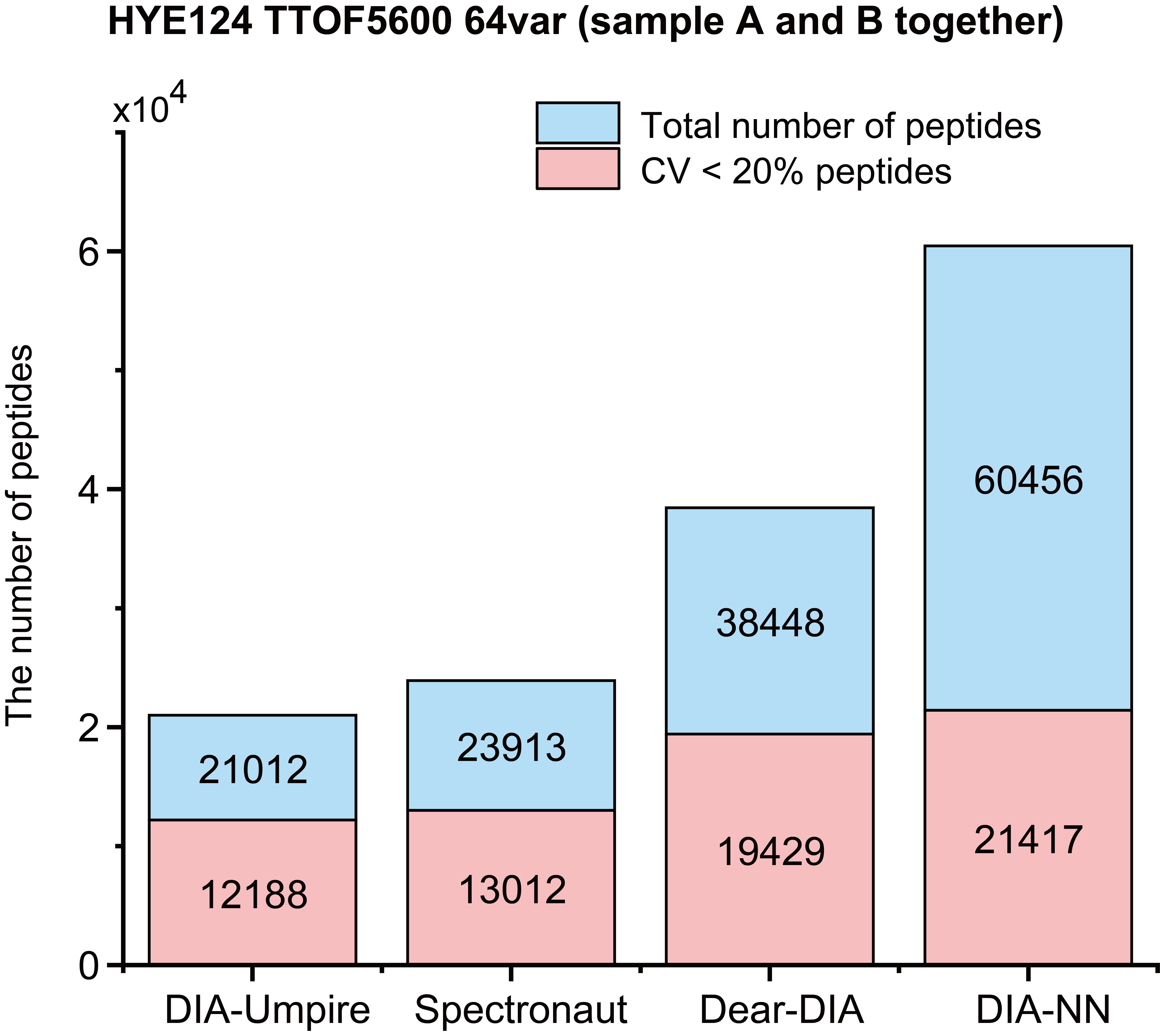


**Figure S19. The number of peptides with coefficient of variation (CV) below 20% from HYE124 TTOF5600 64var dataset (sample A with sample B).** The red and blue part represent the number of peptides with CV below 20% and the total number of peptides, respectively.





**Figure S20. LFQbench test performance of HYE124 Triple TOF 5600 64var dataset.** The top and down scatter plots represent the peptide ratios and the protein ratios reported by Dear-DIA^XMBD^, Spectronaut 14, and DIA-Umpire, respectively. The colored dashed lines indicate the expected ratios for human (green), yeast (orange), and E. coli (purple), respectively. The black dashed lines represent the local trend along the x-axis of experimental log-transformed ratios of each population (human, yeast and E. coli).


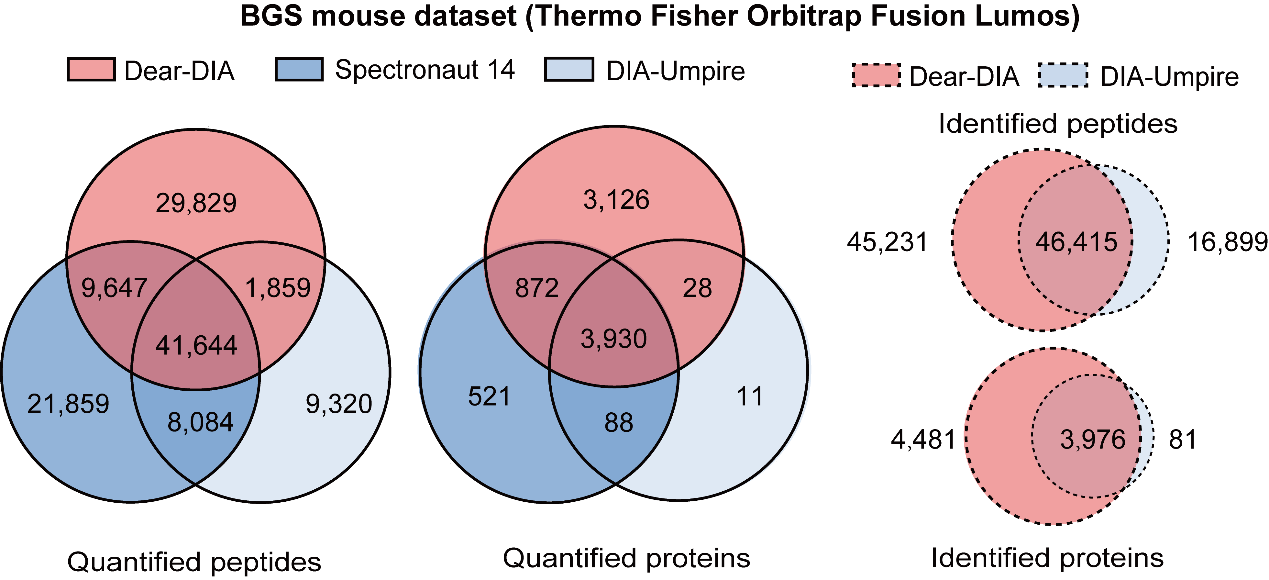


**Figure S21. Venn diagrams of peptides and proteins found from BGS mouse DIA dataset.** The comparison of the numbers of identified and quantified peptides and proteins obtained by Dear-DIA^XMBD^, DIA-Umpire, and Spectronaut 14 from BGS mouse DIA dataset. The solid lines and dashed lines show the quantified and identified results, respectively. The red circles, the dark blue circles and the light blue circles represent the results of Dear-DIA^XMBD^, Spectronaut 14, and DIA-Umpire, respectively.


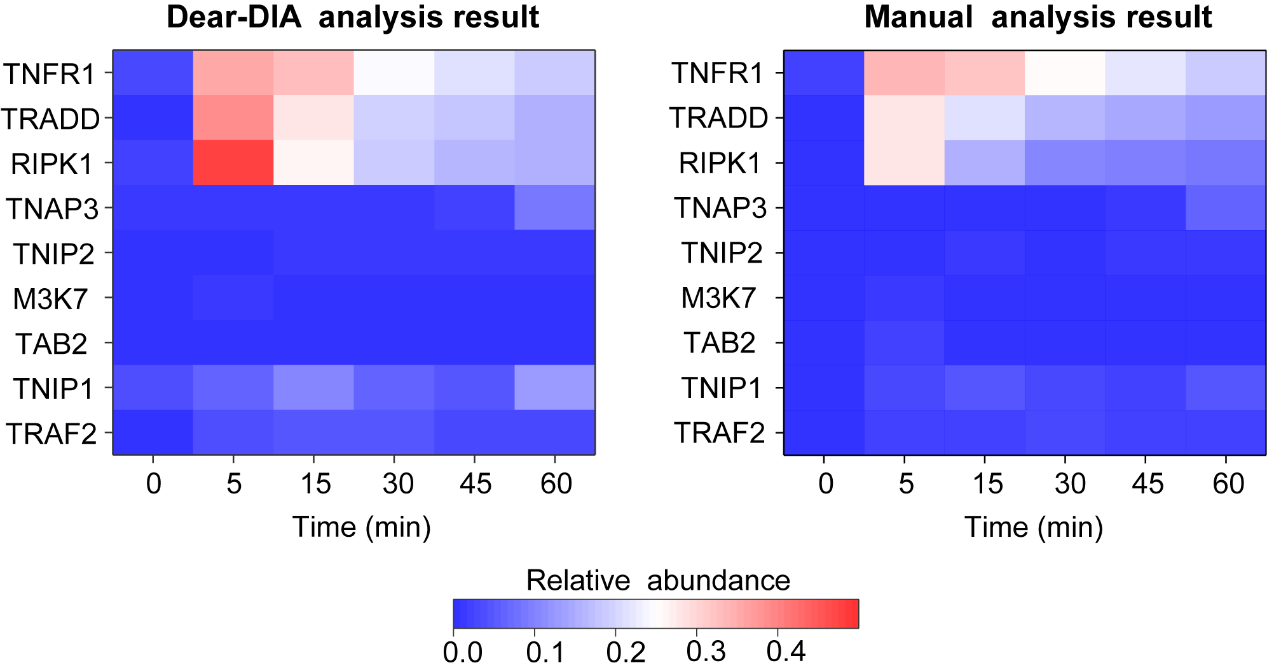


**Figure S22. The heat maps of protein intensities of Dear-DIA^XMBD^ and manual analysis in TNFR1 dataset.** The left and right panels show the results of Dear-DIA^XMBD^ and manual analysis, respectively. The color changed from blue to red indicates that the relative abundance of proteins increases from 0.0 to 0.5. The names of proteins which were confirmed by manual check are located on the left side of each subplot.


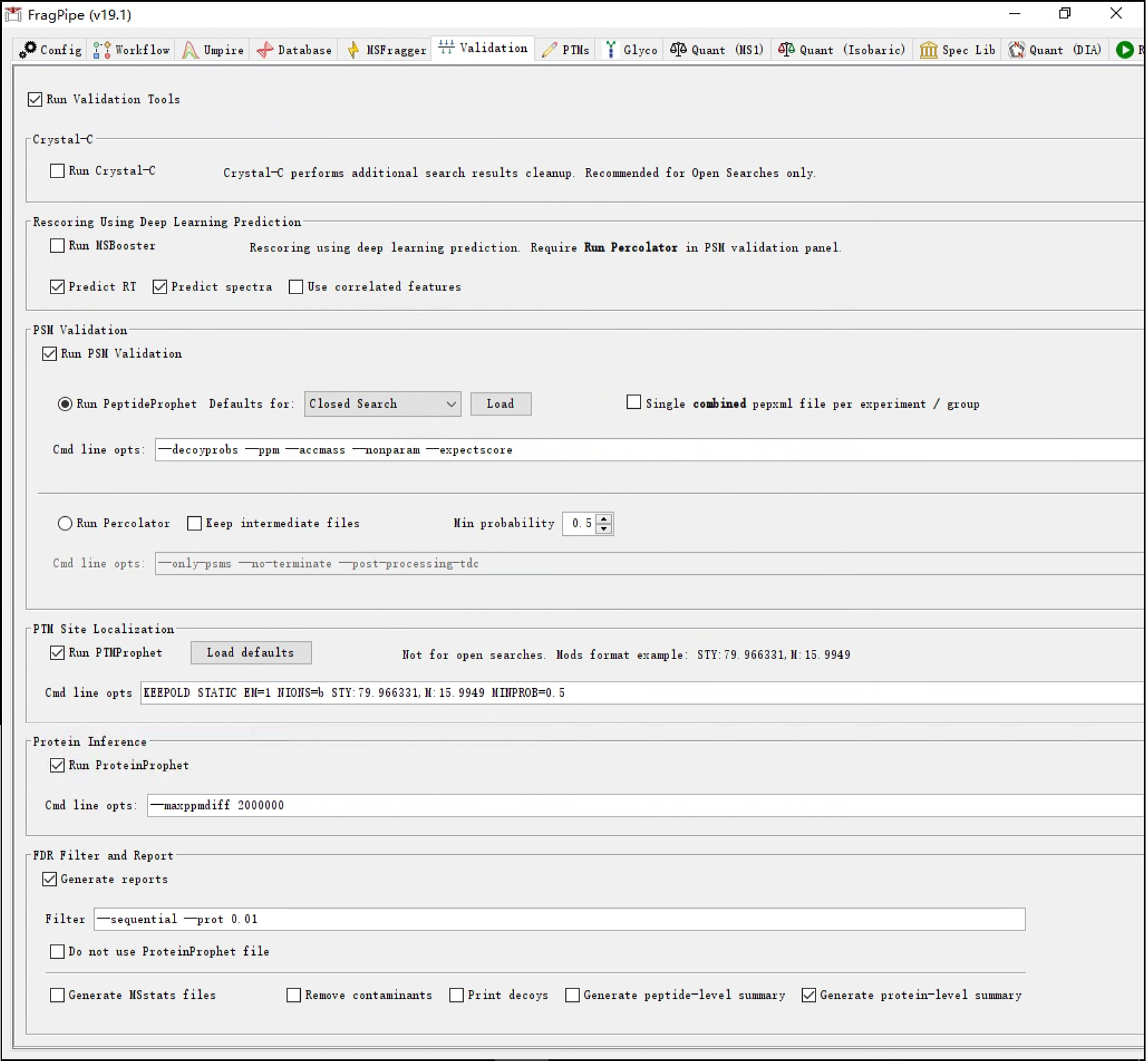


**Figure S23. The parameters of Philosopher (v4.8.1).** Parameter settings when using Philosopher as a validation toolkit in Dear-DIA^XMBD^ analysis workflow.


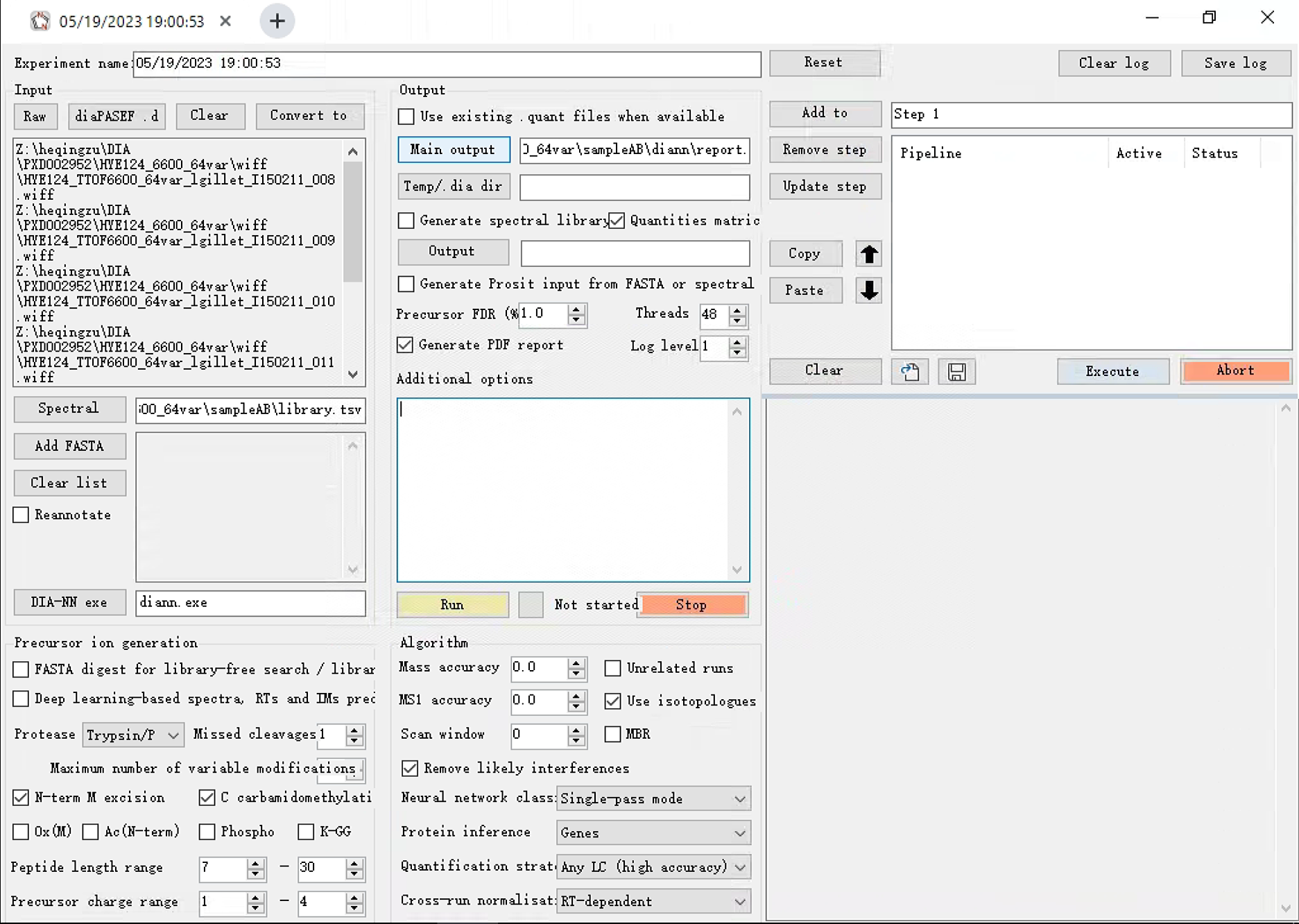


**Figure S24. The parameters of DIA-NN (v1.8.1).** Parameter settings when using DIA-NN as a quantitative toolkit in Dear-DIA^XMBD^ analysis workflow.

**Table S1. The LFQbench metrics of peptides and proteins found by Dear-DIA^XMBD^, Spectronaut 14 and DIA-Umpire of HYE124 64-var dataset (TripleTOF 6600).**

| Dear-DIA^XMBD^ | | | | | | |
| --- | --- | --- | --- | --- | --- | --- |
|  | $`A:B` (peptides) | | | $`A:B` (proteins) | | |
|  | HUMAN | YEAST | ECOLI | HUMAN | YEAST | ECOLI |
| Invalid ratios | 1480 | 655 | 708 | 17 | 5 | 10 |
| invalid, out of validity range | 0 | 0 | 0 | 0 | 0 | 0 |
| invalid, missing value | 1480 | 655 | 708 | 17 | 5 | 10 |
| valid ratios | 39416 | 13577 | 8740 | 3023 | 1164 | 855 |
| in plot range | 39416 | 13577 | 8740 | 3023 | 1164 | 855 |
| out of plot range | 0 | 0 | 0 | 0 | 0 | 0 |

| Spectronaut 14 | | | | | | |
| --- | --- | --- | --- | --- | --- | --- |
|  | $`A:B` (peptides) | | | $`A:B` (proteins) | | |
|  | HUMAN | YEAST | ECOLI | HUMAN | YEAST | ECOLI |
| Invalid ratios | 86 | 45 | 66 | 1 | 2 | 5 |
| invalid, out of validity range | 0 | 0 | 0 | 0 | 0 | 0 |
| invalid, missing value | 86 | 45 | 66 | 1 | 2 | 5 |
| valid ratios | 31453 | 11868 | 8294 | 2987 | 1172 | 856 |
| in plot range | 31437 | 11862 | 8263 | 2987 | 1172 | 856 |
| out of plot range | 16 | 6 | 31 | 0 | 0 | 0 |

| DIA-Umpire | | | | | | |
| --- | --- | --- | --- | --- | --- | --- |
|  | $`A:B` (peptides) | | | $`A:B` (proteins) | | |
|  | HUMAN | YEAST | ECOLI | HUMAN | YEAST | ECOLI |
| Invalid ratios | 134 | 55 | 77 | 6 | 2 | 8 |
| invalid, out of validity range | 0 | 0 | 0 | 0 | 0 | 0 |
| invalid, missing value | 134 | 55 | 77 | 6 | 2 | 8 |
| valid ratios | 16868 | 6426 | 4695 | 1929 | 762 | 557 |
| in plot range | 16868 | 6426 | 4695 | 1929 | 762 | 557 |
| out of plot range | 0 | 0 | 0 | 0 | 0 | 0 |

**Table S2. The parameters of MSFragger search engines.**


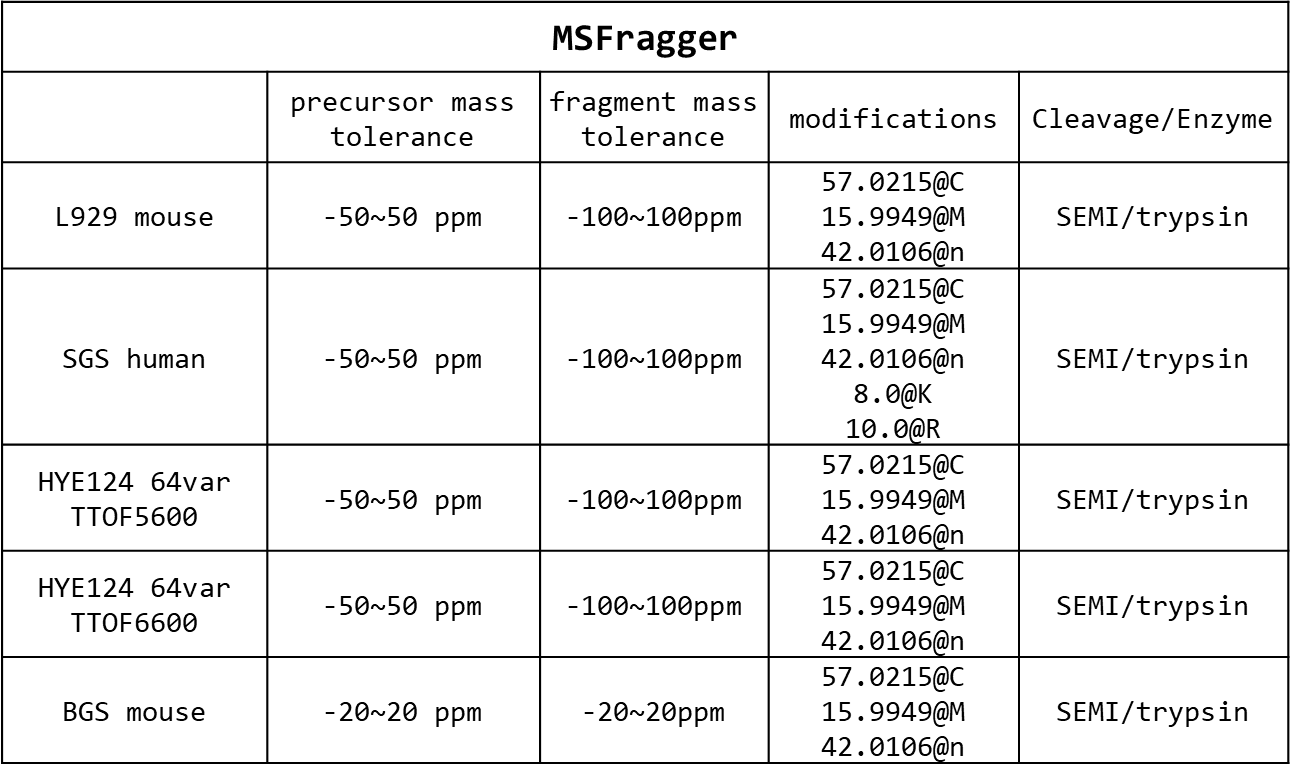


* Except for the parameters in the table, the rest of the parameters uses the default value.

**Table S3. The parameters of Dear-DIA^XMBD^.**


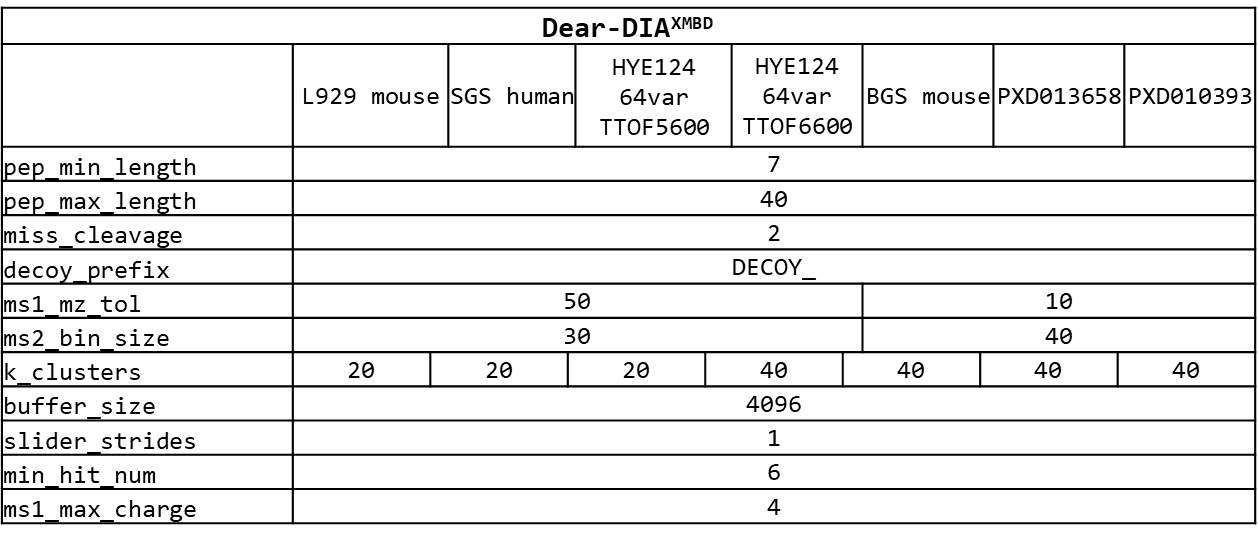


“pep_min_length”: the minimum length of digested peptides. The default is 7.

“pep_max_length”: the maximum length of digested peptides. The default is 40.

“miss_cleavage”: miss cleavage of enzyme digestion. The default is 2.

“decoy_prefix”: the prefix of decoy protein in fasta database file. The default is DECOY_.

“ms1_mz_tol”: the tolerance of precursor m/z tolerance (Unit: ppm). This parameter depends on the instrument resolution, and different instruments have different MS1 tolerance. For example, the MS1 tolerance of the AB SCIEX 5600 and Thermo Orbitrap mass spectrometers is 50ppm and 10ppm, respectively. The default is 50.

“ms2_bin_size”: the binning size of MS2 m/z. The high-resolution mass spectrometer can be set to a larger bin size. The value of this parameter is dependent on the resolution of the instrument, with higher resolution instruments corresponding to larger parameter values. The suggested parameter values are 30, 40, and 50. The larger value of "ms2_bin_size" will cost more memory space and time. The default is 30.

“k_clusters”: the number of clusters (k clusters) using in k-means. The default value of k is set to 20. We estimated the value of k based on the experience and the test results. The number of fragments contained in a DDA spectrum is about 200. Correspondingly, there are approximately 5000 fragments in a slider after data preprocessing. To product the pseudo-DDA spectra, the k-means clustering algorithm divides the fragments in a slider into k categories, and each category contains 200 fragments on average. Therefore, the value of k is about 25. We tried the value of k from 2 to 100, and found that the best number of peptides correspondes to k=20. In addition, the value of k is an adjustable parameter that the users can optimize by themselves. We recommend that the value of k should not be greater than 50.

“buffer_size”: the size of buffer space when loading mzXML file. The larger the value of "buffer_size" is, the more memory will be occupied. The default is 4096 Bytes (4 KB).

“slider_strides”: the overlap of neighboring sliders. The default is 1.

“min_hit_num”: the number of hit b ions plus the number of hit y ions. The default is 6.

“ms1_max_charge”: the maximum charge of precursor. The default is 4.

**Table S4. The parameters of Spectronaut 14.**


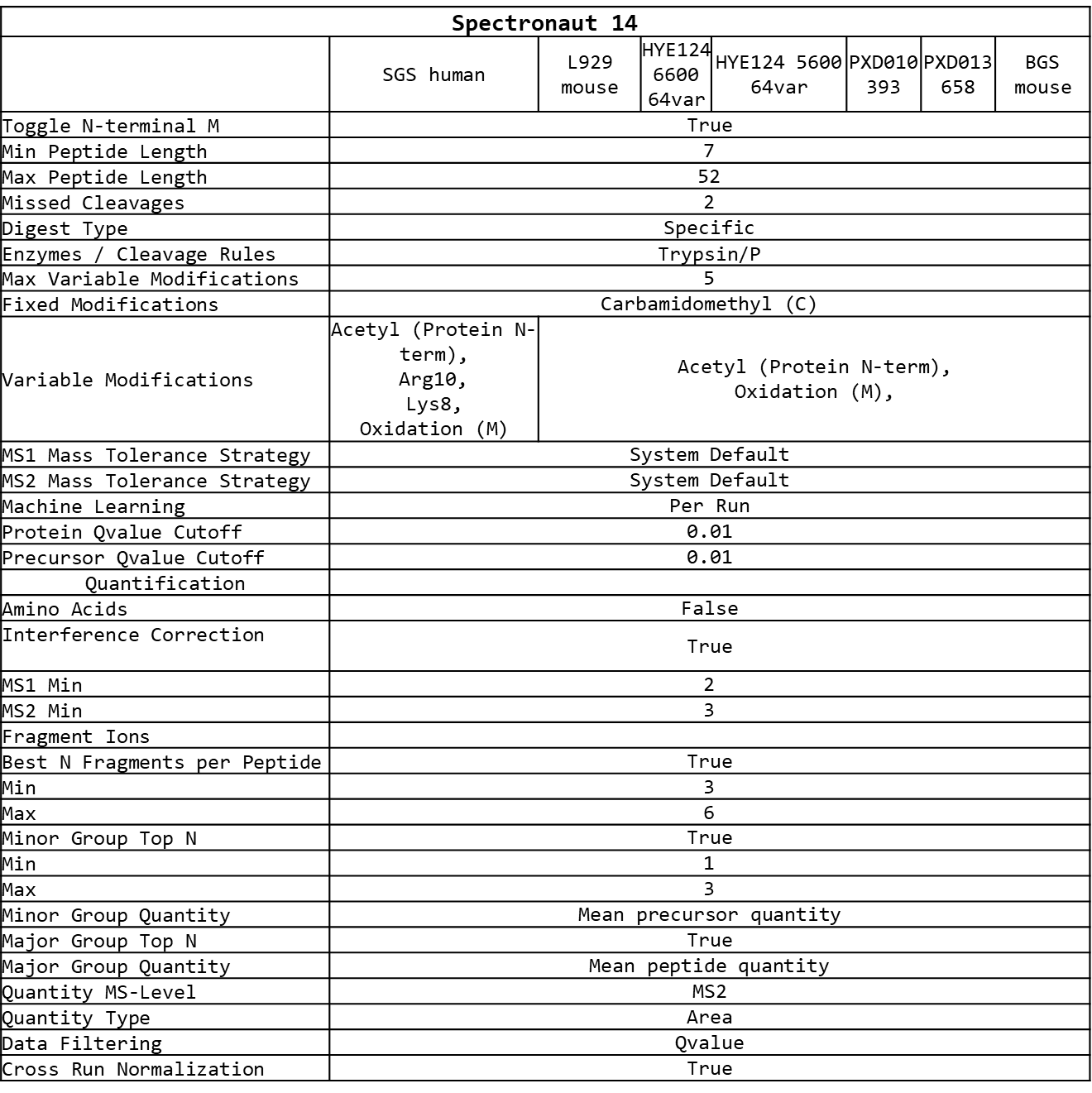


**Table S5. The parameters of DIA-Umpire.**


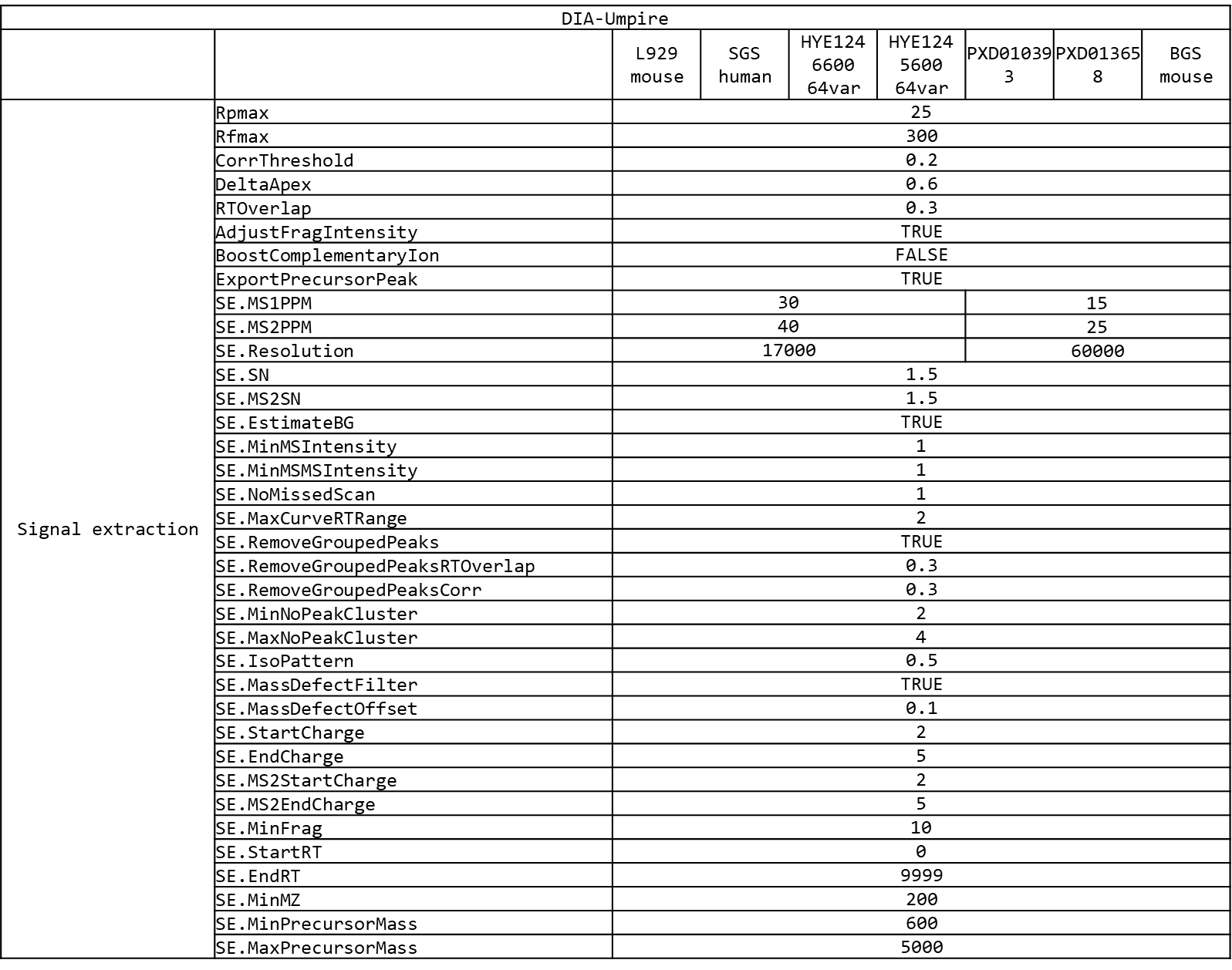


**Supplementary Text S1. The times required for training and running Dear-DIA^XMBD^, DIA-Umpire and Spectronaut 14.**

We test Dear-DIA^XMBD^ on a high-performance computer. The testing results are shown in the following:

**CPU*:** Intel(R) Xeon(R) 8280M CPU @ 2.60GHz 64-Core

**GPU*:** NVIDIA GeForce GTX 1080Ti

**Memory**: 256GB

1. The training time of Dear-DIA^XMBD^: about 2 days.
2. The running time of Dear-DIA^XMBD^:

| File size | Only CPU* | CPU*+GPU* |
| --- | --- | --- |
| 34GB (profile mzXML, AB Sciex) | ~35 minute | ~30 minute |

1. The running time of DIA-Umpire:

| File size | DIA-Umpire |
| --- | --- |
| 34GB (profile mzXML, AB Sciex) | ~40 minute |

1. The running time of Spectronaut 14:

| File size | Spectronaut 14 |
| --- | --- |
| 34GB (profile mzXML, AB Sciex) | ~2.8 hours |

**Supplementary Text S2. The comparison between VAE and a simplistic precursor-fragment grouping algorithm.**

We have implemented the simplistic precursor-fragment ion grouping algorithm from DIA-Umpire within Dear-DIA^XMBD^. The grouping algorithm selects top N with N=50 precursor ions by intensity, and then calculates Pearson correlation coefficients between top N precursor ions and all fragment ions. We divided the precursor-fragment pairs with Pearson coefficient greater than 0.6 into the same group.

We used L929 mouse dataset as the testing dataset to evaluate the performance of the deep learning and the simplistic signal processing algorithm. The performances of Dear-DIA^XMBD^ with VAE and with the simplistic grouping algorithm are compared in the following Venn diagram, indicating that Dear-DIA^XMBD^ with VAE discovers more peptides and proteins than the simplistic grouping algorithm.


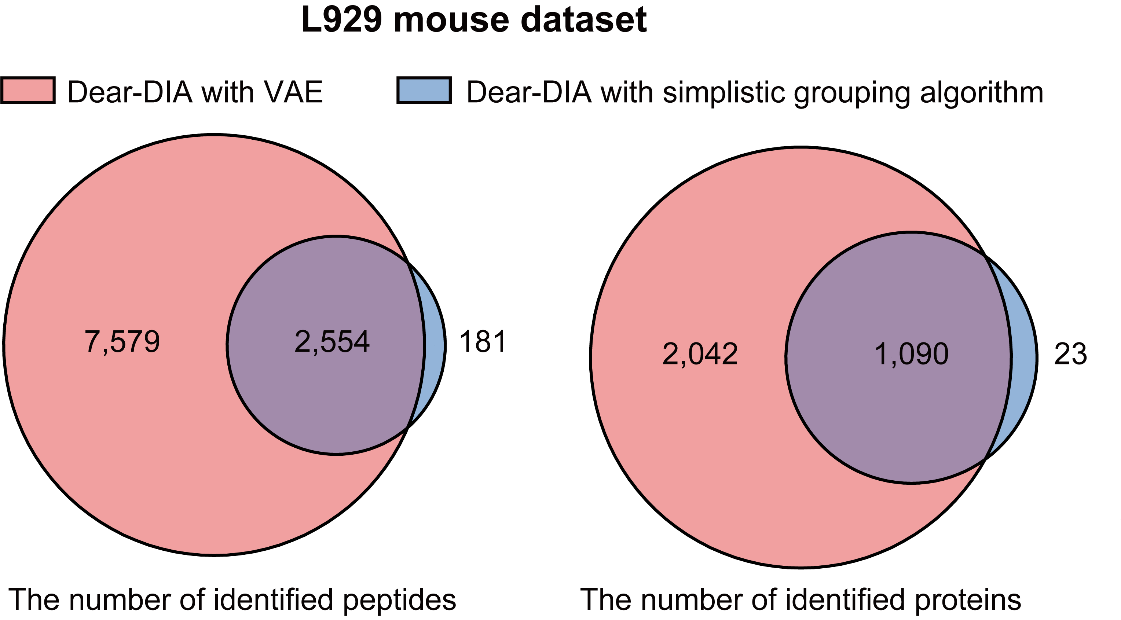


**Supplementary Text S3. The comparison results between Dear-DIA^XMBD^ with PIndex and Dear-DIA^XMBD^ without PIndex.**

We removed PIndex algorithm and used all-vs-all method to generate pseudo-spectra. We used Dear-DIA^XMBD^ without PIndex and Dear-DIA^XMBD^ with PIndex to analyze L929 mouse dataset, respectively. According to the identified results, the peptides found by Dear-DIA^XMBD^ without PIndex and with PIndex are 7,669 and 9,545, and the proteins found by Dear-DIA^XMBD^ without PIndex and with PIndex are 2,202 and 2,673, respectively. Dear-DIA^XMBD^ without PIndex covers 70% peptides and 78% proteins found by Dear-DIA^XMBD^ with PIndex.


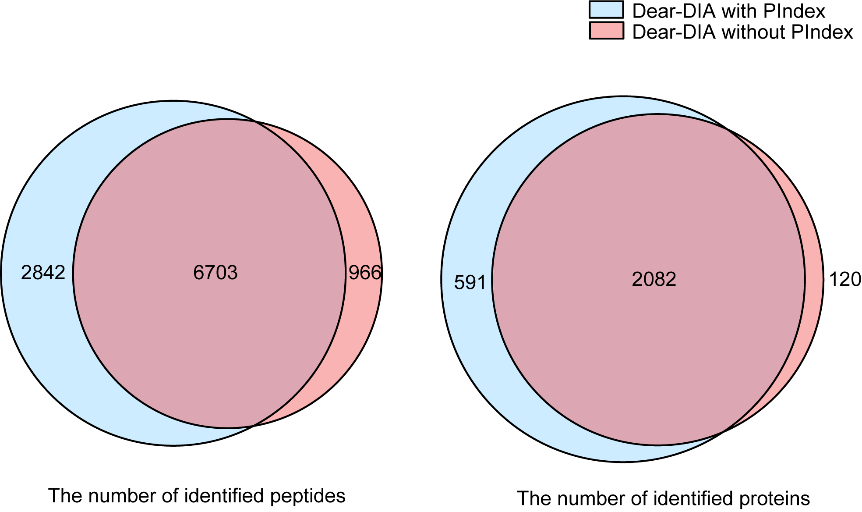


**Supplementary Text S4. The results of simplistic training VAE using Obitrap data and QTOF data.**

We trained VAE model on a Biognosys facility (BGS) dataset (*46*) (mouse cerebelli) and an *E. coli* dataset. The BGS dataset was acquired from Orbitrap Fusion Lumos mass spectrometer (Thermo Fisher Scientific, San Jose, CA) and the *E. coli* dataset (*43*) was acquired from TripleTOF 6600 QTOF mass spectrometer (AB Sciex). These two datasets were downloaded from the ProteomeXchange Consortium (http://proteomecentral.proteomexchange.org) via the PRIDE partner repository with the data set identifiers PXD011691 and PXD020761.

We applied DIA-Umpire to analyze the BGS dataset to generate the BGS spectral library. Then we used OpenSWATH workflow to quantify the peptides included in BGS library and *E. coli* library, respectively. According to the report files of OpenSWATH workflow, we extracted the fragments XICs of quantified peptides to establish the triplet training datasets of BGS and *E. coli*, respectively. The training set was a rough dataset without manual inspection. Finally, we trained the Orbitrap VAE and QTOF VAE models on BGS dataset and *E. coli* dataset, respectively.

To demonstrate the transferability of VAE model, we used QTOF VAE and Orbitrap VAE to analyze a single HeLa data (2 hours run, Thermo Fisher Q Exactive HF-X). The HeLa dataset (*58*) was downloaded from the ProteomeXchange Consortium via the PRIDE partner repository with the dataset identifier PXD013658.

According to the identified results, Orbitrap VAE finds 3,3162 peptides and 6,492 proteins, while QTOF VAE reports 3,0487 peptides and 6,107 proteins, respectively. Orbitrap VAE finds 8% more peptides and 6% more proteins with Orbitrap data. Using the Orbitrap VAE model to analyze the Orbitrap data can indeed find more peptides and proteins. We will consider adding the Obitrap data to train the model to improve the performance of Dear-DIA^XMBD^ in our future work.


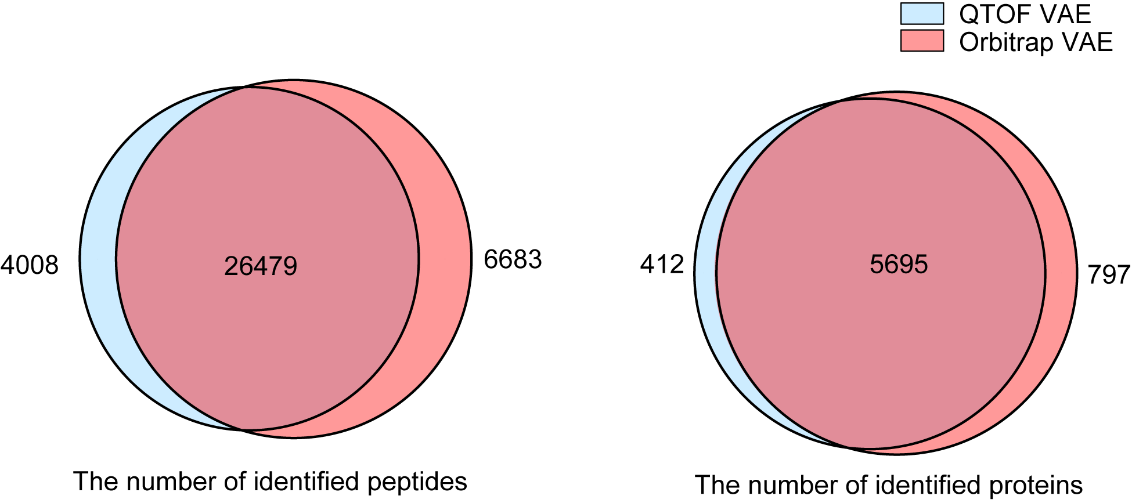


**Supplementary Text S5. The limitations of Pearson correlation coefficient.**

DIA-Umpire applies Pearson correlation coefficient to estimate the similarity between precursor XIC and fragment XICs to obtain the precursor-fragment groups. The formula of Pearson coefficient is shown in the following:

$$\rho_{X,Y}=\frac{\sum_{i=1}^{n} \left(X_{i}-\bar{X}\right)\left(Y_{i}-\bar{Y}\right)}{\sqrt{\sum_{i=1}^{n} \left(X_{i}-\bar{X}\right)^{2}}\sqrt{\sum_{i=1}^{n} \left(Y_{i}-\bar{Y}\right)^{2}}}$$

where $X$and $Y$ represent two variables that need to be calculated for correlation. $\bar{X}$ and $\bar{Y}$ are the mean values of $X$and $Y$, respectively. $n$ is the size of $X$and $Y$. Pearson correlation coefficient is a linear correlation coefficient, which can only measure the linear relationship between $X$and $Y$.


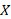

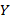

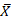

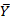

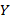

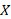

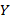

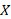

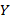


The Pearson correlation coefficient can represent the similarity between two normally distributed and linearly related XICs, such as the X and Y shown in the following figure.


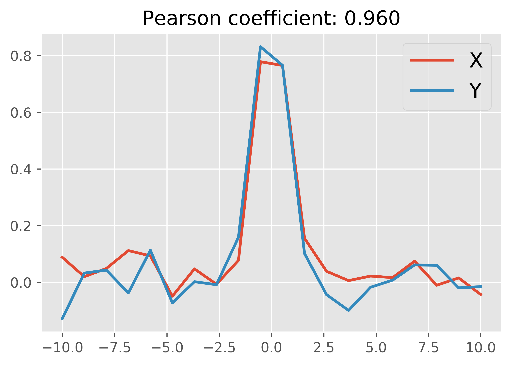


However, there are several limitations for Pearson correlation coefficient. To show the details of the limitations, we used computer simulation to generate several XICs, and then calculated the Pearson coefficient between them.

Pearson correlation coefficient is sensitive to outliers. For instance, we only change the last value of Y and recalculate the Pearson correlation coefficient. One can find that Pearson’s coefficient drops from 0.96 to 0.351, even though Y contains only one outlier.


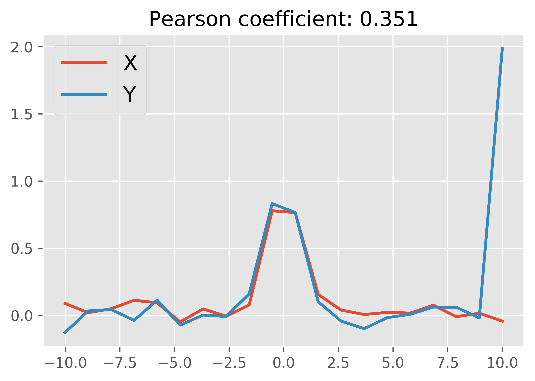


According to the following figure, the similarity calculated by Pearson correlation coefficient is not accurate when there is a misalignment between two XICs.


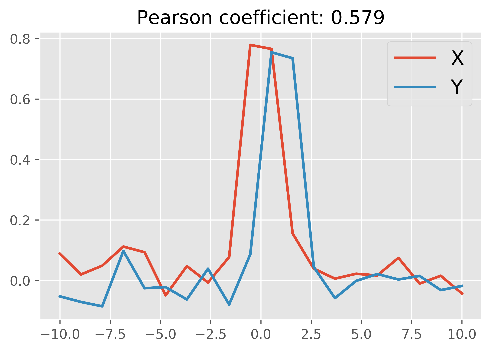


In addition, the interference peaks can affect the estimation of similarity by Pearson correlation coefficient.


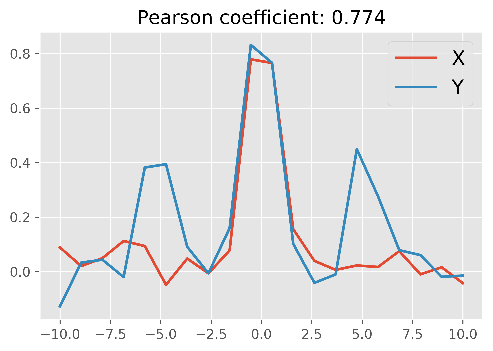


Dear-DIA^XMBD^ employs VAE to extract the high dimensional statistic features for each fragment XIC. Compared to Pearson correlation coefficient, we used the features to replace the corresponding XICs and then applied Euclidean distance to measure the similarity between the features. The features extracted by VAE are learned from a large amount of data. It shows a better robustness and can avoid the influence of outliers, misalignment and interference peaks.

**Supplementary Text S6. A comparison between the Gaussian curve fitting model and the VAE model.**

We written a Gaussian curve fitting program to replace the neural network. The Gaussian curve fitting algorithm can estimate the mean value and the variance of a fragment XIC. The fragment XICs are normalized by min-max scaling algorithm, which is described by the formula:

$$x'=\frac{x-x_{\min}}{x_{\max}-x_{\min}}$$

where$x$ and $x'$ represent the intensity of fragment XIC before and after normalization, respectively. The values $x_{\min}$ and $x_{\max}$ represent the minimum and the maximum of XIC, respectively. Then we assume that the shape of fragment XIC shows similar to a single Gaussian peak, which can be described by the following common formula:


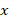

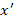

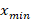

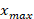


where $a$, $b$ and $c$ are the parameters of Gaussian curve. We regarded $a$, $b$ and $c$ as the features of the XIC curve, and used them to replace the features extracted by the neural network in the Dear-DIA workflow. We choose the L929 mouse dataset as the testing dataset for comparing the performance of Gaussian curve fitting algorithm and neural network model. The results are shown in the following Venn diagram.


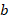

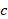

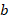

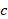

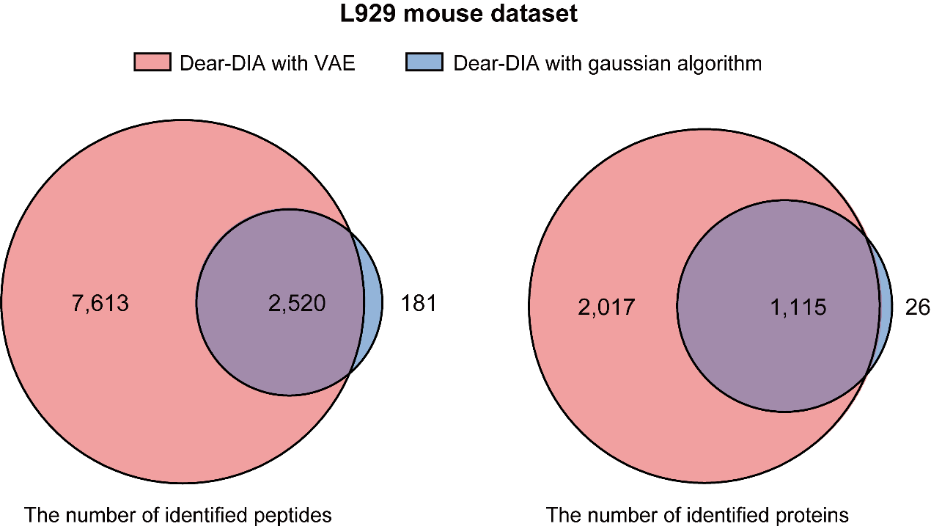


Dear-DIA^XMBD^ with the Gaussian curve fitting algorithm finds only 2,701 peptides and 1,141 proteins, while Dear-DIA^XMBD^ with neural network finds 10,133 peptides and 3,132 proteins. We believe that the shape of XIC curve cannot be simply represented by a Gaussian function. The deep neural network can extract the nonlinear features in high dimension space, and such features can be used to distinguish the noisy XICs.

**Supplementary Text S7. The FDR validation of Dear-DIA^XMBD^, DIA-Umpire, and Spectronaut 14.**

We validated FDR by searching the human samples against a merged sequence database containing all human and Arabidopsis proteins. The ratio of Arabidopsis peptides identified to the total number of peptides identified is the estimate of the FDR. We used two datasets to validate the FDR of Dear-DIA^XMBD^, Spectronaut 14 and DIA-Umpire.

We drew the plots containing “Number of target precursor ions” vs “q value” to show the FDR validation.

The dataset (2-hour and 6-hour gradient HeLa runs) was downloaded from the ProteomeXchange Consortium (http://proteomecentral.proteomexchange.org) via the PRIDE partner repository with the data set identifiers PXD013658. The raw files of 2-hour and 6-hour gradient Hela runs are shown in the following list:

“G_D190220_S467-570CSH17-60cm-GradientRamp-Hela-2ug-2h_MHRM_R01_T0.raw”,

“G_D190220_S467-570CSH17-60cm-GradientRamp-Hela-2ug-2h_MHRM_R02_T0.raw”,

“G_D190220_S467-570CSH17-60cm-GradientRamp-Hela-2ug-2h_MHRM_R03_T0.raw”,

“G_D190220_S467-570CSH17-60cm-GradientRamp-Hela-2ug-6h_MHRM_R01_T0.raw”,

“G_D190220_S467-570CSH17-60cm-GradientRamp-Hela-2ug-6h_MHRM_R02_T0.raw”,

“G_D190220_S467-570CSH17-60cm-GradientRamp-Hela-2ug-6h_MHRM_R03_T0.raw”.

We treated the precursor ions with the same peptide sequence, modifications and charge as unique ones such as “HSAPGLLSM[147]ANSGPSTNGCQFFITCSK_3”. Then we drew plots containing “Number of target precursor ions” vs “q value” curves and the “Number of decoy precursor ions” vs “q value” curves.

We manually calculated the q-values for the outputs from Comet search engine. The "expect" scores output by Comet were used to rank the precursor ions and calculate the q-value. The smaller the "expect" value is, the higher the ranking of the precursor ion is.

According to the description from the following paper: “Unbiased False Discovery Rate Estimation for Shotgun Proteomics Based on the Target-Decoy Approach^3^”, we used three steps to calculate the q-values.

**Step 1.** We sorted the list of peptide precursor ions according to the “expect” scores included in the Comet outputs.

**Step 2.** we estimate FDR using the following formula:

$$FDR=\frac{d}{t}$$

where *d* and *t* are the numbers of decoy and target precursor ions in the set, respectively. The peptides from Human are treated as targets, while the peptides from Arabidopsis are treated as decoys.

**Step 3.** We calculated q-values for precursor ions using the above-mentioned definition as follows:

$$q_{i}=\min_{j\geq i} FDR_{j}$$

By definition, the calculated q-values grow monotonically in the list of sorted peptide precursor ions. The results of q-value for Dear-DIA^XMBD^, Spectronaut 14, and DIA-Umpire are shown in the following figures.





**Supplementary Text S7 Figure 1 |** The q-value plots for Dear-DIA^XMBD^, Spectronaut 14 and DIA-Umpire from HeLa dataset (2-hour gradient and 6-hour gradient). The red and blue curves show the q-values of target precursor ions and decoy precursor ions, respectively. The subplots represent the complete q-value results of Spectronaut.

**Supplementary Text S8. The installation instruction of Dear-DIA^XMBD^.**

1. **Windows users run the release version of Dear-DIA using GUI.**
2. Double click “Dear-DIA-GUI.exe”.


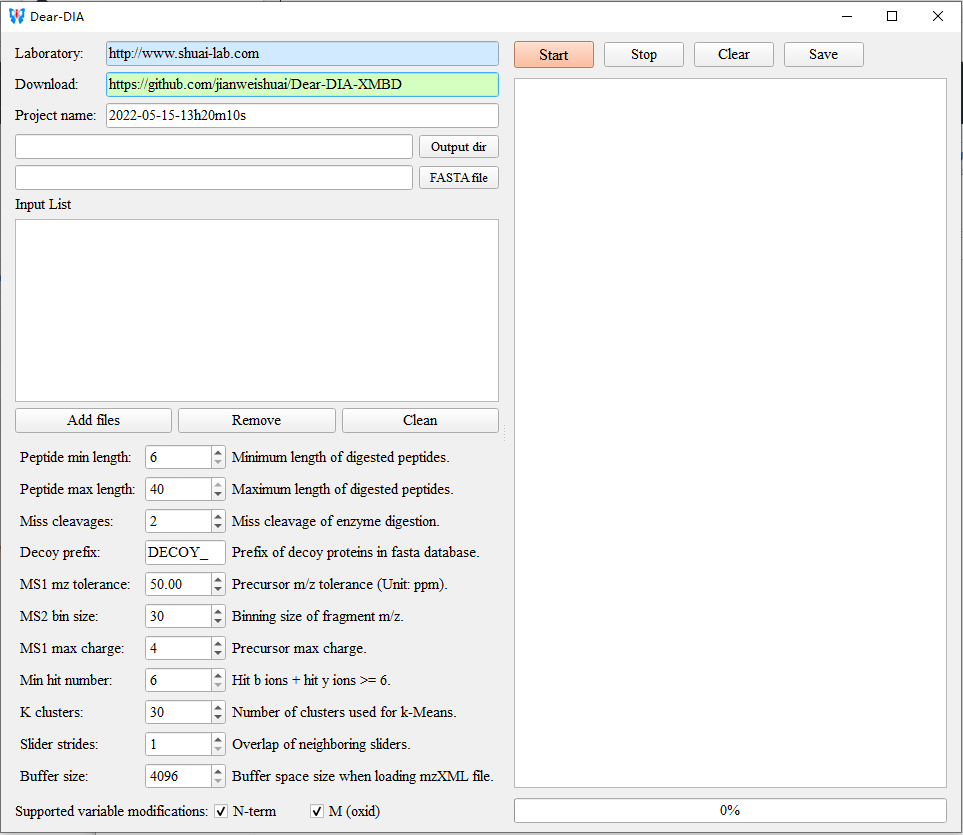


1. Set the parameters of “Ouput dir”, “FASTA file”. Then click the button “Add files” to add the profile mzXML files. The users can also set others parameters on the bottom of panel.


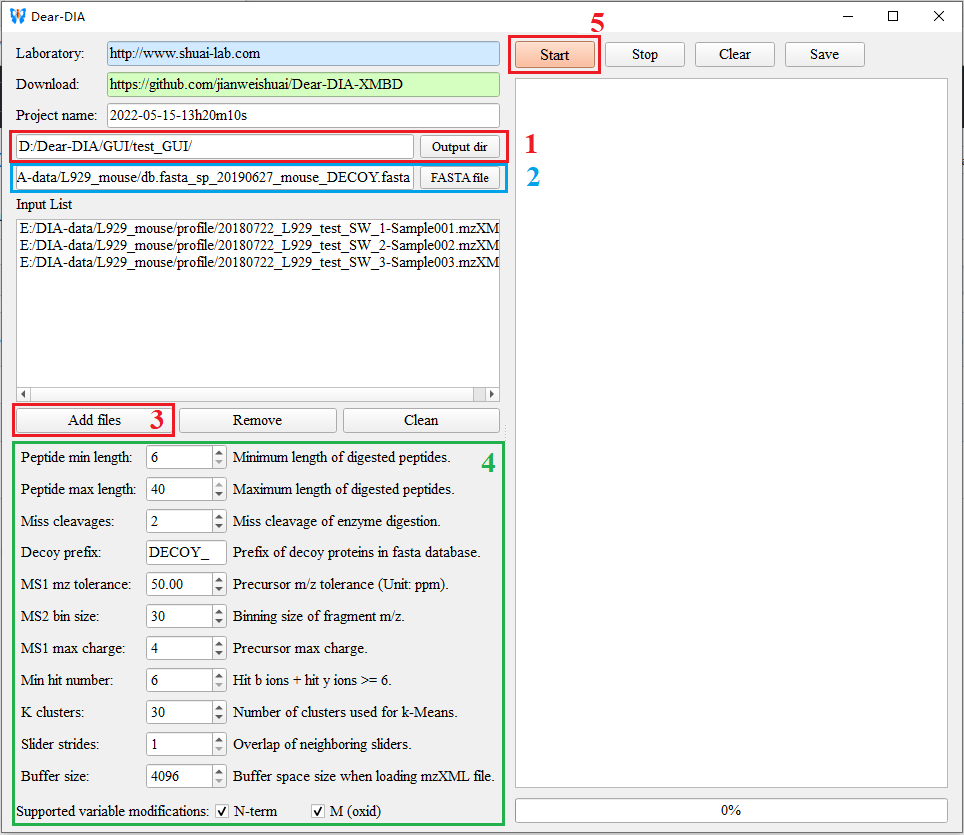


1. Run the Dear-DIA workflow.

Click “Start” push-button to run the Dear-DIA workflow. The output file with “.mgf” suffix can be found in the setting output directory.

1. **Windows users run the release version of Dear-DIA using command line (without GUI).**
2. Open the “cmd.exe”.


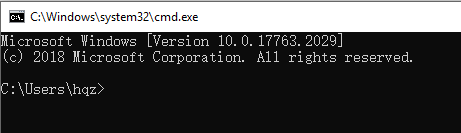


1. Entry to the folder included “Dear-DIA.exe”.

The users can use the command such as “cd /d C:\Users\hqz\Desktop\test_deardia\windows_gpu_release” to entry the folder. The path “C:\Users\hqz\Desktop\test_deardia\windows_gpu_release” indicates the folder containing “Dear-DIA.exe”


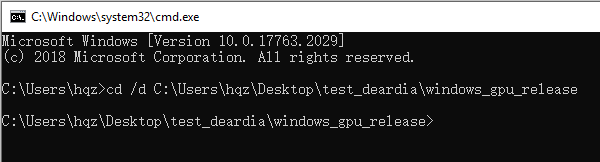


1. If there is no configure file of Dear-DIA such like “deardia.configure.new”, the users can use the command “Dear-DIA.exe --p” to generate a new configure file.

Note: The “deardia.configure.new” only contains the default parameters, and the users can specify the parameters in this file.


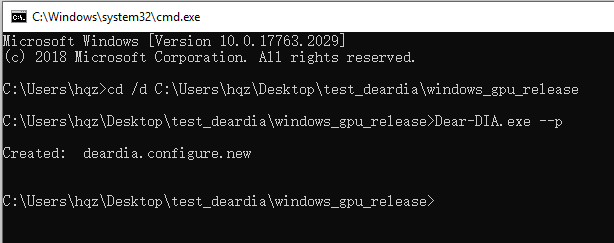


1. Enter the command such as “Dear-DIA.exe --config=deardia.configure.new --out_dir=C:\Users\hqz\Desktop\test_deardia\windows_gpu_release --input=C:\Users\hqz\Desktop\test_deardia\test_data\SW_100VW_CT_3.6s-Sample004.mzXML”


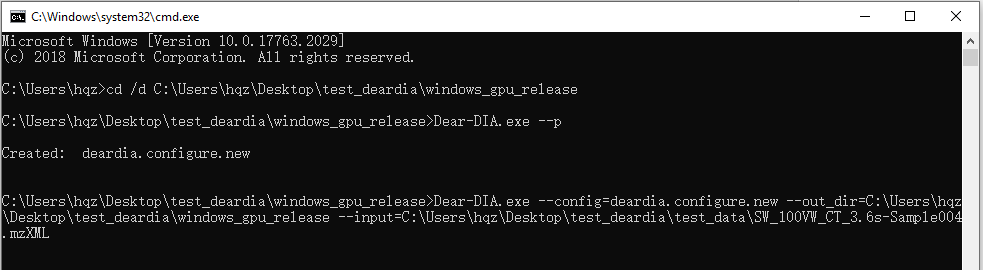


The description of the parameters of command line:

“--config”: the configure file of Dear-DIA.exe.

“--out_dir”: the output directory of MGF file.

“--input”: the input file of Dear-DIA.exe (mzXML format).

1. **Windows users compile the source code of Dear-DIA from scratch (CPU version).**
2. Compile the source code of Dear-DIA.

Dear-DIA requires the support of C++11. We have successfully compiled the source code of Dear-DIA on Visual Studio 2019 (version 16.8.9). So we use VS 2019 to demonstrate the compilation process.

1. Double click “Dear-DIA.sln” file to open the VS project.


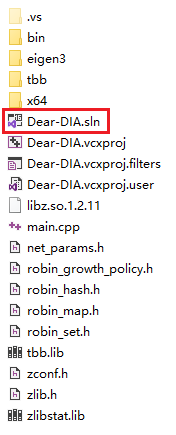


1. Click “Project” and choose “Dear-DIA Properties”.


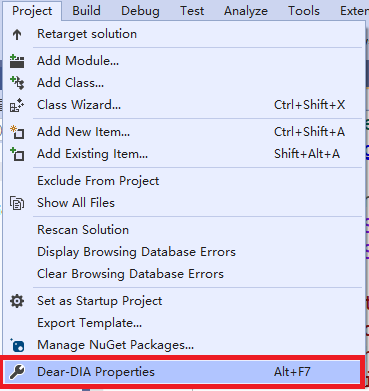


1. Click “VC++ Directories” and edit “Include Directories”. Add the “eigen3” in Dear-DIA folder to “Include Directories”.


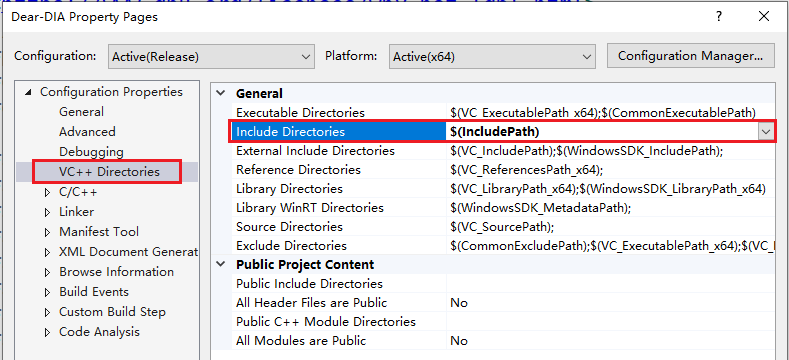


1. Choose the “Build” 🡪 “Rebuild Solution” option.


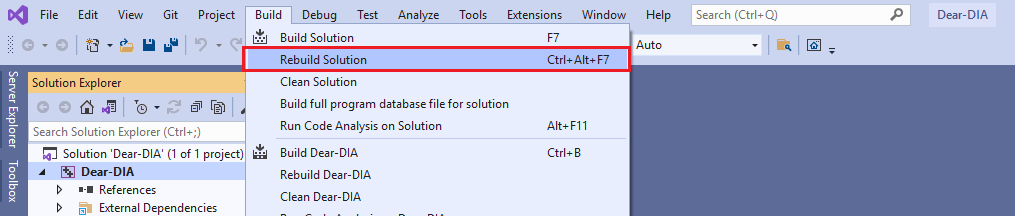


After the compilation is successful, the user will find the “Dear-DIA.exe” file in the “.\bin” directory.


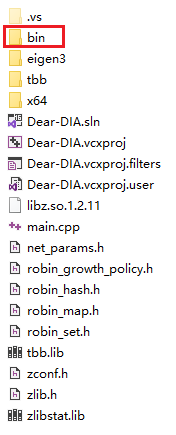


Note: The requirements of “Dear-DIA.exe” include: “vcomp140.dll” and “tbb.dll”.

The “tbb.dll” file has been placed in the “.\bin” folder in advance.


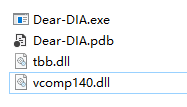


1. Run Dear-DIA using command line or GUI.
2. **Linux users compile the source code of Dear-DIA from scratch (CPU version).**
3. Download “oneTBB-tbb_2020.zip” and decompress this file. Threading Building Blocks (TBB) is the open source program, which can be download from GitHub repository <https://github.com/oneapi-src/oneTBB>.

Enter the decompressed command: “unzip oneTBB-tbb_2020.zip”.

Enter the command: “cd oneTBB-tbb_2020/” to entry the folder.


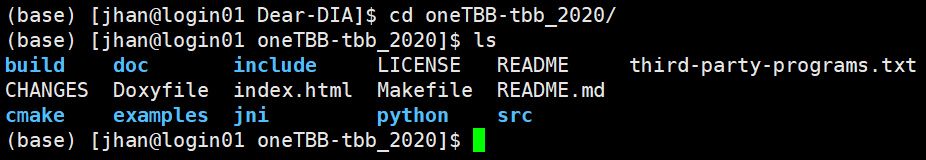


1. Compile TBB program.

Requirement: g++ >= 5.4.0

Enter the command: “make” to compile TBB program.


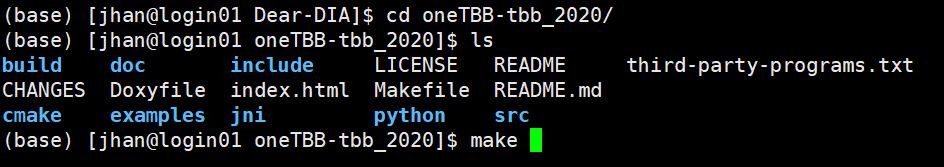


Find the “libtbb.so.2” file. This file may be placed in:

“. /build/linux_intel64_gcc_cc5.4.0_libc2.17_kernel3.10.0_release/”


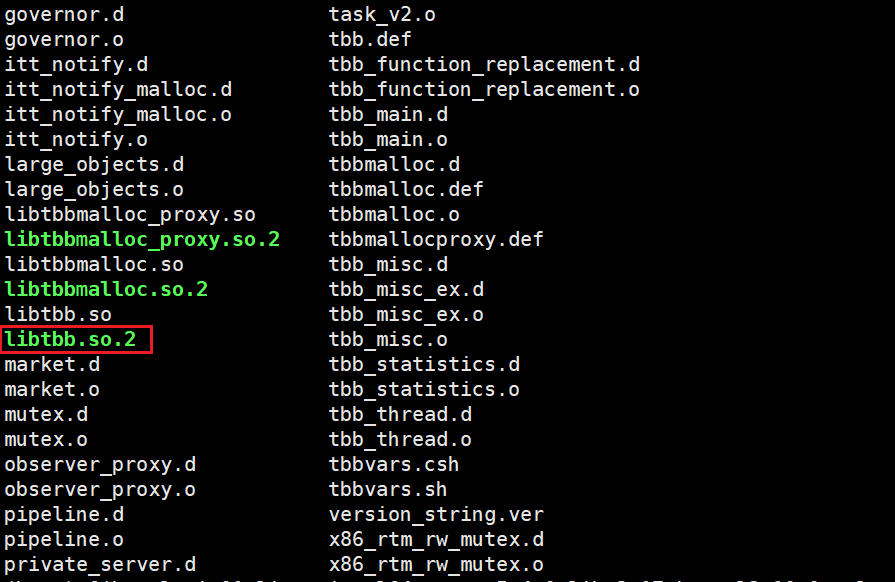


1. Copy “libtbb.so.2” file to “./oneTBB-tbb_2020” directory.


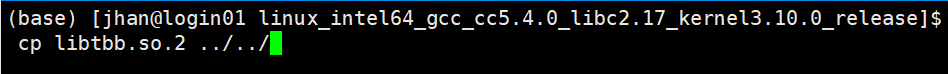

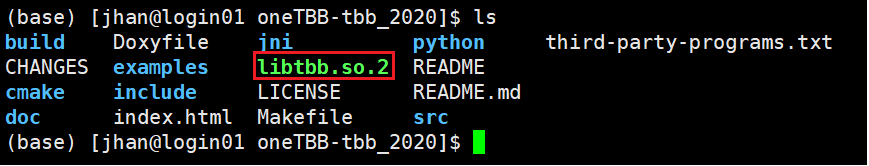


1. Go to the folder contained Dear-DIA source code.


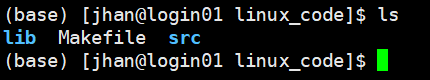


1. Copy “libtbb.so.2” file to “./lib” folder.


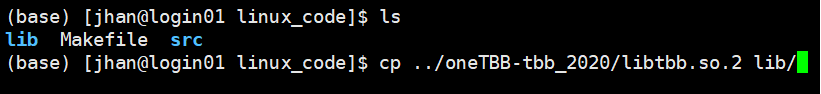

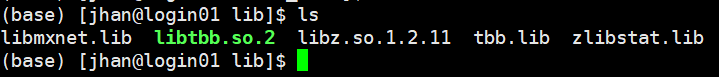


1. Go back to the folder of Dear-DIA source code.


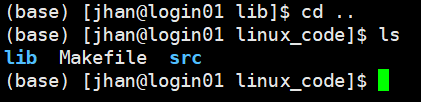


Enter the command: “vi Makefile” to open and modify Makefile.


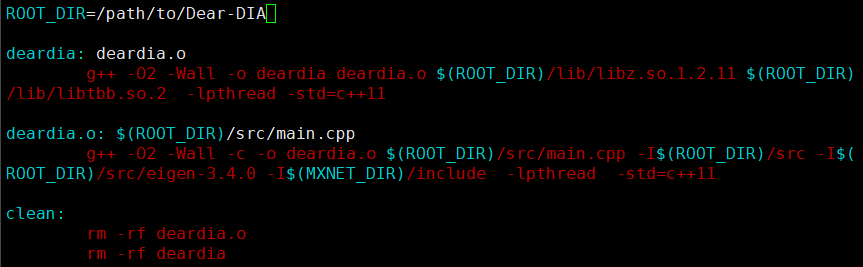


The users need to specify directories of “ROOT_DIR”. The “ROOT_DIR” indicates the directory of Dear-DIA source code.

1. Compile the source code of Dear-DIA.

Enter the command: “make” to compile source code. The current directory includes “Makefile”.


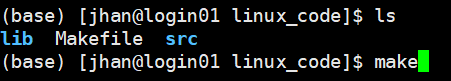

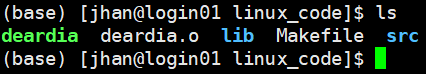


1. Copy “libtbb.so.2” file to the folder contained “deardia”.


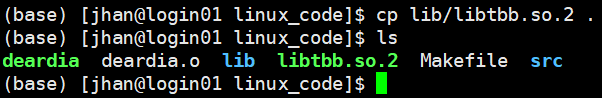


1. Run Dear-DIA.

Linux version of Dear-DIA only support running with command line. The running command of Linux is the same as running command of Windows.
